# Supplementary material for: A new species of the odorous frog genus Odorrana (Amphibia, Anura, Ranidae) from southwestern China
Source: PeerJ. 2018 Oct 4;6:e5695. doi: 10.7717/peerj.5695 (PMC6174872; doi:10.7717/peerj.5695)
Supplement: Supplemental Information 1 — Unit: mm. Abbreviations for the morphometric characters refer to Methods section. [file peerj-06-5695-s001.docx]

| **Species** | **Voucher** | **Sex** | **Locality** | **SVL** | **HDL** | **HDW** | **SL** | **ED** | **IOD** | **IND** | **NED** | **NSD** | **IFE** | **IAE** | **TYD** | **LAL** | **LW** | **THL** | **TL** | **TW** | **TFL** | **FL** | **FDW** | **DPW** |
| --- | --- | --- | --- | --- | --- | --- | --- | --- | --- | --- | --- | --- | --- | --- | --- | --- | --- | --- | --- | --- | --- | --- | --- | --- |
| *Odorrana kweichowensis* sp. nov. | CIBjs20150803006 | Female | Lengshuihe Nature Reserve, Jinsha Co., Guizhou Prov. China | 67.9 | 26.7 | 25.0 | 10.6 | 8.8 | 5.2 | 7.9 | 5.9 | 4.1 | 12.9 | 18.2 | 4.3 | 33.4 | 5.2 | 38.5 | 41.0 | 8.5 | 55.9 | 39.6 | 2.7 | 2.3 |
| *Odorrana kweichowensis* sp. nov. | CIBjs20150804001 | Female | Lengshuihe Nature Reserve, Jinsha Co., Guizhou Prov. China | 76.7 | 28.7 | 26.9 | 11.6 | 9.6 | 6.4 | 8.3 | 6.0 | 4.8 | 14.3 | 20.8 | 5.0 | 37.0 | 6.3 | 41.7 | 47.2 | 9.6 | 62.2 | 42.8 | 3.2 | 2.6 |
| *Odorrana kweichowensis* sp. nov. | CIBjs20150804005 | Female | Lengshuihe Nature Reserve, Jinsha Co., Guizhou Prov. China | 78.7 | 30.6 | 29.5 | 12.2 | 9.0 | 6.5 | 8.7 | 7.2 | 5.0 | 14.9 | 21.7 | 4.3 | 41.4 | 7.8 | 44.9 | 49.8 | 10.5 | 70.0 | 48.1 | 3.2 | 2.6 |
| *Odorrana kweichowensis* sp. nov. | CIBjs20150808020 | Female | Lengshuihe Nature Reserve, Jinsha Co., Guizhou Prov. China | 76.9 | 30.6 | 26.3 | 11.8 | 8.9 | 5.7 | 8.8 | 6.1 | 4.9 | 13.4 | 20.4 | 5.0 | 39.3 | 7.1 | 42.8 | 48.0 | 10.9 | 64.7 | 46.1 | 2.8 | 2.7 |
| *Odorrana kweichowensis* sp. nov. | CIBjs20150804004 | Female | Lengshuihe Nature Reserve, Jinsha Co., Guizhou Prov. China | 75.3 | 29.5 | 27.0 | 11.7 | 9.8 | 6.6 | 8.2 | 6.7 | 4.7 | 13.8 | 20.8 | 4.7 | 39.4 | 6.4 | 42.7 | 46.3 | 10.4 | 59.7 | 44.0 | 2.7 | 2.3 |
| *Odorrana kweichowensis* sp. nov. | CIBjs20150804006 | Female | Lengshuihe Nature Reserve, Jinsha Co., Guizhou Prov. China | 76.5 | 29.1 | 26.5 | 11.4 | 9.0 | 5.2 | 9.4 | 5.2 | 4.8 | 13.9 | 21.0 | 5.0 | 38.5 | 7.0 | 40.6 | 48.2 | 11.0 | 65.6 | 46.8 | 3.2 | 2.4 |
| *Odorrana kweichowensis* sp. nov. | CIBjs20150804003 | Female | Lengshuihe Nature Reserve, Jinsha Co., Guizhou Prov. China | 72.1 | 26.5 | 25.5 | 11.9 | 9.9 | 5.7 | 8.6 | 6.5 | 5.0 | 13.7 | 19.9 | 4.3 | 36.1 | 5.6 | 41.5 | 45.6 | 8.4 | 65.0 | 45.4 | 2.7 | 2.7 |
| *Odorrana kweichowensis* sp. nov. | CIBjs20150803008 | Female | Lengshuihe Nature Reserve, Jinsha Co., Guizhou Prov. China | 81.1 | 30.9 | 28.1 | 12.6 | 9.0 | 5.8 | 9.8 | 6.9 | 5.4 | 14.7 | 21.8 | 4.5 | 40.7 | 6.5 | 44.9 | 48.8 | 10.7 | 65.0 | 47.6 | 3.1 | 2.8 |
| *Odorrana kweichowensis* sp. nov. | CIBjs20150803007 | Female | Shilian, Meitan Co., Guizhou Prov. China | 78.1 | 30.1 | 28.2 | 11.3 | 9.5 | 5.3 | 8.2 | 6.1 | 4.9 | 14.0 | 21.2 | 4.6 | 38.1 | 6.8 | 44.0 | 48.4 | 10.6 | 65.3 | 45.7 | 3.5 | 3.1 |
| *Odorrana kweichowensis* sp. nov. | CIBGYU20130917003 | Female | Shilian, Meitan Co., Guizhou Prov. China | 78.6 | 29.0 | 27.3 | 12.1 | 9.4 | 5.3 | 8.6 | 6.5 | 4.9 | 14.1 | 19.7 | 5.2 | 37.4 | 6.5 | 42.1 | 46.4 | 11.2 | 62.1 | 44.5 | 2.9 | 2.9 |
| *Odorrana kweichowensis* sp. nov. | CIBGYU20130917006 | Female | Shilian, Meitan Co., Guizhou Prov. China | 67.6 | 25.6 | 23.7 | 10.9 | 8.0 | 5.2 | 7.7 | 5.2 | 4.5 | 12.5 | 16.9 | 4.7 | 33.8 | 5.8 | 37.1 | 42.7 | 8.6 | 56.5 | 37.9 | 2.5 | 2.4 |
| *Odorrana kweichowensis* sp. nov. | CIBGYU20130917002 | Female | Shilian, Meitan Co., Guizhou Prov. China | 64.7 | 26.2 | 23.3 | 11.0 | 7.9 | 5.5 | 7.7 | 5.7 | 3.9 | 12.6 | 18.0 | 4.7 | 35.2 | 5.2 | 35.8 | 43.2 | 8.3 | 58.0 | 40.3 | 2.4 | 2.1 |
| *Odorrana kweichowensis* sp. nov. | CIBCGYU20130917001 | Female | Xieba, Zhengan Co., Guizhou Prov. China | 75.6 | 27.6 | 26.3 | 12.1 | 8.0 | 4.8 | 8.5 | 6.0 | 4.8 | 13.6 | 19.2 | 5.1 | 38.8 | 7.1 | 41.2 | 49.2 | 10.5 | 63.5 | 45.4 | 2.5 | 2.8 |
| *Odorrana kweichowensis* sp. nov. | CIBGYU20130921003 | Female | Xieba, Zhengan Co., Guizhou Prov. China | 71.0 | 27.0 | 26.4 | 11.3 | 8.8 | 6.5 | 8.1 | 6.2 | 5.6 | 13.2 | 17.7 | 4.4 | 37.3 | 5.6 | 38.9 | 43.3 | 9.2 | 56.7 | 41.4 | 2.6 | 2.3 |
| *Odorrana kweichowensis* sp. nov. | CIBGYU20130921001 | Female | Xieba, Zhengan Co., Guizhou Prov. China | 74.4 | 27.9 | 27.0 | 11.4 | 7.7 | 5.9 | 8.2 | 6.8 | 4.4 | 13.7 | 18.9 | 4.7 | 37.8 | 5.4 | 41.3 | 46.8 | 9.2 | 62.3 | 43.2 | 2.9 | 2.3 |
| *Odorrana kweichowensis* sp. nov. | CIBGYU20130921002 | Female | Lengshuihe Nature Reserve, Jinsha Co., Guizhou Prov. China | 62.4 | 24.4 | 21.5 | 9.8 | 7.2 | 5.5 | 6.7 | 5.4 | 4.7 | 11.3 | 16.7 | 4.5 | 31.7 | 5.6 | 32.8 | 39.3 | 7.9 | 51.9 | 35.2 | 2.3 | 2.0 |
| *Odorrana kweichowensis* sp. nov. | CIBjs20150803004 | Male | Lengshuihe Nature Reserve, Jinsha Co., Guizhou Prov. China | 43.3 | 17.3 | 15.7 | 6.5 | 5.9 | 3.5 | 4.8 | 3.7 | 2.5 | 8.6 | 13.6 | 3.8 | 24.3 | 3.9 | 25.2 | 28.8 | 5.5 | 38.7 | 26.8 | 1.9 | 1.8 |
| *Odorrana kweichowensis* sp. nov. | CIBjs20150803002 | Male | Lengshuihe Nature Reserve, Jinsha Co., Guizhou Prov. China | 42.5 | 17.1 | 15.0 | 6.5 | 5.9 | 3.0 | 4.7 | 3.7 | 3.0 | 8.9 | 12.4 | 4.0 | 20.7 | 4.3 | 24.6 | 24.6 | 5.4 | 33.5 | 24.7 | 1.4 | 1.5 |
| *Odorrana kweichowensis* sp. nov. | CIBjs20150803001 | Male | Lengshuihe Nature Reserve, Jinsha Co., Guizhou Prov. China | 43.0 | 17.2 | 14.4 | 7.0 | 5.9 | 3.0 | 4.8 | 4.0 | 3.0 | 8.4 | 12.7 | 4.0 | 21.7 | 4.0 | 25.1 | 25.6 | 5.6 | 35.6 | 24.9 | 1.5 | 1.6 |
| *Odorrana kweichowensis* sp. nov. | CIBjs20150803005 | Male | Lengshuihe Nature Reserve, Jinsha Co., Guizhou Prov. China | 36.2 | 14.3 | 12.5 | 5.5 | 5.0 | 2.7 | 4.5 | 3.1 | 1.8 | 7.6 | 10.2 | 2.9 | 20.2 | 3.7 | 19.6 | 22.7 | 4.4 | 30.1 | 21.1 | 1.2 | 1.2 |
| *Odorrana kweichowensis* sp. nov. | CIBjs20150803003 | Male | Lengshuihe Nature Reserve, Jinsha Co., Guizhou Prov. China | 40.8 | 15.8 | 14.6 | 6.7 | 5.6 | 3.2 | 5.1 | 3.4 | 2.9 | 8.4 | 12.5 | 4.0 | 21.6 | 4.4 | 21.4 | 24.3 | 4.9 | 34.6 | 24.5 | 1.6 | 1.4 |
| *Odorrana kweichowensis* sp. nov. | CIBjs20150804002 | Male | Shilian, Meitan Co., Guizhou Prov. China | 42.1 | 15.8 | 15.0 | 7.0 | 5.6 | 2.6 | 5.4 | 4.0 | 2.8 | 8.4 | 12.8 | 4.2 | 20.8 | 4.0 | 23.6 | 26.3 | 5.1 | 36.1 | 24.0 | 1.7 | 1.4 |
| *Odorrana kweichowensis* sp. nov. | CIBGYU20130917005 | Male | Shilian, Meitan Co., Guizhou Prov. China | 38.7 | 14.1 | 13.4 | 6.0 | 4.8 | 3.4 | 4.9 | 3.2 | 2.6 | 7.3 | 10.8 | 4.0 | 20.3 | 3.9 | 21.7 | 24.1 | 4.5 | 34.6 | 22.9 | 1.4 | 1.4 |
| *Odorrana kweichowensis* sp. nov. | CIBGYU20130917004 | Male | Shilian, Meitan Co., Guizhou Prov. China | 41.4 | 15.9 | 15.6 | 6.3 | 6.2 | 2.7 | 5.8 | 3.5 | 2.7 | 8.2 | 12.4 | 4.5 | 19.0 | 3.6 | 21.9 | 22.8 | 5.6 | 32.4 | 23.4 | 1.5 | 1.3 |
| *Odorrana kweichowensis* sp. nov. | CIBGYU20130917007 | Male | Badagongshan Nature Reserve, Sangzhi Co., Hunan Prov. China | 40.5 | 14.9 | 14.1 | 6.1 | 5.2 | 2.6 | 4.6 | 3.8 | 2.9 | 7.2 | 11.4 | 4.0 | 21.4 | 3.2 | 21.6 | 24.1 | 5.0 | 32.7 | 20.2 | 1.4 | 1.4 |
| *O. schmackeri* | CIB130530 | Female | Badagongshan Nature Reserve, Sangzhi Co., Hunan Prov. China | 78.4 | 28.5 | 27.1 | 11.4 | 9.1 | 6.5 | 8.5 | 6.8 | 5.0 | 14.5 | 22.1 | 5.0 | 36.0 | 6.1 | 41.4 | 45.2 | 9.8 | 60.3 | 42.2 | 2.8 | 2.6 |
| *O. schmackeri* | CIBsz2012061905 | Female | Badagongshan Nature Reserve, Sangzhi Co., Hunan Prov. China | 72.1 | 27.1 | 26.1 | 11.5 | 9.5 | 4.8 | 8.2 | 6.8 | 6.3 | 13.2 | 21.3 | 5.3 | 36.0 | 6.7 | 38.2 | 44.7 | 10.5 | 61.4 | 42.8 | 2.6 | 2.4 |
| *O. schmackeri* | CIBsz2012061906 | Female | Badagongshan Nature Reserve, Sangzhi Co., Hunan Prov. China | 80.4 | 32.3 | 27.5 | 12.6 | 9.7 | 6.6 | 8.5 | 7.0 | 6.2 | 14.7 | 22.2 | 5.2 | 36.6 | 7.2 | 41.2 | 46.2 | 11.2 | 63.5 | 42.6 | 3.2 | 2.9 |
| *O. schmackeri* | CIBsz2012062013 | Female | Badagongshan Nature Reserve, Sangzhi Co., Hunan Prov. China | 74.9 | 30.2 | 25.7 | 11.2 | 8.3 | 5.3 | 8.7 | 5.8 | 5.2 | 13.3 | 20.1 | 4.9 | 35.0 | 5.8 | 37.7 | 43.0 | 9.6 | 61.1 | 42.0 | 2.5 | 2.3 |
| *O. schmackeri* | CIB130529 | Male | Badagongshan Nature Reserve, Sangzhi Co., Hunan Prov. China | 43.4 | 17.4 | 15.0 | 7.3 | 6.1 | 3.7 | 4.8 | 3.8 | 2.1 | 8.2 | 13.3 | 4.6 | 20.8 | 5.0 | 22.3 | 25.0 | 6.0 | 31.9 | 23.9 | 1.5 | 1.4 |
| *O. schmackeri* | CIB130531 | Male | Badagongshan Nature Reserve, Sangzhi Co., Hunan Prov. China | 43.2 | 17.7 | 15.8 | 7.2 | 6.9 | 4.1 | 5.0 | 4.2 | 3.3 | 8.6 | 13.7 | 4.5 | 20.3 | 4.2 | 22.0 | 24.0 | 5.8 | 33.4 | 23.9 | 1.5 | 1.5 |
| *O. schmackeri* | CIB130532 | Male | Badagongshan Nature Reserve, Sangzhi Co., Hunan Prov. China | 42.4 | 17.0 | 14.2 | 6.8 | 6.0 | 3.3 | 4.5 | 4.0 | 2.6 | 8.0 | 12.2 | 3.7 | 20.5 | 4.2 | 22.9 | 24.6 | 5.0 | 33.5 | 23.3 | 1.4 | 1.4 |
| *O. schmackeri* | CIB130533 | Male | Badagongshan Nature Reserve, Sangzhi Co., Hunan Prov. China | 42.7 | 17.9 | 15.5 | 7.1 | 3.6 | 3.6 | 4.7 | 3.9 | 2.6 | 8.5 | 12.6 | 4.1 | 22.2 | 4.2 | 24.6 | 25.0 | 5.6 | 36.4 | 24.6 | 1.4 | 1.3 |
| *O. schmackeri* | CIBsz2012061902 | Male | Badagongshan Nature Reserve, Sangzhi Co., Hunan Prov. China | 40.2 | 17.0 | 15.2 | 7.0 | 6.4 | 3.6 | 5.0 | 3.9 | 3.3 | 8.4 | 12.0 | 4.1 | 19.6 | 4.0 | 22.0 | 24.0 | 5.6 | 30.6 | 23.1 | 2.0 | 1.6 |
| *O. schmackeri* | CIBsz2012061908 | Male | Badagongshan Nature Reserve, Sangzhi Co., Hunan Prov. China | 37.9 | 15.4 | 13.9 | 6.1 | 5.3 | 3.2 | 4.5 | 3.3 | 2.7 | 7.2 | 11.8 | 3.3 | 19.7 | 3.8 | 20.8 | 23.4 | 5.2 | 32.5 | 22.7 | 1.6 | 1.4 |
| *O. schmackeri* | CIBsz2012061907 | Male | Badagongshan Nature Reserve, Sangzhi Co., Hunan Prov. China | 41.4 | 18.0 | 15.1 | 7.1 | 6.0 | 3.6 | 5.1 | 3.5 | 3.5 | 8.7 | 13.3 | 4.2 | 19.7 | 4.5 | 22.1 | 24.6 | 5.7 | 34.2 | 23.4 | 1.9 | 1.7 |
| *O. schmackeri* | CIBsz2012061903 | Male | Badagongshan Nature Reserve, Sangzhi Co., Hunan Prov. China | 41.6 | 17.2 | 14.5 | 6.7 | 6.4 | 3.7 | 5.3 | 3.6 | 3.3 | 7.9 | 13.0 | 4.5 | 20.7 | 4.8 | 21.8 | 23.8 | 5.0 | 33.1 | 23.7 | 1.9 | 1.8 |
| *O. schmackeri* | CIBsz2012061901 | Male | Badagongshan Nature Reserve, Sangzhi Co., Hunan Prov. China | 44.8 | 18.6 | 16.3 | 7.4 | 6.2 | 3.6 | 5.0 | 3.9 | 3.3 | 8.8 | 14.2 | 5.2 | 21.1 | 5.1 | 22.4 | 25.2 | 6.3 | 34.8 | 25.0 | 1.8 | 1.7 |
| *O. schmackeri* | CIBsz2012062003 | Male | Badagongshan Nature Reserve, Sangzhi Co., Hunan Prov. China | 42.6 | 17.3 | 15.1 | 7.2 | 6.3 | 3.2 | 5.0 | 3.5 | 3.2 | 8.4 | 12.9 | 4.3 | 21.0 | 5.1 | 22.6 | 24.9 | 5.7 | 33.8 | 24.1 | 2.1 | 1.9 |
| *O. schmackeri* | CIBsz2012062012 | Male | Badagongshan Nature Reserve, Sangzhi Co., Hunan Prov. China | 43.1 | 17.2 | 15.2 | 7.0 | 5.8 | 3.2 | 5.2 | 4.0 | 3.0 | 8.5 | 13.5 | 4.0 | 21.2 | 4.0 | 22.6 | 25.4 | 5.2 | 36.4 | 24.9 | 2.0 | 1.6 |
| *O. schmackeri* | CIBsz2012062011 | Male | Badagongshan Nature Reserve, Sangzhi Co., Hunan Prov. China | 40.0 | 16.3 | 14.5 | 6.8 | 6.4 | 2.9 | 4.8 | 3.2 | 2.8 | 8.1 | 12.8 | 4.0 | 19.2 | 4.3 | 20.7 | 23.3 | 5.9 | 31.6 | 22.6 | 1.7 | 1.4 |
| *O. schmackeri* | CIBsz2012062010 | Male | Badagongshan Nature Reserve, Sangzhi Co., Hunan Prov. China | 43.5 | 17.9 | 16.1 | 6.8 | 6.9 | 3.3 | 5.3 | 3.8 | 3.1 | 8.6 | 13.8 | 4.2 | 20.8 | 4.0 | 21.4 | 23.6 | 4.8 | 32.9 | 23.0 | 1.8 | 1.6 |
| *O. schmackeri* | CIBsz2012061904 | Male | Badagongshan Nature Reserve, Sangzhi Co., Hunan Prov. China | 42.1 | 18.3 | 15.6 | 7.0 | 5.6 | 3.6 | 5.0 | 3.8 | 2.9 | 8.2 | 12.7 | 4.2 | 20.2 | 5.0 | 22.7 | 23.8 | 5.6 | 32.9 | 21.5 | 2.0 | 2.0 |
| *O. schmackeri* | CIBsz2012062004 | Male | Mangshan Nature Reserve,Yichang Co., Hunan Prov. China | 40.7 | 16.5 | 15.7 | 7.6 | 6.1 | 3.7 | 5.2 | 3.9 | 2.7 | 8.0 | 13.0 | 4.1 | 22.4 | 5.2 | 22.7 | 21.7 | 6.1 | 36.7 | 24.3 | 2.1 | 1.8 |
| *O.huanggangensis* | CIBFJS20150614003 | Female | Yangxi Nature Reserve,Yinjiang Co., Guizhoug Prov. China | 77.9 | 28.8 | 28.3 | 11.8 | 9.2 | 6.9 | 8.9 | 6.4 | 5.1 | 13.5 | 20.6 | 5.4 | 38.0 | 6.1 | 41.3 | 41.8 | 10.7 | 61.3 | 44.5 | 3.0 | 3.1 |
| *O.huanggangensis* | CIBYJ20140514001 | Female | Yangxi Nature Reserve,Yinjiang Co., Guizhoug Prov. China | 82.1 | 31.1 | 30.2 | 12.9 | 9.4 | 5.8 | 8.7 | 6.0 | 5.4 | 14.7 | 22.6 | 5.1 | 37.8 | 7.1 | 41.4 | 48.8 | 12.5 | 65.1 | 44.5 | 3.2 | 3.3 |
| *O.huanggangensis* | CIBYJ20140514003 | Female | Nanling Nature Reserve, Ruyuan Co., Guangdong Prov. China | 88.6 | 32.1 | 31.9 | 13.3 | 9.9 | 6.6 | 9.8 | 6.6 | 5.3 | 14.3 | 23.1 | 5.7 | 41.0 | 8.5 | 46.2 | 53.1 | 13.0 | 71.4 | 48.2 | 3.4 | 3.0 |
| *O.huanggangensis* | CIBGD201108059 | Female | Nanling Nature Reserve, Ruyuan Co., Guangdong Prov. China | 81.8 | 28.7 | 27.8 | 13.7 | 10.4 | 5.6 | 9.5 | 6.7 | 5.6 | 16.4 | 21.4 | 5.6 | 37.6 | 7.3 | 42.7 | 48.9 | 11.4 | 68.1 | 48.3 | 3.1 | 2.9 |
| *O.huanggangensis* | CIBGD201108058 | Female | Nanling Nature Reserve, Ruyuan Co., Guangdong Prov. China | 76.9 | 27.5 | 26.9 | 13.0 | 9.8 | 4.7 | 9.2 | 7.1 | 6.0 | 15.3 | 20.3 | 5.3 | 36.0 | 6.7 | 42.1 | 45.8 | 10.8 | 62.7 | 42.6 | 2.8 | 2.5 |
| *O.huanggangensis* | CIBGD201108033 | Female | Nanling Nature Reserve, Ruyuan Co., Guangdong Prov. China | 81.4 | 29.1 | 27.6 | 12.7 | 10.0 | 6.1 | 9.0 | 6.8 | 6.0 | 15.5 | 22.0 | 5.1 | 38.8 | 7.2 | 44.0 | 49.4 | 11.2 | 65.3 | 45.8 | 3.1 | 2.7 |
| *O.huanggangensis* | CIBGD201108028 | Female | Nanling Nature Reserve, Ruyuan Co., Guangdong Prov. China | 74.6 | 27.5 | 27.3 | 10.4 | 9.5 | 5.5 | 8.6 | 6.3 | 5.9 | 13.9 | 20.4 | 4.8 | 34.3 | 5.7 | 40.3 | 45.9 | 10.8 | 62.8 | 44.0 | 2.8 | 2.8 |
| *O.huanggangensis* | CIBGD2011080? | Female | Leigongshan Nature Reserve, Leishan Co., Guizhou Prov. China | 79.1 | 30.9 | 28.7 | 12.5 | 10.0 | 5.8 | 8.8 | 6.6 | 5.3 | 15.7 | 22.4 | 4.7 | 36.1 | 6.5 | 40.4 | 47.0 | 12.0 | 62.9 | 43.9 | 3.0 | 2.8 |
| *O.huanggangensis* | CIBLGS20151112303 | Female | Leigongshan Nature Reserve, Leishan Co., Guizhou Prov. China | 70.1 | 28.0 | 25.9 | 11.4 | 8.1 | 5.2 | 8.6 | 6.5 | 5.2 | 13.6 | 20.4 | 5.1 | 33.6 | 6.8 | 37.4 | 42.6 | 9.3 | 58.0 | 41.9 | 2.8 | 2.4 |
| *O.huanggangensis* | CIBLGS2015052204 | Female | Fanjingshan Nature Reserve, Jiangkou Co., Guizhou Prov. China | 71.5 | 25.6 | 25.0 | 10.8 | 7.6 | 4.4 | 8.2 | 5.8 | 4.2 | 13.0 | 19.2 | 4.8 | 32.5 | 5.2 | 38.0 | 42.2 | 9.7 | 55.7 | 38.4 | 2.7 | 2.1 |
| *O.huanggangensis* | CIBFJS20150614001 | Male | Fanjingshan Nature Reserve, Jiangkou Co., Guizhou Prov. China | 46.7 | 16.1 | 16.0 | 7.1 | 6.1 | 3.9 | 4.8 | 4.2 | 3.3 | 8.3 | 14.6 | 4.4 | 21.9 | 5.0 | 24.0 | 27.0 | 5.5 | 33.6 | 25.0 | 2.0 | 1.9 |
| *O.huanggangensis* | CIBFJS20150614002 | Male | Leigongshan Nature Reserve, Leishan Co., Guizhou Prov. China | 43.6 | 16.8 | 15.9 | 7.2 | 5.4 | 3.8 | 4.7 | 3.0 | 3.9 | 9.0 | 14.4 | 4.4 | 21.8 | 4.7 | 23.0 | 25.9 | 5.7 | 31.7 | 23.6 | 2.1 | 1.8 |
| *O.huanggangensis* | CIBLS20141004003 | Male | Leigongshan Nature Reserve, Leishan Co., Guizhou Prov. China | 41.7 | 16.0 | 14.4 | 6.2 | 5.9 | 3.3 | 4.5 | 3.3 | 2.6 | 7.5 | 13.1 | 3.3 | 19.2 | 4.4 | 22.4 | 25.2 | 4.7 | 31.0 | 22.0 | 1.6 | 1.2 |
| *O.huanggangensis* | CIBLS20141004001 | Male | Yangxi Nature Reserve,Yinjiang Co., Guizhoug Prov. China | 43.3 | 16.1 | 14.8 | 6.2 | 5.7 | 3.0 | 4.5 | 3.8 | 2.6 | 7.9 | 12.5 | 4.3 | 18.9 | 3.1 | 21.7 | 23.1 | 4.8 | 30.4 | 21.5 | 1.2 | 1.3 |
| *O.huanggangensis* | CIBYJ20140514002 | Male | Nanling Nature Reserve, Ruyuan Co., Guangdong Prov. China | 38.0 | 14.2 | 12.7 | 5.9 | 4.4 | 3.0 | 4.1 | 3.3 | 2.4 | 7.3 | 10.6 | 3.4 | 18.5 | 3.1 | 19.6 | 22.6 | 5.1 | 29.3 | 20.4 | 1.2 | 1.0 |
| *O.huanggangensis* | CIBGD201108031 | Male | Nanling Nature Reserve, Ruyuan Co., Guangdong Prov. China | 42.3 | 18.0 | 15.5 | 7.4 | 6.5 | 3.3 | 5.3 | 3.9 | 3.3 | 8.6 | 13.2 | 4.0 | 21.1 | 4.3 | 23.0 | 25.3 | 5.3 | 35.9 | 25.9 | 1.6 | 1.3 |
| *O.huanggangensis* | CIBGD201108034 | Male | Nanling Nature Reserve, Ruyuan Co., Guangdong Prov. China | 39.6 | 15.0 | 15.0 | 6.8 | 6.2 | 3.0 | 4.2 | 3.5 | 3.0 | 8.5 | 13.4 | 4.0 | 20.6 | 4.7 | 21.7 | 23.8 | 5.9 | 34.2 | 24.5 | 1.7 | 1.6 |
| *O.huanggangensis* | CIBGD201108056 | Male | Nanling Nature Reserve, Ruyuan Co., Guangdong Prov. China | 44.0 | 17.7 | 15.6 | 7.2 | 7.3 | 3.0 | 5.1 | 4.6 | 3.3 | 8.6 | 13.6 | 4.2 | 23.1 | 5.5 | 24.4 | 27.6 | 6.2 | 37.7 | 25.9 | 1.8 | 1.7 |
| *O.huanggangensis* | CIBGD201108055 | Male | Nanling Nature Reserve, Ruyuan Co., Guangdong Prov. China | 42.6 | 17.4 | 15.4 | 7.2 | 6.8 | 3.0 | 4.8 | 3.6 | 3.3 | 8.5 | 13.2 | 4.0 | 20.3 | 4.4 | 21.7 | 23.7 | 5.9 | 33.4 | 23.6 | 1.8 | 1.4 |
| *O.huanggangensis* | CIBGD201108030 | Male | Nanling Nature Reserve, Ruyuan Co., Guangdong Prov. China | 41.9 | 17.4 | 14.9 | 7.1 | 6.5 | 3.0 | 4.9 | 3.3 | 2.9 | 7.7 | 13.0 | 3.4 | 20.2 | 4.1 | 22.0 | 24.3 | 5.8 | 33.1 | 24.5 | 1.4 | 1.4 |
| *O.huanggangensis* | CIBGD201108057 | Male | Nanling Nature Reserve, Ruyuan Co., Guangdong Prov. China | 38.7 | 15.3 | 13.8 | 6.7 | 5.6 | 2.7 | 4.5 | 3.7 | 2.9 | 7.8 | 12.5 | 3.2 | 19.8 | 3.9 | 21.8 | 23.8 | 5.0 | 29.9 | 22.2 | 1.5 | 1.2 |
| *O.huanggangensis* | CIBGD201108186 | Male | Mangshan Nature Reserve,Yichang Co., Hunan Prov. China | 41.8 | 16.7 | 15.0 | 7.1 | 6.0 | 3.3 | 5.1 | 3.5 | 3.2 | 7.9 | 12.7 | 4.0 | 20.1 | 4.6 | 23.1 | 25.3 | 5.7 | 35.6 | 23.2 | 1.5 | 1.5 |
| *O.huanggangensis* | CIBHN20110187 | Male | Mangshan Nature Reserve,Yichang Co., Hunan Prov. China | 44.1 | 17.4 | 15.4 | 7.0 | 5.7 | 3.5 | 5.3 | 3.9 | 3.4 | 8.7 | 13.1 | 4.0 | 21.7 | 4.7 | 23.5 | 25.5 | 5.8 | 34.8 | 24.0 | 1.7 | 1.5 |
