# Supplementary material for: A new species of the odorous frog genus Odorrana (Amphibia, Anura, Ranidae) from southwestern China
Source: PeerJ. 2018 Oct 4;6:e5695. doi: 10.7717/peerj.5695 (PMC6174872; doi:10.7717/peerj.5695)
Supplement: Supplemental Information 2 — “*” denoted the sequences downloaded from GenBank. [file peerj-06-5695-s002.docx]

| **Sample No.** | **Species** | **Locality** | **Voucher** | **GenBank Accession number** | | | | |
| --- | --- | --- | --- | --- | --- | --- | --- | --- |
|  |  |  |  | **12s** | **16s** | **ND2** | **DOLK** | **KCNF** |
| 1 | *Odorrana kweichowensis* sp. nov. | Shilian, Meitan Co., Guizhou Prov., China | CIBGYU20130917004 | MH193535 | MH193548 | MH193602 | MH193566 | MH193584 |
| 2 | *Odorrana kweichowensis* sp. nov. | Lengshuihe Nature Reserve, Jinsha Co., Guizhou Prov., China | CIBjs20150803008 | MH193538 | MH193552 | MH193606 | MH193570 | MH193588 |
| 3 | *Odorrana kweichowensis* sp. nov. | Shilian, Meitan Co., Guizhou Prov., China | CIBGYU20130921001 | MH193537 | MH193550 | MH193604 | MH193568 | MH193586 |
| 4 | *Odorrana kweichowensis* sp. nov. | Xieba, Zhengan Co., Guizhou Prov., China | CIBGYU20130917005 | MH193536 | MH193549 | MH193603 | MH193567 | MH193585 |
| 5 | *Odorrana kweichowensis* sp. nov. | Lengshuihe Nature Reserve, Jinsha Co., Guizhou Prov., China | CIBjs20171014001 | MH193539 | MH193551 | MH193605 | MH193569 | MH193587 |
| 6 | *Odorrana schmackeri* | Houhe Nature Reserve, Changyang Co., Hubei Prov. , China | CIBLC2010092 | MH193546 | MH193558 | – | MH193574 | MH193592 |
| 7 | *Odorrana schmackeri* | Houhe Nature Reserve, Changyang Co., Hubei Prov., China | CIBLC2010097 | MH193547 | MH193559 | – | MH193575 | MH193593 |
| 8 | *Odorrana schmackeri* | Yichang City, Hubei Prov., China | HNNU0908II349 | KF185011*** | KF185047*** | – | – | – |
| 9 | *Odorrana schmackeri* | Badagongshan Nature Reserve, Sangzhi Co., Hunan Prov., China | CIB20130531 | MH193543 | MH193555 | MH193609 | MH193571 | MH193589 |
| 10 | *Odorrana schmackeri* | Badagongshan Nature Reserve, Sangzhi Co., Hunan Prov., China | CIB20130533 | MH193545 | MH193557 | MH193611 | MH193573 | MH193591 |
| 11 | *Odorrana schmackeri* | Badagongshan Nature Reserve, Sangzhi Co., Hunan Prov., China | CIB20130532 | MH193544 | MH193556 | MH193610 | MH193572 | MH193590 |
| 12 | *Odorrana tiannanensis* | Hekou Co., Yunnan Prov., China | HNNUHK001 | KF185008*** | KF185044*** | – | – | – |
| 13 | *Odorrana bacboensis* | Khe Moi, Nghe An,Vietnam | – | AF206099*** | DQ650569*** | – | – | – |
| 14 | *Odorrana hainanensis* | Wuzhishan city, Hainan Prov., China | HNNU0606105 | KF184996*** | KF185032*** | – | – | – |
| 15 | *Odorrana fengkaiensis* | Shiwanshan city, Guangxi Prov., China | HNNU2957k | KF184997*** | KF185033*** | – | – | – |
| 16 | *Odorrana nanjiangensis* | Yichang Co., Hubei Prov., China | HNNU1007I061 | KF185005* | KF185041* |  |  |  |
| 17 | *Odorranahe hejiangensis* | Hejiang Co., Sichuan Prov., China | HNNU1007I202 | KF185016* | KF185052* |  |  |  |
| 18 | *Odorrana huanggangensis* | Nanling Nature Reserve, Ruyuan Co., Guangdong Prov., China | CIBGD201108030 | MH193531 | MH193563 | MH193613 | MH193577 | MH193595 |
| 19 | *Odorrana huanggangensis* | Fanjingshan Nature Reserve, Jiangkou Co., Guizhou Prov., China | CIBFJS20150502002 | MH193532 | MH193565 | MH193614 | MH193578 | MH193596 |
| 20 | *Odorrana huanggangensis* | Wuyishan Nature Reserve, Fujian Prov., China | HNNU0607001 | KF185023*** | KF185059*** | – | – | – |
| 21 | *Odorrana huanggangensis* | Leigongshan Nature Reserve, Leishan Co., Guizhou Prov., China | CIBLS20140818005 | MH193530 | MH193564 | MH193612 | MH193576 | MH193594 |
| 22 | *Odorrana tianmuii* | Lin’an area , Zhejiang Prov., China | HNNU707071 | KF185004*** | KF185040*** | – | – | – |
| 23 | *Odorrana narina* | Okinawa Island, Ryukyu | – | AB511287*** | AB511287*** | – | – | – |
| 24 | *Odorrana amamiensis* | Tokunoshima, Ryukyu | – | AB200923*** | AB200947*** | – | – | – |
| 25 | *Odorrana supranarina* | Iriomotejima, Ryukyu | – | AB200926*** | AB200950*** | – | – | – |
| 26 | *Odorrana swinhoana* | Nantou Co., Taiwan Prov., China | HNNUTW9 | KF185010*** | KF185046*** | – | – | – |
| 27 | *Odorrana utsunomiyaorum* | Iriomotejima, Ryukyu | - | AB200928*** | AB200952*** | – | – | – |
| 28 | *Odorrana nasuta* | Wuzhishan city, Hainan Prov., China | HNNU051119 | KF185017*** | KF185053*** | – | – | – |
| 29 | *Odorrana versabilis* | Leigongshan Nature Reserve, Leishan CO., Guizhou Prov., China | HNNU003 | KF185019*** | KF185055*** | – | – | – |
| 30 | *Odorrana exiliversabilis* | Wuyishan city, Fujian Prov., China | HNNU0607032 | KF185020*** | KF185056*** | – | – | – |
| 31 | *Odorrana nasica* | HaTinh, Vietnam | – | DQ283345*** | DQ283345*** | – | – | – |
| 32 | *Odorrana tormota* | Huangshan city, Anhui Prov., China | – | DQ835616*** | DQ835616*** | – | – | – |
| 33 | *Odorrana leporipes* | Shaoguan city, Guangdong Prov., China | HNNU1008I099 | KF185000*** | KF185036*** | – | – | – |
| 34 | *Odorrana graminea* | Wuzhishan city, Hainan Prov., China | HNNU0606123 | KF185002*** | KF185038*** | – | – | – |
| 35 | *Odorrana chloronota* | Ha Giang, Vietnam | – | DQ283394*** | DQ283394*** | – | – | – |
| 36 | *Odorrana hosii* | Kuala Lumpur, Malaysia | – | AB511284*** | AB511284*** | – | – | – |
| 37 | *Odorrana banaorum* | Tram Lap, Vietnam | – | AF206106*** | AF206487*** | – | – | – |
| 38 | *Odorrana morafkai* | TramLap, Vietnam | – | AF206103*** | AF206484*** | – | – | – |
| 39 | *Odorrana ishikawae* | Amami Island, Ryukyu | – | AB511282*** | AB511282*** | – | – | – |
| 40 | *Odorrana grahami* | Kunming city, Yunnan Prov., China | HNNU1008II016 | KF185015*** | KF185051*** | – | – | – |
| 41 | *Odorrana junlianensis* | Junlian, Sichuan Prov., China | HNNU002JL | KF185022*** | KF185058*** | – | – | – |
| 42 | *Odorrana daorum* | Sa Pa,Vietnam | – | AF206101*** | AF206482*** | – | – | – |
| 43 | *Odorrana hmongorum* | Lao Cai, Vietnam | – | – | EU861559*** | – | – | – |
| 44 | *Odorrana andersonii* | Longchuan Co., Yunnan Prov., China | HNNU001YN | KF185021*** | KF185057*** | – | – | – |
| 45 | *Odorrana jingdongensis* | Jingdong Co., Yunan Prov., China | 20070711017 | KF185014*** | KF185050*** | – | – | – |
| 46 | *Odorrana kuangwuensis* | Nanjiang Co., Sichuan Prov., China | HNNU0908II185 | KF184998*** | KF185034*** | – | – | – |
| 47 | *Odorrana margaretae* | Emei city, Sichuan Prov., China | HNNU20050032 | KF184999*** | KF185035*** | – | – | – |
| 48 | *Odorrana wuchuanensis* | Wuchuan Co., Guizhou Prov., China | HNNU019L | KF185007*** | KF185043*** | – | – | – |
| 49 | *Odorrana yizhangensis* | Fanjingshan Nature Reserve, Jiangkou Co., Guizhou Prov., China | CIBFJS20150501004 | MH193541 | MH193561 | MH193616 | MH193583 | MH193600 |
| 50 | *Odorrana yizhangensis* | Fanjingshan Nature Reserve, Jiangkou Co., Guizhou Prov., China | CIBFJS20150501006 | MH193542 | MH193562 | MH193617 | MH193582 | MH193601 |
| 51 | *Odorrana yizhangensis* | Nanling Nature Reserve, Ruyuan Co., Guangdong Prov., China | CIBHN201108149 | MH193540 | MH193560 | MH193615 | MH193581 | MH193599 |
| 52 | *Odorrana yizhangensis* | Nanling Nature Reserve, Ruyuan Co., Guangdong Prov., China | HNNU1008I075 | KF185012*** | KF185048*** | – | – | – |
| 53 | *Odorrana lungshengensis* | Leigongshan Nature Reserve, Leishan Co., Guizhou Prov., China | CIBLS20140616006 | MH193534 | MH193554 | MH193608 | MH193580 | MH193598 |
| 54 | *Odorrana lungshengensis* | Leigongshan Nature Reserve, Leishan Co., Guizhou Prov., China | CIBLS20140616004 | MH193533 | MH193553 | MH193607 | MH193579 | MH193597 |
| 55 | *Odorrana lungshengensis* | Longsheng Co., Guangxi Prov., China | HNNU70028 | KF185018*** | KF185054*** | – | – | – |
| 56 | *Odorrana anlungensis* | Anlong Co., Guizhou Prov., China | HNNU1008I109 | KF185013*** | KF185049*** | – | – | – |
| 57 | *Odorrana chapaensis* | Lai Chau, Vietnam | – | DQ283372*** | DQ283372*** | – | – | – |
| 58 | *Hylarana guentheri* | Fuzhou city, Fujian Prov., China | HNNU060435 | KF185024*** | KF185060*** | – | – | – |
| 59 | *Hylarana spinulosa* | Wuzhishan city, Hainan Prov., China | HNNU051117 | KF185031*** | KF185067*** | – | – | – |
| 60 | *Babina daunchina* | Emeishan city, Sichuan Prov., China | HNNU20060103 | KF185029*** | KF185065*** | – | – | – |
| 61 | *Rana chensinensis* | Ningshan Co., Shanxi Prov., China | HNNU 20060359 | KF185025*** | KF185061*** | – | – | – |
| 62 | *Odorrana kweichowensis* sp. nov. | Kuankuoshui, Suiyang Co., Guizhou Prov., China | KKS01 |  |  | KP167566* |  |  |
| 63 | *Odorrana kweichowensis* sp. nov. | Kuankuoshui, Suiyang Co., Guizhou Prov., China | KKS15 |  |  | KP167567* |  |  |
| 64 | *Odorrana kweichowensis* sp. nov. | Lengshuihe, Jinsha Co., Guizhou Prov., China | LSH35 |  |  | KP167568* |  |  |
| 65 | *Odorrana schmackeri* | Gaojiayan, Yichang City, Hubei Prov., China | GJY02 |  |  | KP167551* |  |  |
| 66 | *Odorrana schmackeri* | Gaojiayan, Yichang City, Hubei Prov., China | GJY04 |  |  | KP167552* |  |  |
| 67 | *Odorrana schmackeri* | Gaojiayan, Yichang City, Hubei Prov., China | GJY12 |  |  | KP167553* |  |  |
| 68 | *Odorrana schmackeri* | Gaojiayan, Yichang City, Hubei Prov., China | GJY07 |  |  | KP167554* |  |  |
| 69 | *Odorrana schmackeri* | Gaojiayan, Yichang City, Hubei Prov., China | GJY01 |  |  | KP167555* |  |  |
| 70 | *Odorrana schmackeri* | Gaojiayan, Yichang City, Hubei Prov., China | GJY30 |  |  | KP167556* |  |  |
| 71 | *Odorrana schmackeri* | Gaojiayan, Yichang City, Hubei Prov., China | GJY27 |  |  | KP167557* |  |  |
| 72 | *Odorrana schmackeri* | Hupingshan, Shimen Co., Hunan Prov., China | HPS27 |  |  | KP167558* |  |  |
| 73 | *Odorrana schmackeri* | Hupingshan, Shimen Co., Hunan Prov., China | HPS24 |  |  | KP167559* |  |  |
| 74 | *Odorrana schmackeri* | Hupingshan, Shimen Co., Hunan Prov., China | HPS11 |  |  | KP167560* |  |  |
| 75 | *Odorrana schmackeri* | Hupingshan, Shimen Co., Hunan Prov., China | HPS29 |  |  | KP167561* |  |  |
| 76 | *Odorrana schmackeri* | Hupingshan, Shimen Co., Hunan Prov., China | HPS17 |  |  | KP167562* |  |  |
| 77 | *Odorrana schmackeri* | Hupingshan, Shimen Co., Hunan Prov., China | HPS02 |  |  | KP167563* |  |  |
| 78 | *Odorrana schmackeri* | Hupingshan, Shimen Co., Hunan Prov., China | HPS13 |  |  | KP167564* |  |  |
| 79 | *Odorrana schmackeri* | Hupingshan, Shimen Co., Hunan Prov., China | HPS07 |  |  | KP167565* |  |  |
| 80 | *Odorrana schmackeri* | Shennongjia, Shennongjia Forest Region, Hubei Prov., China | SNJ24 |  |  | KP167571* |  |  |
| 81 | *Odorrana schmackeri* | Funiushan, Nanzhao Co., Henan Prov., China | FNS03 |  |  | KP167572* |  |  |
| 82 | *Odorrana huanggangensis* | Daiyunshan, Dehua Co., Fujian Prov., China | DYS01 |  |  | KP167492* |  |  |
| 83 | *Odorrana huanggangensis* | Fanjingshan, Jiangkou Co., Guizhou Prov., China | FJS01 |  |  | KP167493* |  |  |
| 84 | *Odorrana huanggangensis* | Fanjingshan, Jiangkou Co., Guizhou Prov., China | FJS07 |  |  | KP167494* |  |  |
| 85 | *Odorrana huanggangensis* | Fanjingshan, Jiangkou Co., Guizhou Prov., China | FJS03 |  |  | KP167495* |  |  |
| 86 | *Odorrana huanggangensis* | Fanjingshan, Jiangkou Co., Guizhou Prov., China | FJS12 |  |  | KP167496* |  |  |
| 87 | *Odorrana huanggangensis* | Fanjingshan, Jiangkou Co., Guizhou Prov., China | FJS15 |  |  | KP167497* |  |  |
| 88 | *Odorrana huanggangensis* | Fanjingshan, Jiangkou Co., Guizhou Prov., China | FJS06 |  |  | KP167498* |  |  |
| 89 | *Odorrana huanggangensis* | Guposhan, Hezhou City, Guangxi Prov., China | GPS01 |  |  | KP167499* |  |  |
| 90 | *Odorrana huanggangensis* | Guposhan, Hezhou City, Guangxi Prov., China | GPS04 |  |  | KP167500* |  |  |
| 91 | *Odorrana huanggangensis* | Guposhan, Hezhou City, Guangxi Prov., China | GPS05 |  |  | KP167501* |  |  |
| 92 | *Odorrana huanggangensis* | Leigongshan, Leishan Co., Guizhou Prov., China | LGS21 |  |  | KP167507* |  |  |
| 93 | *Odorrana huanggangensis* | Leigongshan, Leishan Co., Guizhou Prov., China | LGS07 |  |  | KP167508* |  |  |
| 94 | *Odorrana huanggangensis* | Leigongshan, Leishan Co., Guizhou Prov., China | LGS06 |  |  | KP167509* |  |  |
| 95 | *Odorrana huanggangensis* | Leigongshan, Leishan Co., Guizhou Prov., China | LGS04 |  |  | KP167510* |  |  |
| 96 | *Odorrana huanggangensis* | Leigongshan, Leishan Co., Guizhou Prov., China | LGS30 |  |  | KP167511* |  |  |
| 97 | *Odorrana huanggangensis* | Leigongshan, Leishan Co., Guizhou Prov., China | LGS22 |  |  | KP167512* |  |  |
| 98 | *Odorrana huanggangensis* | Leigongshan, Leishan Co., Guizhou Prov., China | LGS03 |  |  | KP167513* |  |  |
| 99 | *Odorrana huanggangensis* | Leigongshan, Leishan Co., Guizhou Prov., China | LGS08 |  |  | KP167514* |  |  |
| 100 | *Odorrana huanggangensis* | Liuxihe, Conghua Co., Guangdong Prov., China | LXH01 |  |  | KP167515* |  |  |
| 101 | *Odorrana huanggangensis* | Liuxihe, Conghua Co., Guangdong Prov., China | LXH09 |  |  | KP167516* |  |  |
| 102 | *Odorrana huanggangensis* | Liuxihe, Conghua Co., Guangdong Prov., China | LXH06 |  |  | KP167517* |  |  |
| 103 | *Odorrana huanggangensis* | Liuxihe, Conghua Co., Guangdong Prov., China | LXH11 |  |  | KP167518* |  |  |
| 104 | *Odorrana huanggangensis* | Liuxihe, Conghua Co., Guangdong Prov., China | LXH04 |  |  | KP167519* |  |  |
| 105 | *Odorrana huanggangensis* | Mangshan, Yizhang Co., Hunan Prov., China | MS15 |  |  | KP167520* |  |  |
| 106 | *Odorrana huanggangensis* | Mangshan, Yizhang Co., Hunan Prov., China | MS12 |  |  | KP167521* |  |  |
| 107 | *Odorrana huanggangensis* | Mangshan, Yizhang Co., Hunan Prov., China | MS08 |  |  | KP167522* |  |  |
| 108 | *Odorrana huanggangensis* | Mangshan, Yizhang Co., Hunan Prov., China | MS07 |  |  | KP167523* |  |  |
| 109 | *Odorrana huanggangensis* | Mangshan, Yizhang Co., Hunan Prov., China | MS02 |  |  | KP167524* |  |  |
| 110 | *Odorrana huanggangensis* | Mangshan, Yizhang Co., Hunan Prov., China | MS09 |  |  | KP167525* |  |  |
| 111 | *Odorrana huanggangensis* | Mangshan, Yizhang Co., Hunan Prov., China | MS06 |  |  | KP167526* |  |  |
| 112 | *Odorrana huanggangensis* | Maoershan, Xing’an Co., Guangxi Prov., China | MES01 |  |  | KP167527* |  |  |
| 113 | *Odorrana huanggangensis* | Maoershan, Xing’an Co., Guangxi Prov., China | MES13 |  |  | KP167528* |  |  |
| 114 | *Odorrana huanggangensis* | Maoershan, Xing’an Co., Guangxi Prov., China | MES09 |  |  | KP167529* |  |  |
| 115 | *Odorrana huanggangensis* | Maoershan, Xing’an Co., Guangxi Prov., China | MES05 |  |  | KP167530* |  |  |
| 116 | *Odorrana huanggangensis* | Nanling, Ruyuan Co., Guangdong Prov., China | NL16 |  |  | KP167531* |  |  |
| 117 | *Odorrana huanggangensis* | Nanling, Ruyuan Co., Guangdong Prov., China | NL05 |  |  | KP167532* |  |  |
| 118 | *Odorrana huanggangensis* | Wuyishan, Wuyishan Co., Fujian Prov., China | WYS09 |  |  | KP167541* |  |  |
| 119 | *Odorrana huanggangensis* | Wuyishan, Wuyishan Co., Fujian Prov., China | WYS02 |  |  | KP167542* |  |  |
| 120 | *Odorrana huanggangensis* | Wuyishan, Wuyishan Co., Fujian Prov., China | WYS23 |  |  | KP167543* |  |  |
| 121 | *Odorrana huanggangensis* | Wuyishan, Wuyishan Co., Fujian Prov., China | WYS03 |  |  | KP167544* |  |  |
| 122 | *Odorrana huanggangensis* | Yinpingshan, Dongguan City, Guangdong Prov., China | YPS01 |  |  | KP167545* |  |  |
| 123 | *Odorrana huanggangensis* | Yinpingshan, Dongguan City, Guangdong Prov., China | YPS06 |  |  | KP167546* |  |  |
| 124 | *Odorrana huanggangensis* | Yinpingshan, Dongguan City, Guangdong Prov., China | YPS14 |  |  | KP167547* |  |  |
| 125 | *Odorrana huanggangensis* | Yinpingshan, Dongguan City, Guangdong Prov., China | YPS12 |  |  | KP167548* |  |  |
| 126 | *Odorrana huanggangensis* | Yinpingshan, Dongguan City, Guangdong Prov., China | YPS18 |  |  | KP167549* |  |  |
| 127 | *Odorrana huanggangensis* | Yinpingshan, Dongguan City, Guangdong Prov., China | YPS17 |  |  | KP167550* |  |  |
| 128 | *Odorrana tianmuii* | Sanchahe, Shitai Co., Anhui Prov., China | SCH17 |  |  | KP167485* |  |  |
| 129 | *Odorrana tianmuii* | Sanchahe, Shitai Co., Anhui Prov., China | SCH19 |  |  | KP167486* |  |  |
| 130 | *Odorrana tianmuii* | Sanchahe, Shitai Co., Anhui Prov., China | SCH18 |  |  | KP167487* |  |  |
| 131 | *Odorrana tianmuii* | Sanchahe, Shitai Co., Anhui Prov., China | SCH22 |  |  | KP167488* |  |  |
| 132 | *Odorrana tianmuii* | Jiuhuashan, Chizhou City, Prov., China | JHS09 |  |  | KP167489* |  |  |
| 133 | *Odorrana tianmuii* | Jiuhuashan, Chizhou City, Prov., China | JHS04 |  |  | KP167490* |  |  |
| 134 | *Odorrana tianmuii* | Jiuhuashan, Chizhou City, Prov., China | JHS07 |  |  | KP167491* |  |  |
| 135 | *Odorrana tianmuii* | Guniujiang, Qimen Co., Anhui Prov., China | GNJ18 |  |  | KP167502* |  |  |
| 136 | *Odorrana tianmuii* | Guniujiang, Qimen Co., Anhui Prov., China | GNJ27 |  |  | KP167503* |  |  |
| 137 | *Odorrana tianmuii* | Guniujiang, Qimen Co., Anhui Prov., China | GNJ10 |  |  | KP167504* |  |  |
| 138 | *Odorrana tianmuii* | Guniujiang, Qimen Co., Anhui Prov., China | GNJ06 |  |  | KP167505* |  |  |
| 139 | *Odorrana tianmuii* | Guniujiang, Qimen Co., Anhui Prov., China | GNJ21 |  |  | KP167506* |  |  |
| 140 | *Odorrana tianmuii* | Nanxijiang, Yongjia Co., Zhejiang Prov., China | NXJ01 |  |  | KP167533* |  |  |
| 141 | *Odorrana tianmuii* | Nanxijiang, Yongjia Co., Zhejiang Prov., China | NXJ14 |  |  | KP167534* |  |  |
| 142 | *Odorrana tianmuii* | Nanxijiang, Yongjia Co., Zhejiang Prov., China | NXJ03 |  |  | KP167535* |  |  |
| 143 | *Odorrana tianmuii* | Nanxijiang, Yongjia Co., Zhejiang Prov., China | NXJ07 |  |  | KP167536* |  |  |
| 144 | *Odorrana tianmuii* | Nanxijiang, Yongjia Co., Zhejiang Prov., China | NXJ08 |  |  | KP167537* |  |  |
| 145 | *Odorrana tianmuii* | Tianmushan, Lin’an Co., Zhejiang Prov., China | TMS07 |  |  | KP167538* |  |  |
| 146 | *Odorrana tianmuii* | Tianmushan, Lin’an Co., Zhejiang Prov., China | TMS03 |  |  | KP167539* |  |  |
| 147 | *Odorrana tianmuii* | Tianmushan, Lin’an Co., Zhejiang Prov., China | TMS10 |  |  | KP167540* |  |  |
| 148 | *Odorrana* sp1 | Lushan, Jiujiang City, Jiangxi Prov., China | LS01 |  |  | KP167569* |  |  |
| 149 | *Odorrana* sp1 | Wugongshan, Luxi Co., Jiangxi Prov., China | WGS01 |  |  | KP167576* |  |  |
| 150 | *Odorrana* sp1 | Wugongshan, Luxi Co., Jiangxi Prov., China | WGS03 |  |  | KP167577* |  |  |
| 151 | *Odorrana* sp2 | Shennongjia, Shennongjia Forest Region, Hubei Prov., China | SNJ01 |  |  | KP167570* |  |  |
| 152 | *Odorrana* sp3 | Funiushan, Nanzhao Co., Henan Prov., China | FNS02 |  |  | KP167573* |  |  |
| 153 | *Odorrana* sp3 | Funiushan, Nanzhao Co., Henan Prov., China | FNS07 |  |  | KP167574* |  |  |
| 154 | *Odorrana* sp3 | Funiushan, Nanzhao Co., Henan Prov., China | FNS01 |  |  | KP167575* |  |  |
