# Supplementary material for: A new species of the odorous frog genus Odorrana (Amphibia, Anura, Ranidae) from southwestern China
Source: PeerJ. 2018 Oct 4;6:e5695. doi: 10.7717/peerj.5695 (PMC6174872; doi:10.7717/peerj.5695)
Supplement: Supplemental Information 3 [file peerj-06-5695-s003.docx]

| **Gene** | **Identifiers** | **Sequence (5’ to 3’)** | **Sources** |
| --- | --- | --- | --- |
| 12S rRNA | P1 | CCAGGCTTTACACTTTATGC | Kocher *et al.*, 1989 |
|  | P2 | GCGATTAAGTTGGGTAACGC |  |
| 16S rRNA | P7 | CGCCTGTTTACCAAAAACAT | Simon *et al.*, 1994 |
|  | P8 | CCGGTCTGAACTCAGATCACGT |  |
| ND2 | Ile-LND2 | ATAGGGAGACTTATAGGGGTTC | Li *et al.*, 2015 |
|  | Asn-HDN2 | CTAAGTCATTACGGGATCGAGGCC |  |
| DOLK | DOLK_F1 | GARGTCATHGARGTNYTNGARGT | Shen *et al.*, 2013 |
|  | DOLK_R1 | GTYTTYTTDGTNCCNGGCCA |  |
|  | DOLK_F2 | AGGGTTTTCCCAGTCACGACCGMTGCTTYACHCCYGGNGARGC |  |
|  | DOLK_R2 | AGATAACAATTTCACACAGGGTGTCNCCTACNCCNACNGC |  |
| KCNF | KCNF1_F1 | GRGAYCCNGAYGCNTTYAARTG | Shen *et al.*, 2013 |
|  | KCNF1_R1 | GACRAARTTRTTDATDATNGGRTG |  |
|  | KCNF1_F2 | AGGGTTTTCCCAGTCACGACCAYATGAARAARGGNATHTGYCC |  |
|  | KCNF1_R2 | AGATAACAATTTCACACAGGGGGTADATRTCNCCRTANCCNAC |  |
