# Supplementary material for: A new species of the odorous frog genus Odorrana (Amphibia, Anura, Ranidae) from southwestern China
Source: PeerJ. 2018 Oct 4;6:e5695. doi: 10.7717/peerj.5695 (PMC6174872; doi:10.7717/peerj.5695)
Supplement: Supplemental Information 4 — Unit: mm. Abbreviations for the morphometric characters refer to Methods section. [file peerj-06-5695-s004.docx]

| **Voucher number** | **TOL** | **SVL** | **BH** | **BW** | **SS** | **SL** | **MW** | **TL** | **TH** | **TBW** | **Gosner’s stage** |
| --- | --- | --- | --- | --- | --- | --- | --- | --- | --- | --- | --- |
| CIBJS20171014001 | 35.0 | 13.1 | 4.1 | 5.0 | 7.6 | 3.4 | 2.7 | 23.3 | 5.4 | 1.9 | 29 |
| CIBJS20171014002 | 35.4 | 12.1 | 4.2 | 5.7 | 7.5 | 2.6 | 3.0 | 23.8 | 5.6 | 2.2 | 29 |
| CIBJS20171014003 | 35.5 | 14.5 | 3.7 | 5.3 | 7.6 | 3.5 | 3.0 | 22.7 | 6.3 | 2.0 | 29 |
| CIBJS20171014004 | 36.2 | 13.8 | 3.6 | 5.0 | 8.2 | 3.5 | 3.1 | 22.7 | 5.9 | 2.0 | 29 |
| CIBJS20171014005 | 35.9 | 13.5 | 4.1 | 6.2 | 7.7 | 3.5 | 2.7 | 23.4 | 6.0 | 2.2 | 28 |
| CIBJS20171014006 | 32.8 | 12.4 | 4.3 | 5.4 | 7.6 | 3.5 | 2.8 | 19.8 | 5.9 | 2.1 | 29 |
| CIBJS20171014007 | 31.6 | 12.2 | 3.5 | 4.7 | 7.1 | 3.2 | 2.5 | 18.3 | 5.6 | 1.2 | 28 |
| CIBJS20171014008 | 32.0 | 12.9 | 3.9 | 4.9 | 7.4 | 2.7 | 2.5 | 19.7 | 5.7 | 1.6 | 28 |
| CIBJS20171014009 | 35.7 | 13.9 | 3.3 | 5.6 | 8.2 | 3.3 | 2.9 | 22.0 | 6.3 | 1.8 | 29 |
| CIBJS201710140010 | 33.5 | 12.6 | 4.0 | 5.0 | 7.2 | 3.1 | 2.7 | 21.5 | 5.5 | 1.6 | 28 |
