# Supplementary material for: A new species of the odorous frog genus Odorrana (Amphibia, Anura, Ranidae) from southwestern China
Source: PeerJ. 2018 Oct 4;6:e5695. doi: 10.7717/peerj.5695 (PMC6174872; doi:10.7717/peerj.5695)
Supplement: Supplemental Information 5 [file peerj-06-5695-s005.docx]

|  | 1 | 2 | 3 | 4 | 5 | 6 | 7 | 8 | 9 | 10 | 11 | 12 | 13 | 14 | 15 | 16 | 17 | 18 | 19 | 20 | 21 | 22 | 23 | 24 | 25 | 26 | 27 | 28 | 29 | 30 | 31 | 32 | 33 | 34 | 35 | 36 | 37 | 38 |
| --- | --- | --- | --- | --- | --- | --- | --- | --- | --- | --- | --- | --- | --- | --- | --- | --- | --- | --- | --- | --- | --- | --- | --- | --- | --- | --- | --- | --- | --- | --- | --- | --- | --- | --- | --- | --- | --- | --- |
| 1 *Odorrana kweichowensis* **sp. nov.** |  |  |  |  |  |  |  |  |  |  |  |  |  |  |  |  |  |  |  |  |  |  |  |  |  |  |  |  |  |  |  |  |  |  |  |  |  |  |
| 2 *Odorrana schmackeri* | **0.027** |  |  |  |  |  |  |  |  |  |  |  |  |  |  |  |  |  |  |  |  |  |  |  |  |  |  |  |  |  |  |  |  |  |  |  |  |  |
| 3 *Odorrana tiannanensis* | 0.053 | 0.051 |  |  |  |  |  |  |  |  |  |  |  |  |  |  |  |  |  |  |  |  |  |  |  |  |  |  |  |  |  |  |  |  |  |  |  |  |
| 4 *Odorrana bacboensis* | 0.046 | 0.046 | **0.009** |  |  |  |  |  |  |  |  |  |  |  |  |  |  |  |  |  |  |  |  |  |  |  |  |  |  |  |  |  |  |  |  |  |  |  |
| 5 *Odorrana hainanensis* | 0.053 | 0.065 | 0.030 | 0.046 |  |  |  |  |  |  |  |  |  |  |  |  |  |  |  |  |  |  |  |  |  |  |  |  |  |  |  |  |  |  |  |  |  |  |
| 6 *Odorrana fengkaiensis* | 0.053 | 0.067 | 0.028 | 0.046 | **0.014** |  |  |  |  |  |  |  |  |  |  |  |  |  |  |  |  |  |  |  |  |  |  |  |  |  |  |  |  |  |  |  |  |  |
| 7 *Odorrana nanjiangensis* | 0.042 | 0.051 | 0.039 | 0.033 | 0.045 | 0.047 |  |  |  |  |  |  |  |  |  |  |  |  |  |  |  |  |  |  |  |  |  |  |  |  |  |  |  |  |  |  |  |  |
| 8 *Odorrana hejiangensis* | 0.043 | 0.053 | 0.041 | 0.033 | 0.047 | 0.049 | **0.002** |  |  |  |  |  |  |  |  |  |  |  |  |  |  |  |  |  |  |  |  |  |  |  |  |  |  |  |  |  |  |  |
| 9 *Odorrana huanggangensis* | 0.055 | 0.062 | 0.063 | 0.039 | 0.067 | 0.065 | 0.051 | 0.053 |  |  |  |  |  |  |  |  |  |  |  |  |  |  |  |  |  |  |  |  |  |  |  |  |  |  |  |  |  |  |
| 10 *Odorrana tianmuii* | 0.051 | 0.061 | 0.067 | 0.046 | 0.067 | 0.065 | 0.051 | 0.050 | **0.014** |  |  |  |  |  |  |  |  |  |  |  |  |  |  |  |  |  |  |  |  |  |  |  |  |  |  |  |  |  |
| 11 *Odorrana narina* | 0.075 | 0.083 | 0.077 | 0.059 | 0.087 | 0.085 | 0.071 | 0.069 | 0.082 | 0.072 |  |  |  |  |  |  |  |  |  |  |  |  |  |  |  |  |  |  |  |  |  |  |  |  |  |  |  |  |
| 12 *Odorrana amamiensis* | 0.073 | 0.075 | 0.083 | 0.095 | 0.092 | 0.087 | 0.073 | 0.071 | 0.083 | 0.069 | 0.028 |  |  |  |  |  |  |  |  |  |  |  |  |  |  |  |  |  |  |  |  |  |  |  |  |  |  |  |
| 13 *Odorrana supranarina* | 0.079 | 0.081 | 0.089 | 0.092 | 0.099 | 0.089 | 0.077 | 0.075 | 0.087 | 0.077 | 0.063 | 0.061 |  |  |  |  |  |  |  |  |  |  |  |  |  |  |  |  |  |  |  |  |  |  |  |  |  |  |
| 14 *Odorrana swinhoana* | 0.085 | 0.093 | 0.103 | 0.092 | 0.114 | 0.108 | 0.091 | 0.089 | 0.092 | 0.087 | 0.083 | 0.084 | 0.067 |  |  |  |  |  |  |  |  |  |  |  |  |  |  |  |  |  |  |  |  |  |  |  |  |  |
| 15 *Odorrana utsunomiyaorum* | 0.089 | 0.083 | 0.095 | 0.092 | 0.103 | 0.101 | 0.085 | 0.083 | 0.098 | 0.089 | 0.094 | 0.093 | 0.071 | 0.102 |  |  |  |  |  |  |  |  |  |  |  |  |  |  |  |  |  |  |  |  |  |  |  |  |
| 16 *Odorrana nasuta* | 0.093 | 0.097 | 0.099 | 0.092 | 0.097 | 0.095 | 0.089 | 0.087 | 0.103 | 0.092 | 0.085 | 0.085 | 0.077 | 0.109 | 0.101 |  |  |  |  |  |  |  |  |  |  |  |  |  |  |  |  |  |  |  |  |  |  |  |
| 17 *Odorrana versabilis* | 0.088 | 0.093 | 0.095 | 0.078 | 0.103 | 0.101 | 0.087 | 0.085 | 0.103 | 0.092 | 0.080 | 0.078 | 0.069 | 0.095 | 0.095 | 0.028 |  |  |  |  |  |  |  |  |  |  |  |  |  |  |  |  |  |  |  |  |  |  |
| 18 *Odorrana exiliversabilis* | 0.077 | 0.091 | 0.087 | 0.076 | 0.090 | 0.085 | 0.078 | 0.076 | 0.087 | 0.078 | 0.054 | 0.044 | 0.082 | 0.089 | 0.103 | 0.099 | 0.088 |  |  |  |  |  |  |  |  |  |  |  |  |  |  |  |  |  |  |  |  |  |
| 19 *Odorrana nasica* | 0.091 | 0.089 | 0.093 | 0.078 | 0.099 | 0.095 | 0.082 | 0.080 | 0.085 | 0.082 | 0.063 | 0.056 | 0.063 | 0.079 | 0.097 | 0.099 | 0.096 | 0.077 |  |  |  |  |  |  |  |  |  |  |  |  |  |  |  |  |  |  |  |  |
| 20 *Odorrana tormota* | 0.096 | 0.093 | 0.108 | 0.095 | 0.118 | 0.106 | 0.099 | 0.097 | 0.088 | 0.087 | 0.084 | 0.075 | 0.075 | 0.089 | 0.119 | 0.101 | 0.111 | 0.082 | 0.045 |  |  |  |  |  |  |  |  |  |  |  |  |  |  |  |  |  |  |  |
| 21 *Odorrana leporipes* | 0.079 | 0.085 | 0.089 | 0.085 | 0.099 | 0.093 | 0.081 | 0.079 | 0.091 | 0.081 | 0.079 | 0.073 | 0.024 | 0.065 | 0.077 | 0.077 | 0.069 | 0.082 | 0.081 | 0.091 |  |  |  |  |  |  |  |  |  |  |  |  |  |  |  |  |  |  |
| 22 *Odorrana graminea* | 0.067 | 0.071 | 0.079 | 0.072 | 0.091 | 0.085 | 0.067 | 0.065 | 0.083 | 0.073 | 0.067 | 0.059 | 0.018 | 0.057 | 0.067 | 0.069 | 0.058 | 0.075 | 0.071 | 0.080 | **0.014** |  |  |  |  |  |  |  |  |  |  |  |  |  |  |  |  |  |
| 23 *Odorrana chloronota* | 0.093 | 0.098 | 0.101 | 0.059 | 0.103 | 0.105 | 0.093 | 0.091 | 0.118 | 0.107 | 0.083 | 0.103 | 0.081 | 0.104 | 0.110 | 0.077 | 0.081 | 0.110 | 0.099 | 0.112 | 0.093 | 0.085 |  |  |  |  |  |  |  |  |  |  |  |  |  |  |  |  |
| 24 *Odorrana hosii* | 0.085 | 0.088 | 0.091 | 0.078 | 0.097 | 0.095 | 0.079 | 0.077 | 0.097 | 0.085 | 0.073 | 0.068 | 0.069 | 0.096 | 0.088 | 0.032 | 0.028 | 0.080 | 0.085 | 0.103 | 0.073 | 0.063 | 0.080 |  |  |  |  |  |  |  |  |  |  |  |  |  |  |  |
| 25 *Odorrana morafkai* | 0.089 | 0.093 | 0.101 | 0.085 | 0.113 | 0.109 | 0.089 | 0.087 | 0.095 | 0.092 | 0.087 | 0.089 | 0.075 | 0.089 | 0.100 | 0.073 | 0.063 | 0.096 | 0.081 | 0.105 | 0.081 | 0.069 | 0.100 | 0.069 |  |  |  |  |  |  |  |  |  |  |  |  |  |  |
| 26 *Odorrana banaorum* | 0.089 | 0.093 | 0.101 | 0.092 | 0.113 | 0.109 | 0.089 | 0.087 | 0.095 | 0.092 | 0.085 | 0.082 | 0.069 | 0.081 | 0.098 | 0.075 | 0.065 | 0.089 | 0.075 | 0.098 | 0.075 | 0.063 | 0.106 | 0.075 | **0.014** |  |  |  |  |  |  |  |  |  |  |  |  |  |
| 27 *Odorrana ishikawae* | 0.069 | 0.069 | 0.067 | 0.052 | 0.079 | 0.075 | 0.063 | 0.061 | 0.093 | 0.081 | 0.077 | 0.082 | 0.077 | 0.091 | 0.081 | 0.083 | 0.085 | 0.092 | 0.091 | 0.101 | 0.077 | 0.065 | 0.086 | 0.079 | 0.091 | 0.093 |  |  |  |  |  |  |  |  |  |  |  |  |
| 28 *Odorrana grahami* | 0.079 | 0.081 | 0.085 | 0.059 | 0.096 | 0.096 | 0.067 | 0.065 | 0.084 | 0.073 | 0.073 | 0.075 | 0.085 | 0.093 | 0.091 | 0.083 | 0.087 | 0.094 | 0.087 | 0.085 | 0.089 | 0.075 | 0.099 | 0.081 | 0.095 | 0.095 | 0.077 |  |  |  |  |  |  |  |  |  |  |  |
| 29 *Odorrana junlianensis* | 0.081 | 0.083 | 0.083 | 0.059 | 0.094 | 0.094 | 0.069 | 0.067 | 0.082 | 0.075 | 0.075 | 0.078 | 0.087 | 0.095 | 0.093 | 0.085 | 0.089 | 0.097 | 0.089 | 0.087 | 0.091 | 0.077 | 0.101 | 0.083 | 0.097 | 0.097 | 0.079 | **0.002** |  |  |  |  |  |  |  |  |  |  |
| 30 *Odorrana daorum* | 0.077 | 0.080 | 0.077 | 0.052 | 0.089 | 0.089 | 0.067 | 0.065 | 0.083 | 0.077 | 0.083 | 0.092 | 0.091 | 0.098 | 0.086 | 0.087 | 0.090 | 0.103 | 0.089 | 0.094 | 0.091 | 0.077 | 0.100 | 0.083 | 0.101 | 0.101 | 0.076 | **0.018** | **0.016** |  |  |  |  |  |  |  |  |  |
| 31 *Odorrana hmongorum* | 0.052 | 0.039 | 0.046 | 0.046 | 0.065 | 0.065 | 0.039 | 0.039 | 0.052 | 0.059 | 0.046 | 0.067 | 0.085 | 0.085 | 0.072 | 0.085 | 0.078 | 0.067 | 0.072 | 0.076 | 0.078 | 0.065 | 0.065 | 0.072 | 0.085 | 0.092 | 0.059 | **0.013** | **0.013** | **0.007** |  |  |  |  |  |  |  |  |
| 32 *Odorrana andersonii* | 0.079 | 0.083 | 0.083 | 0.065 | 0.095 | 0.095 | 0.065 | 0.063 | 0.089 | 0.081 | 0.075 | 0.078 | 0.083 | 0.097 | 0.093 | 0.083 | 0.087 | 0.097 | 0.089 | 0.094 | 0.083 | 0.069 | 0.101 | 0.085 | 0.093 | 0.093 | 0.075 | **0.014** | **0.012** | **0.022** | **0.020** |  |  |  |  |  |  |  |
| 33 *Odorrana jingdongensis* | 0.077 | 0.083 | 0.079 | 0.065 | 0.091 | 0.091 | 0.069 | 0.067 | 0.086 | 0.079 | 0.079 | 0.078 | 0.081 | 0.099 | 0.081 | 0.091 | 0.089 | 0.097 | 0.089 | 0.099 | 0.085 | 0.071 | 0.107 | 0.087 | 0.097 | 0.093 | 0.081 | **0.018** | **0.016** | **0.026** | **0.020** | **0.018** |  |  |  |  |  |  |
| 34 *Odorrana kuangwuensis* | 0.085 | 0.091 | 0.091 | 0.078 | 0.102 | 0.102 | 0.073 | 0.071 | 0.092 | 0.081 | 0.075 | 0.083 | 0.081 | 0.099 | 0.087 | 0.093 | 0.087 | 0.097 | 0.089 | 0.099 | 0.089 | 0.075 | 0.107 | 0.085 | 0.093 | 0.093 | 0.081 | **0.022** | **0.024** | 0.033 | 0.033 | **0.026** | **0.020** |  |  |  |  |  |
| 35 *Odorrana margaretae* | 0.087 | 0.093 | 0.096 | 0.078 | 0.108 | 0.108 | 0.083 | 0.081 | 0.100 | 0.089 | 0.081 | 0.083 | 0.087 | 0.108 | 0.097 | 0.095 | 0.089 | 0.106 | 0.093 | 0.104 | 0.095 | 0.081 | 0.101 | 0.087 | 0.095 | 0.099 | 0.081 | 0.028 | 0.030 | 0.039 | 0.033 | 0.028 | **0.022** | **0.022** |  |  |  |  |
| 36 *Odorrana wuchuanensis* | 0.071 | 0.075 | 0.073 | 0.039 | 0.085 | 0.085 | 0.057 | 0.055 | 0.075 | 0.063 | 0.067 | 0.069 | 0.075 | 0.079 | 0.089 | 0.082 | 0.080 | 0.083 | 0.082 | 0.085 | 0.075 | 0.061 | 0.097 | 0.069 | 0.089 | 0.089 | 0.071 | **0.026** | 0.028 | 0.032 | 0.020 | 0.033 | 0.035 | 0.039 | 0.045 |  |  |  |
| 37 *Odorrana yizhangensis* | 0.093 | 0.097 | 0.096 | 0.059 | 0.100 | 0.104 | 0.078 | 0.076 | 0.100 | 0.089 | 0.084 | 0.090 | 0.093 | 0.097 | 0.103 | 0.104 | 0.095 | 0.102 | 0.100 | 0.106 | 0.097 | 0.086 | 0.109 | 0.097 | 0.095 | 0.097 | 0.081 | 0.066 | 0.068 | 0.068 | 0.026 | 0.076 | 0.062 | 0.066 | 0.064 | 0.059 |  |  |
| 38 *Odorrana lungshengensis* | 0.079 | 0.082 | 0.078 | 0.059 | 0.085 | 0.089 | 0.070 | 0.068 | 0.090 | 0.080 | 0.065 | 0.071 | 0.086 | 0.091 | 0.085 | 0.104 | 0.092 | 0.088 | 0.083 | 0.099 | 0.093 | 0.080 | 0.097 | 0.090 | 0.094 | 0.092 | 0.069 | 0.060 | 0.062 | 0.058 | 0.026 | 0.066 | 0.056 | 0.060 | 0.062 | 0.056 | 0.035 |  |
| 39 *Odorrana anlungensis* | 0.085 | 0.089 | 0.093 | 0.065 | 0.093 | 0.095 | 0.075 | 0.073 | 0.102 | 0.091 | 0.081 | 0.094 | 0.087 | 0.103 | 0.075 | 0.093 | 0.086 | 0.108 | 0.103 | 0.115 | 0.091 | 0.079 | 0.103 | 0.081 | 0.105 | 0.103 | 0.053 | 0.059 | 0.061 | 0.057 | 0.033 | 0.067 | 0.059 | 0.063 | 0.067 | 0.057 | 0.055 | 0.048 |
