# Supplementary material for: A new species of the odorous frog genus Odorrana (Amphibia, Anura, Ranidae) from southwestern China
Source: PeerJ. 2018 Oct 4;6:e5695. doi: 10.7717/peerj.5695 (PMC6174872; doi:10.7717/peerj.5695)
Supplement: Supplemental Information 6 — Unit: mm. Abbreviations for the morphometric characters refer to Methods section. [file peerj-06-5695-s006.docx]

|  | *Odorrana kweichowensis* sp. nov. | | | | |  | *O. schmackeri* | | | | |  | *O. huanggangensis* | | | | |
| --- | --- | --- | --- | --- | --- | --- | --- | --- | --- | --- | --- | --- | --- | --- | --- | --- | --- |
|  | Females (n = 16) | |  | Males (n = 9) | |  | Females (n = 4) | |  | Males (n = 15) | |  | Females (n = 10) | |  | Males (n = 13) | |
|  | Range | Mean ± SD |  | Range | Mean ± SD |  | Range | Mean ± SD |  | Range | Mean ± SD |  | Range | Mean ± SD |  | Range | Mean ± SD |
| SVL | 62.4–81.1 | 73.6 ± 5.5 |  | 36.2–43.3 | 41.0 ± 2.3 |  | 72.1–80.4 | 76.5 ± 3.7 |  | 37.9–44.8 | 42.0 ± 1.7 |  | 70.1–88.6 | 78.4 ± 5.5 |  | 38.0–46.7 | 42.2 ± 2.4 |
| HDL | 24.4–30.9 | 28.2 ± 2.0 |  | 14.1–17.3 | 15.8 ± 1.2 |  | 27.1–32.3 | 29.5 ± 2.3 |  | 15.4–18.6 | 17.3 ± 0.8 |  | 25.6–32.1 | 28.9 ± 2.0 |  | 14.2–18.0 | 16.5 ± 1.2 |
| HDW | 21.5–29.5 | 26.2 ± 2.0 |  | 125–15.7 | 14.5 ± 1.0 |  | 25.7–27.5 | 26.6 ± 0.8 |  | 13.9–16.3 | 15.2 ± 0.7 |  | 25.0–31.9 | 28.0 ± 2.0 |  | 12.7–16.0 | 15.0 ± 0.9 |
| SL | 9.8–12.6 | 11.5 ± 0.7 |  | 5.5–67.0 | 6.40 ± 0.47 |  | 11.2–12.6 | 11.7 ± 0.6 |  | 6.1–7.6 | 7.0 ± 0.4 |  | 10.4–13.7 | 12.2 ± 1.1 |  | 5.9–7.4 | 6.9 ± 0.5 |
| ED | 7.2–9.9 | 8.8 ± 0.8 |  | 4.8–6.2 | 5.6 ± 0.8 |  | 8.3–9.7 | 9.2 ± 0.6 |  | 3.6–6.9 | 6.0 ± 0.8 |  | 7.6–10.4 | 9.4 ± 0.9 |  | 4.4–7.3 | 6.0 ± 0.7 |
| IOD | 4.8–6.6 | 5.7 ± 0.6 |  | 2.6–3.5 | 3.0 ± 0.3 |  | 4.8–6.6 | 5.8 ± 0.89 |  | 2.9–4.1 | 3.5 ± 0.3 |  | 4.4–6.9 | 5.7 ± 0.8 |  | 2.7–3.9 | 3.22 ± 0.35 |
| IND | 6.7–9.8 | 8.4 ± 0.7 |  | 4.5–5.8 | 4.9 ± 0.4 |  | 8.2–8.7 | 8.5 ± 0.2 |  | 4.5–5.3 | 5.0 ± 0.3 |  | 8.2–9.8 | 8.9 ± 0.5 |  | 4.1–5.3 | 4.8 ± 0.4 |
| NED | 5.2–7.2 | 6.2 ± 0.6 |  | 3.1–4.0 | 3.6 ± 0.3 |  | 5.8–7.0 | 6.6 ± 0.5 |  | 3.2–4.2 | 3.8 ± 0.3 |  | 5.8–7.1 | 6.5 ± 0.4 |  | 3.0–4.6 | 3.7 ± 0.4 |
| NSD | 3.9–5.6 | 4.8 ± 0.4 |  | 1.8–3.0 | 2.7 ± 0.4 |  | 5.0–6.3 | 5.7 ± 0.7 |  | 2.1–3.5 | 3.0 ± 0.4 |  | 4.2–6.0 | 5.4 ± 0.5 |  | 2.4–3.9 | 3.1 ± 0.4 |
| IFE | 11.3–15.0 | 13.5 ± 0.9 |  | 7.2–8.9 | 8.1 ± 0.6 |  | 13.2–14.7 | 13.9 ± 0.8 |  | 7.2–8.8 | 8.3 ± 0.4 |  | 13.0–16.4 | 14.6 ± 1.1 |  | 7.3–9.0 | 8.2 ± 0.5 |
| IAE | 16.7–21.8 | 19.6 ± 1.7 |  | 10.2–13.6 | 12.1 ± 1.1 |  | 20.1–22.2 | 21.4 ± 1.0 |  | 11.8–13.0 | 13.0 ± 0.7 |  | 19.2–23.1 | 21.2 ± 1.3 |  | 10.6–14.6 | 13.1 ± 1.0 |
| TYD | 4.3–5.2 | 4.7 ± 0.3 |  | 2.9–4.5 | 3.9 ± 0.4 |  | 4.9–5.3 | 5.1 ± 0.2 |  | 3.3–5.2 | 4.2 ± 0.4 |  | 4.7–5.7 | 5.2 ± 0.3 |  | 3.2–4.4 | 3.9 ± 0.4 |
| LAL | 31.7–41.4 | 37.2 ± 2.7 |  | 19.0–24.3 | 21.1 ± 1.5 |  | 35.0–36.6 | 35.9 ± 0.6 |  | 19.2–22.4 | 20.6 ± 0.9 |  | 32.5–41.0 | 36.6 ± 2.6 |  | 18.5–23.1 | 20.6 ± 1.3 |
| LW | 5.2–7.8 | 6.2 ± 0.8 |  | 3.2–4.4 | 3.9 ± 0.4 |  | 5.8–7.2 | 6.4 ± 0.6 |  | 3.8–5.2 | 4.5 ± 0.5 |  | 5.2–8.5 | 6.7 ± 0.9 |  | 3.1–5.5 | 4.4 ± 0.7 |
| THL | 32.8–44.9 | 40.7 ± 3.3 |  | 19.6–25.2 | 22.8 ± 2.0 |  | 37.7–41.4 | 39.6 ± 1.9 |  | 20.7–24.6 | 22.2 ± 0.9 |  | 37.4–46.2 | 41.4 ± 2.6 |  | 19.6–24.4 | 22.5 ± 1.3 |
| TL | 39.3–49.8 | 45.9 ± 3.1 |  | 22.7–28.8 | 24.8 ± 1.9 |  | 43.0–46.2 | 44.8 ± 1.3 |  | 21.7–25.4 | 24.2 ± 1.0 |  | 41.8–53.1 | 46.6 ± 3.7 |  | 22.6–27.6 | 24.9 ± 1.5 |
| TW | 7.9–11.2 | 9.7 ± 1.1 |  | 4.4–5.6 | 5.1 ± 0.5 |  | 9.6–11.2 | 10.3 ± 0.7 |  | 4.8–6.3 | 5.6 ± 0.4 |  | 9.3–13.0 | 11.1 ± 1.2 |  | 4.7–6.2 | 5.5 ± 0.5 |
| TFL | 51.9–70.0 | 61.5 ± 4.7 |  | 30.1–38.7 | 34.3 ± 2.5 |  | 60.3–63.5 | 61.6 ± 1.4 |  | 30.6–36.7 | 33.7 ± 1.8 |  | 55.7–71.4 | 63.3 ± 4.6 |  | 29.3–37.7 | 33.1 ± 2.6 |
| FL | 35.2–48.1 | 43.4 ± 3.6 |  | 20.2–26.8 | 23.6 ± 2.0 |  | 42.0–42.8 | 42.4 ± 0.4 |  | 21.5–25.0 | 23.6 ± 1.0 |  | 38.4–48.3 | 44.2 ± 2.9 |  | 20.4–26.0 | 23.6 ± 1.7 |
| FDW | 2.3–3.5 | 2.8 ± 0.4 |  | 1.2–1.9 | 1.5 ± 0.2 |  | 2.5–3.2 | 2.8 ± 0.3 |  | 1.4–2.1 | 1.8 ± 0.3 |  | 2.7–3.4 | 3.0 ± 0.2 |  | 1.2–2.1 | 1.62 ± 0.3 |
| DPW | 2.0–3.1 | 2.5 ± 0.3 |  | 1.2–1.8 | 1.4 ± 0.2 |  | 2.3–2.9 | 2.5 ± 0.3 |  | 1.3–2.0 | 1.6 ± 0.2 |  | 2.1–3.3 | 2.8 ± 0.4 |  | 1.0–1.9 | 1.4 ± 0.3 |
