# Supplementary material for: A new species of the odorous frog genus Odorrana (Amphibia, Anura, Ranidae) from southwestern China
Source: PeerJ. 2018 Oct 4;6:e5695. doi: 10.7717/peerj.5695 (PMC6174872; doi:10.7717/peerj.5695)
Supplement: Supplemental Information 7 — Voucher information for each sequence refer to Table S2. [file peerj-06-5695-s007.zip › raw data/12S rRNA sequence in this study.docx]

12S rRNA sequence in this study (sequence ID refer to Table 1 ):

>CIBGYU20130917004

TCACACCTATAAGCGCCAGGGAACTACGAGCCATGCTTAAAACCCAAAGGATTTGACGGT

GTTCCACCCAGCTAGAGGAGCCTGTTCTATAATCGATGATCCCCGATACACCCGACCACC

CCTAGCTTATCAGTCTGTATACCTCCGTCGAAAGCTTACCATGTGAACGTC-T--ACAGT

AGGCCCAAGGACCCGCATTAGCGCGTCAATACGTCAGGTCAAGGTGCAACTTAAGGAGTG

GAAAGTGATTGGCTACAATTTCTAACCTAGAACAAACGAAAGACTGCGTGAAATCACAGT

CACGAAGGCGGATTTAGTAGTAAGAAGAAAATAGAGAGTTCTTTTTAACCCGGCCCTGGA

ACGCGTACACACCGCCCGT

>CIBGYU20130921001

TCACACCTATAAGCGCCAGGGAACTACGAGCCATGCTTAAAACCCAAAGGATTTGACGGT

GTTCCACCCAGCTAGAGGAGCCTGTTCTATAATCGATGATCCCCGATACACCCGACCACC

CCTAGCTTATCAGTCTGTATACCTCCGTCGAAAGCTTACCATGTGAACGTC-T--ACAGT

AGGCCCAAGGACCCGCATTAGCGCGTCAATACGTCAGGTCAAGGTGCAACTTAAGGAGTG

GAAAGTGATTGGCTACAATTTCTAACCTAGAACAAACGAAAGACTGCGTGAAATCACAGT

CACGAAGGCGGATTTAGTAGTAAGAAGAAAATAGAGAGTTCTTTTTAACCCGGCCCTGGA

ACGCGTACACACCGCCCGT

>CIBGYU20130917005

TCACACCTATAAGCGCCAGGGAACTACGAGCCATGCTTAAAACCCAAAGGATTTGACGGT

GTTCCACCCAGCTAGAGGAGCCTGTTCTATAATCGATGATCCCCGATACACCCGACCACC

CCTAGCTTATCAGTCTGTATACCTCCGTCGAAAGCTTACCATGTGAACGTC-T--ACAGT

AGGCCCAAGGACCCGCATTAGCGCGTCAACACGTCAGGTCAAGGTGCAACTTAAGGAGTG

GAAAGTGATTGGCTACAATTTCTAACCTAGAACAAACGAAAGACTGCGTGAAATCACAGT

CACGAAGGCGGATTTAGTAGTAAAAAGAAAATAGAGAGTCCTTTTTAACCCGGCCCTGGA

ACGCGTACACACCGCCCGT

>CIBjs20150803008

TCACACCTATAAGCGCCAGGGAACTACGAGCCATGCTTAAAACCCAAAGGATTTGACGGT

GTTCCACCCAGCTAGAGGAGCCTGTTCTATAATCGATGATCCCCGATACACCCGACCACC

CCTAGCTTATCAGTCTGTATACCTCCGTCGAAAGCTTACCATGTGAACGTC-T--ACAGT

AGGCCCAAGGACCCGCATTAGCGCGTCAATACGTCAGGTCAAGGTGCAACTTAAGGAGTG

GAAAGTGATTGGCTACAATTTCTAACCTAGAACAAACGAAAGACTGCGTGAAATCACAGT

CACGAAGGCGGATTTAGTAGTAAGAAGAAAATAGAGAGTTCTTTTTAACCCGGCCCTGGA

ACGCGTACACACCGCCCGT

>CIBjs20171014001

TCACACCTATAAGCGCCAGGGAACTACGAGCCATGCTTAAAACCCAAAGGATTTGACGGT

GTTCCACCCAGCTAGAGGAGCCTGTTCTATAATCGATGATCCCCGATACACCCGACCACC

CCTAGCTTATCAGTCTGTATACCTCCGTCGAAAGCTTACCATGTGAACGTC-T--ACAGT

AGGCCCAAGGACCCGCATTAGCGCGTCAACACGTCAGGTCAAGGTGCAACTTAAGGAGTG

GAAAGTGATTGGCTACAATTTCTAACCTAGAACAAACGAAAGACTGCGTGAAATCACAGT

CACGAAGGCGGATTTAGTAGTAAAAAGAAAATAGAGAGTTCTTTTTAACCCGGCCCTGGA

ACGCGTACACACCGCCCGT

>CIB20130531

TTACACCCATAAGCGCCAGGGAACTACGAGCCATGCTTAAAACCCAAAGGATTTGACGGT

GTTCCACCCAACTAGAGGAGCCTGTTCTATAATCGATGATCCCCGATACACCCGACCACT

CCTAGCTTATCAGTCTGTATACCTCCGTCGAAAGCTTACCATGTGAACGTC-T--ACAGT

AGGCCCAAGGACCCGCAT-AGCGCGTCAATACGTCAGGTCAAGGTGCAACTTAAGGAGTG

GGAAGTAATTGGCTACAATTTCTAACCTAGAACAAACGAAAGACTGCATGAAATCACAGT

CACGAAGGCGGATTTAGCAGTAAAAAGAAAATAGAGAGTTCTTTTTAACCCGGCCCTGGA

ACGCGTACACACCGCCCGT

>CIB20130532

TTACACCCATAAGCGCCAGGGAACTACGAGCCATGCTTAAAACCCAAAGGATTTGACGGT

GTTCCACCCAACTAGAGGAGCCTGTTCTATAATCGATGATCCCCGATACACCCGACCACT

CCTAGCTTATCAGTCTGTATACCTCCGTCGAAAGCTTACCATGTGAACGTC-T--ACAGT

AGGCCCAAGGACCCGCAT-AGCGCGTCAATACGTCAGGTCAAGGTGCAACTTAAGGAGTG

GGAAGTAATTGGCTACAATTTCTAACCTAGAACAAACGAAAGACTGCATGAAATCACAGT

CACGAAGGCGGATTTAGCAGTAAAAAGAAAATAGAGAGTTCTTTTTAACCCGGCCCTGGA

ACGCGTACACACCGCCCGT

>CIB20130533

TTACACCCATAAGCGCCAGGGAACTACGAGCCATGCTTAAAACCCAAAGGATTTGACGGT

GTTCCACCCAACTAGAGGAGCCTGTTCTATAATCGATGATCCCCGATACACCCGACCACT

CCTAGCTTATCAGTCTGTATACCTCCGTCGAAAGCTTACCATGTGAACGTC-T--ACAGT

AGGCCCAAGGACCCGCAT-AGCGCGTCAATACGTCAGGTCAAGGTGCAACTTAAGGAGTG

GGAAGTAATTGGCTACAATTTCTAACCTAGAACAAACGAAAGACTGCATGAAATCACAGT

CACGAAGGCGGATTTAGCAGTAAAAAGAAAATAGAGAGTTCTTTTTAACCCGGCCCTGGA

ACGCGTACACACCGCCCGT

>CIBLC2010092

TTACACCCATAAGCGCCAGGGAACTACGAGCCATGCTTAAAACCCAAAGGATTTGACGGT

GTTCCACCCAACTAGAGGAGCCTGTTCTATAATCGATGATCCCCGATACACCCGACCACT

CCTAGCTTATCAGTCTGTATACCTCCGTCGAAAGCTTACCATGTGAACGTC-T--ACAGT

AGGCCCAAGGACCCGCAT-AGCGCGTCAATACGTCAGGTCAAGGTGCAACTTAAGGAGTG

GGAAGTAATTGGCTACAATTTCTAACCTAGAACAAACGAAAGACTGCATGAAATCACAGT

CACGAAGGCGGATTTAGCAGTAAAAAGAAAATAGAGAGTTCTTTTTAACCCGGCCCTGGA

ACGCGTACACACCGCCCGT

>CIBLC2010097

TTACACCCATAAGCGCCAGGGAACTACGAGCCATGCTTAAAACCCAAAGGATTTGACGGT

GTTCCACCCAACTAGAGGAGCCTGTTCTATAATCGATGATCCCCGATACACCCGACCACT

CCTAGCTTATCAGTCTGTATACCTCCGTCGAAAGCTTACCATGTGAACGTC-T--ACAGT

AGGCCCAAGGACCCGCAT-AGCGCGTCAATACGTCAGGTCAAGGTGCAACTTAAGGAGTG

GGAAGTAATTGGCTACAATTTCTAACCTAGAACAAACGAAAGACTGCATGAAATCACAGT

CACGAAGGCGGATTTAGCAGTAAAAAGAAAATAGAGAGTTCTTTTTAACCCGGCCCTGGA

ACGCGTACACACCGCCCGT

>CIBHN201108149

CTACACCTATAAGCGCCAGGGAATTACGAGCCATGCTTAAAACCCAAAGGATTTGACGGT

GTCCCACCCCCCTAGAGGAGCCTGTTCTATAATCGATGATCCCCGCTACACCCGACCACT

TCTTGCTTATCAGTCTGTATACCTCCGTCGAAAGCTTACCATGTGAACGCCACCCACAGT

AGGTTCAATGACCCCC--------GTCAATACGTCAGGTCAAGGTGCAGCTTAAGAAGTG

GGAAGTGATGGGCTACAATTTCTAATCTAGAACAAACGAAAGACTATGTGAAACCCTAGT

CACGAAGGCGGATTTAGCAGTAAAAAGAAAATAGAGTGTTCTTTTTAATCTGGCTCTGGG

ACGCGTACACACCGCCCGT

>CIBFJS20150501004

CTACACCTATAAGCGCCAGGGAATTACGAGCTATGCTTAAAACCCAAAGGATTTGACGGT

GTCCCACCCCCCTAGAGGAGCCTGTTCTATAATCGATGATCCCCGCTACACCCGACCACT

TCTTGCTTATCAGTCTGTATACCTCCGTCGAAAGCTTACCATGTGAACGCCACCCACAGT

AGGTTCAATGACCCCC--------GTCAATACGTCAGGTCAAGGTGCAGCTTAAGAAGTG

GGAAGTGATGGGCTACAATTTCTAATCTAGAACAAACGAAAGACTATGTGAAACCCTAGT

CACGAAGGCGGATTTAGCAGTAAAAAGAAAATAGAGTGTTCTTTTTAATCTGGCTCTGGG

ACGCGTACACACCGCCCGT

>CIBFJS20150501006

CTACACCTATAAGCGCCAGGGAATTACGAGCTATGCTTAAAACCCAAAGGATTTGACGGT

GTCCCACCCCCCTAGAGGAGCCTGTTCTATAATCGATGATCCCCGCTACACCCGACCACT

TCTTGCTTATCAGTCTGTATACCTCCGTCGAAAGCTTACCATGTGAACGCCACCCACAGT

AGGTTCAATGACCCCC--------GTCAATACGTCAGGTCAAGGTGCAGCTTAAGAAGTG

GGAAGTGATGGGCTACAATTTCTAATCTAGAACAAACGAAAGACTATGTGAAACCCTAGT

CACGAAGGCGGATTTAGCAGTAAAAAGAAAATAGAGTGTTCTTTTTAATCTGGCTCTGGG

ACGCGTACACACCGCCCGT

>CIBLS20140616004

CCACACCTATAAGCGCCAGGGAATTACGAGCCATGCTTAAAACCCAAAGGATTTGACGGT

GTCCCACCCCACTAGAGGAGCCTGTTCTATAATCGATAATCCCCGCTACACCCGACCATT

TCTTGCTTATCAGTCTGTATACCTCCGTCGAAAGCTTACCGTGTGAACGCC-CCCACAGT

AGGTTTAATGACCTCC--------GTCAATACGTCAGGTCAAGGTGCAGCTTAAGAAATG

GGAAGTAATGGGCTACAATTTCTAACCTAGAACAAACGAAAGACTATGTGAAACCCTAGT

CACGAAGGCGGATTTAGCAGTAAGAAGAAAATAGAGTGT---------------------

-------------------

>CIBLS20140616006

CCACACCTATAAGCGCCAGGGAATTACGAGCCATGCTTAAAACCCAAAGGATTTGACGGT

GTCCCACCCCACTAGAGGAGCCTGTTCTATAATCGATAATCCCCGCTACACCCGACCATT

TCTTGCTTATCAGTCTGTATACCTCCGTCGAAAGCTTACCGTGTGAACGCC-CCCACAGT

AGGTTTAATGACCTCC--------GTCAATACGTCAGGTCAAGGTGCAGCTTAAGAAATG

GGAAGTAATGGGCTACAATTTCTAACCTAGAACAAACGAAAGACTATGTGAAACCCTAGT

CACGAAGGCGGATTTAGCAGTAAGAAGAAAATAGAGTGTC--------------------

-------------------

>CIBLS20140818005

TCACACCAATAAGCGCCAGGGAACTACGAGCAATGCTTAAAACCCAAAGGATTTGACGGT

GTCCCACCCCCCTAGAGGAGCCTGTTCTATAATCGATGATCCCCGATATACCCCACCACT

CCTTGCTCATCAGTCTGTATACCTCCGTCGAAAGCTTACCGTATGAACGTC-T--ACAGT

AGGTTCAAGGACTCGCAC-AGCGAGTCAATACGTCAGGTCAAGGTGCAACTTAAGGAGTG

GAAAGTAATTGGCTACAATTTCTAATTTAGAACAAACGAAAGACTATGTGAAACCATAGT

AACGAAGGCGGATTTAGTAGTAAAAAGAAAATAGAGAGTTCTTTTTAACCCGGCTCTGGG

ACGCGTACACACCGCCCGT

>CIBGD201108030

TCACACCAATAAGCGCCAGGGAACTACGAGCAATGCTTAAAACCCAAAGGATTTGACGGT

GTCCCACCCCCCTAGAGGAGCCTGTTCTATAATCGATGATCCCCGATATACCCCACCACT

CCTTGCTCATCAGTCTGTATACCTCCGTCGAAAGCTTACCGTATGAACGTC-T--ACAGT

AGGTTCAAGGACTCGCAC-AGCGAGTCAATACGTCAGGTCAAGGTGCAACTTAAGGAGTG

GAAAGTAATTGGCTACAATTTCTAATTTAGAACAAACGAAAGACTATGTGAAACCATAGT

AACGAAGGCGGATTTAGTAGTAAAAAGAAAATAGAGAGTTCTTTTTAACCCGGCTCTGGG

ACGCGTACACACCGCCCGT

>CIBFJS20150502002

TCACACCAATAAGCGCCAGGGAACTACGAGCAATGCTTAAAACCCAAAGGATTTGACGGT

GTCCCACCCCCCTAGAGGAGCCTGTTCTATAATCGATGATCCCCGATATACCCCACCACT

CCTTGCTCATCAGTCTGTATACCTCCGTCGAAAGCTTACCGTATGAACGTC-T--ACAGT

AGGTTCAAGGACTCGCAC-AGCGAGTCAATACGTCAGGTCAAGGTGCAACTTAAGGAGTG

GAAAGTAATTGGCTACAATTTCTAATTTAGAACAAACGAAAGACTATGTGAAACCATAGT

AACGAAGGCGGATTTAGTAGTAAAAAGAAAATAGAGAGTTCTTTTTAACCCGGCTCTGGG

ACGCGTACACACCGCCCGT
