# Supplementary material for: A new species of the odorous frog genus Odorrana (Amphibia, Anura, Ranidae) from southwestern China
Source: PeerJ. 2018 Oct 4;6:e5695. doi: 10.7717/peerj.5695 (PMC6174872; doi:10.7717/peerj.5695)
Supplement: Supplemental Information 7 — Voucher information for each sequence refer to Table S2. [file peerj-06-5695-s007.zip › raw data/16S rRNA sequence in this study.docx]

16S rRNA sequence in this study (sequence ID refer to Table 1 ):

>CIBGYU20130917004

--ATTTAACGGCCGCGGTATACTAACCGTGCGAAGGTAGCATAATCACTTGTTCTTTAAA

TGGGGACTCGTATCAACGGCATCACGAGGGTTATACTGTCTCCTCTCTCTAATCAGTGAA

ACTGATCCTCCCGTGAAGAAGCGGGGATTTAATTATAAGACGAGAAGACCCCATGGAGCT

TTAAACCCGATATACACCTCA-CACTCAAACACCAACTTAACCTTAAAGGCCTGTACATC

GGTTTTAGGCTGGGGGGGCCACGGAGTAAAACTAAACCTCCATAACAAACGGACTAACAC

CCTAATCCACGAT-TTATAAATCTAAGAATCACCAAAATGATGTTTAATGACCCGATAAT

TCGATCAATGAACCAAGTTACCCTGGGGATAACAGCGCAATCCACTTCAAGAGCCCTTAT

CGACAAGTGGGTTTACGACCTCGATGTTGGATCAGGGTATCCTAGTGGTGCAACCGCTAC

TAAT-GGTTCGCTTGTTCAGCGATTAAAACCCTACGTGA

>CIBGYU20130921001

--ATTTAACGGCCGCGGTATACTAACCGTGCGAAGGTAGCATAATCACTTGTTCTTTAAA

TGGGGACTCGTATCAACGGCATCACGAGGGTTATACTGTCTCCTCTCTCTAATCAGTGAA

ACTGATCCTCCCGTGAAGAAGCGGGGATTTAATTATAAGACGAGAAGACCCCATGGAGCT

TTAAACCCGATATACACCTCA-CACTCAAACACCAACTTAACCTTAAAGGCCTGTACATC

GGTTTTAGGCTGGGGGGGCCACGGAGTAAAACTAAACCTCCATAACAAACGGACTAACAC

CCTAATCCACGAT-TTATAAATCTAAGAATCACCAAAATGATGTTTAATGACCCGATAAT

TCGATCAATGAACCAAGTTACCCTGGGGATAACAGCGCAATCCACTTCAAGAGCCCTTAT

CGACAAGTGGGTTTACGACCTCGATGTTGGATCAGGGTATCCTAGTGGTGCAACCGCTAC

TAAT-GGTTCGCTTGTTCAGCGATTAAAACCCTACGTGA

>CIBGYU20130917005

--ATTTAACGGCCGCGGTATACTAACCGTGCGAAGGTAGCATAATCACTTGTTCTTTAAA

TGGGGACTCGTATCAACGGCATCACGAGGGTTATACTGTCTCCTCTCTCTAATCAGTGAA

ACTGATCCTCCCGTGAAGAAGCGGGGATTTAATTATAAGACGAGAAGACCCCATGGAGCT

TTAAACCCGATATACACCTCA-CACTCAAACACCAACTTAACCTTAAAGGCCTGTACATC

GGTTTTAGGCTGGGGGGGCCACGGAGTAAAACTAAACCTCCATAACAAACGGACTAACAC

CCTAATCCACGAT-TTATAAATCTAAGAATCACCAAAATGATGTTTAATGACCCGATAAT

TCGATCAATGAACCAAGTTACCCTGGGGATAACAGCGCAATCCACTTCAAGAGCCCTTAT

CGACAAGTGGGTTTACGACCTCGATGTTGGATCAGGGTATCCTAGTGGTGCAACCGCTAC

TAAT-GGTTCGCTTGTTCAGCGATTAAAACCCTACGTGA

>CIBjs20150803008

--ATTTAACGGCCGCGGTATACTAACCGTGCGAAGGTAGCATAATCACTTGTTCTTTAAA

TGGGGACTCGTATCAACGGCATCACGAGGGTTATACTGTCTCCTCTCTCTAATCAGTGAA

ACTGATCCTCCCGTGAAGAAGCGGGGATTTAATTATAAGACGAGAAGACCCCATGGAGCT

TTAAACCCGATATACACCTCA-CACTCAAACACCAACTTAACCTTAAAGGCCTGTACATC

GGTTTTAGGCTGGGGGGGCCACGGAGTAAAACTAAACCTCCATAACAAACGGACTAACAC

CCTAATCCACGAT-TTATAAATCTAAGAATCACCAAAATGATGTTTAATGACCCGATAAT

TCGATCAATGAACCAAGTTACCCTGGGGATAACAGCGCAATCCACTTCAAGAGCCCTTAT

CGACAAGTGGGTTTACGACCTCGATGTTGGATCAGGGTATCCTAGTGGTGCAACCGCTAC

TAAT-GGTTCGCTTGTTCAGCGATTAAAACCCTACGTGA

>CIBjs20171014001

--ATTTAACGGCCGCGGTATACTAACCGTGCGAAGGTAGCATAATCACTTGTTCTTTAAA

TGGGGACTCGTATCAACGGCATCACGAGGGTTATACTGTCTCCTCTCTCTAATCAGTGAA

ACTGATCCTCCCGTGAAGAAGCGGGGATTTAATTATAAGACGAGAAGACCCCATGGAGCT

TTAAACCCGATATACACCTCA-CACTCAAACACCAACTTAACCTTAAAGGCCTGTACATC

GGTTTTAGGCTGGGGGGGCCACGGAGTAAAACTAAACCTCCATAACAAACGGACTAACAC

CCTAATCCACGAT-TTATAAATCTAAGAATCACCAAAATGATGTTTAATGACCCGATAAT

TCGATCAATGAACCAAGTTACCCTGGGGATAACAGCGCAATCCACTTCAAGAGCCCTTAT

CGACAAGTGGGTTTACGACCTCGATGTTGGATCAGGGTATCCTAGTGGTGCAACCGCTAC

TAAT-GGTTCGCTTGTTCAGCGATTAAAACCCTACGTGA

>CIB20130531

--ATTTAACGGCCGCGGTATACTAACCGTGCGAAGGTAGCATAATCACTTGTTCTTTAAA

TAGGGACTCGTATCAACGGCATCACGAGGGTTATACTGTCTCCTCCCTCTAATCAGTGAA

ACTGATCCCCCCGTGAAGAAGCGGGGATTTAACTATAAGACGAGAAGACCCCATGGAGCT

TTAAACCCGATATACACCCAGGCACTCAAATACCAACTTAACCTAAGAGGCCTGTACATC

GGTTTTAGGCTGGGGGGGCCACGGAGTAAAACTAAACCTCCATAACAAACGGACTAATAA

CCTAATCCACGAT-TTATAAATCTAAGAATCACCAAAGTGATGTTTAATGACCCGATAAT

TCGATCAATGAACCAAGTTACCCTGGGGATAACAGCGCAATCCACTTCAAGAGCCCCTAT

CGACAAGTGGGTTTACGACCTCGATGTTGGATCAGGGTATCCTAGTGGTGCAACCGCTAC

TAAT-GGTTCGCTTGTTCAGCGATTAAAACCCTACGTGA

>CIB20130532

--ATTTAACGGCCGCGGTATACTAACCGTGCGAAGGTAGCATAATCACTTGTTCTTTAAA

TAGGGACTCGTATCAACGGCATCACGAGGGTTATACTGTCTCCTCCCTCTAATCAGTGAA

ACTGATCCCCCCGTGAAGAAGCGGGGATTTAACTATAAGACGAGAAGACCCCATGGAGCT

TTAAACCCGATATACACCCAGGCACTCAAATACCAACTTAACCTAAGAGGCCTGTACATC

GGTTTTAGGCTGGGGGGGCCACGGAGTAAAACTAAACCTCCATAACAAACGGACTAATAA

CCTAATCCACGAT-TTATAAATCTAAGAATCACCAAAGTGATGTTTAATGACCCGATAAT

TCGATCAATGAACCAAGTTACCCTGGGGATAACAGCGCAATCCACTTCAAGAGCCCCTAT

CGACAAGTGGGTTTACGACCTCGATGTTGGATCAGGGTATCCTAGTGGTGCAACCGCTAC

TAAT-GGTTCGCTTGTTCAGCGATTAAAACCCTACGTGA

>CIB20130533

--ATTTAACGGCCGCGGTATACTAACCGTGCGAAGGTAGCATAATCACTTGTTCTTTAAA

TAGGGACTCGTATCAACGGCATCACGAGGGTTATACTGTCTCCTCCCTCTAATCAGTGAA

ACTGATCCCCCCGTGAAGAAGCGGGGATTTAACTATAAGACGAGAAGACCCCATGGAGCT

TTAAACCCGATATACACCCAGGCACTCAAATACCAACTTAACCTAAGAGGCCTGTACATC

GGTTTTAGGCTGGGGGGGCCACGGAGTAAAACTAAACCTCCATAACAAACGGACTAATAA

CCTAATCCACGAT-TTATAAATCTAAGAATCACCAAAGTGATGTTTAATGACCCGATAAT

TCGATCAATGAACCAAGTTACCCTGGGGATAACAGCGCAATCCACTTCAAGAGCCCCTAT

CGACAAGTGGGTTTACGACCTCGATGTTGGATCAGGGTATCCTAGTGGTGCAACCGCTAC

TAATTGGTTCGCTTGTTCAGCGATTAAAACCCTACGTGA

>CIBLC2010092

--ATTTAACGGCCGCGGTATACTAACCGTGCGAAGGTAGCATAATCACTTGTTCTTTAAA

TAGGGACTCGTATCAACGGCATCACGAGGGTTATACTGTCTCCTCCCTCTAATCAGTGAA

ACTGATCCCCCCGTGAAGAAGCGGGGATTTAACTATAAGACGAGAAGACCCCATGGAGCT

TTAAACCCGATATACACCCAGGCACTCAAATACCAACTTAACCTAAGAGGCCTGTACATC

GGTTTTAGGCTGGGGGGGCCACGGAGTAAAACTAAACCTCCATAACAAACGGACTAATAA

CCTAATCCACGAT-TTATAAATCTAAGAATCACCAAAGTGATGTTTAATGACCCGATAAT

TCGATCAATGAACCAAGTTACCCTGGGGATAACAGCGCAATCCACTTCAAGAGCCCCTAT

CGACAAGTGGGTTTACGACCTCGATGTTGGATCAGGGTATCCTAGTGGTGCAACCGCTAC

TAAT-GGTTCGCTTGTTCAGCGATTAAAACCCTACGTGA

>CIBLC2010097

--ATTTAACGGCCGCGGTATACTAACCGTGCGAAGGTAGCATAATCACTTGTTCTTTAAA

TAGGGACTCGTATCAACGGCATCACGAGGGTTATACTGTCTCCTCCCTCTAATCAGTGAA

ACTGATCCCCCCGTGAAGAAGCGGGGATTTAACTATAAGACGAGAAGACCCCATGGAGCT

TTAAACCCGATATACACCCAGGCACTCAAATACCAACTTAACCTAAGAGGCCTGTACATC

GGTTTTAGGCTGGGGGGGCCACGGAGTAAAACTAAACCTCCATAACAAACGGACTAATAA

CCTAATCCACGAT-TTATAAATCTAAGAATCACCAAAGTGATGTTTAATGACCCGATAAT

TCGATCAATGAACCAAGTTACCCTGGGGATAACAGCGCAATCCACTTCAAGAGCCCCTAT

CGACAAGTGGGTTTACGACCTCGATGTTGGATCAGGGTATCCTAGTGGTGCAACCGCTAC

TAAT-GGTTCGCTTGTTCAGCGATTAAAACCCTACGTGA

>CIBHN201108149

--ATTCAACGGCCGCGGTACACTAACCGTGCGAAGGTAGCATAATCACTTGTTCTTTAAA

TAGGGACTAGTATCAACGGCATCACGAGGGCTATACTGTCTCCTCTCTCTAATCAGTGAA

ACTGATCCCCCCGTGAAGAAGCGGGGATTCAATTATAAGACGAGAAGACCCCATGGAGCT

TTAAACCCAACATATACCCCCTTACCTGAGCACCAACTTAACCTGAGGGACTTATATGCT

GGTTTTAGGCTGGGGGGGCCACGGAGTAAAACTAAACCTCCATAGCAAATGGGCTAACAC

CCTTATCCATGATTTTACAAATCTAAGAATTACCAAAATAATGTTTAACGACCCGATAAC

TCGATCAATGAACTAAGTTACCCTGGGGATAACAGCGCAATCTACTTCAAGAGCCCCTAT

CGACAAGTAGGCTTACGACCTCGATGTTGGATCAGGGTATCCCAGTGGTGCAACCGCTAC

TGAC-GGTTCGTTTGTTCAACGATTAAAACCCTACGTGA

>CIBFJS20150501004

--ATTCAACGGCCGCGGTACACTAACCGTGCGAAGGTAGCATAATCACTTGTTCTTTAAA

TAGGGACTAGTATCAACGGCATCACGAGGGCTATACTGTCTCCTCTCTCTAATCAGTGAA

ACTGATCCCCCCGTGAAGAAGCGGGGATTCAATTATAAGACGAGAAGACCCCATGGAGCT

TTAAACCCAACATATACCCCCTTACCTGAGCACCAACTTAACCTAAGAGACTTATATGCT

GGTTTTAGGCTGGGGGGGCCACGGAGTAAAACTAAACCTCCATAGCAAATGGGCTAACAC

CCTTATCCATGATTTTACAAATCTAAGAATTACCAAAATAATGTTTAACGACCCGATAAC

TCGATCAATGAACTAAGTTACCCTGGGGATAACAGCGCAATCTACTTCAAGAGCCCCTAT

CGACAAGTAGGCTTACGACCTCGATGTTGGATCAGGGTATCCCAGTGGTGCAACCGCTAC

TGAC-GGTTCGTTTGTTCAACGATTAAAACCCTACGTGA

>CIBFJS20150501006

--ATTCAACGGCCGCGGTACACTAACCGTGCGAAGGTAGCATAATCACTTGTTCTTTAAA

TAGGGACTAGTATCAACGGCATCACGAGGGCTATACTGTCTCCTCTCTCTAATCAGTGAA

ACTGATCCCCCCGTGAAGAAGCGGGGATTCAATTATAAGACGAGAAGACCCCATGGAGCT

TTAAACCCAACATATACCCCCTTACCTGAGCACCAACTTAACCTAAGAGACTTATATGCT

GGTTTTAGGCTGGGGGGGCCACGGAGTAAAACTAAACCTCCATAGCAAATGGGCTAACAC

CCTTATCCATGATTTTACAAATCTAAGAATTACCAAAATAATGTTTAACGACCCGATAAC

TCGATCAATGAACTAAGTTACCCTGGGGATAACAGCGCAATCTACTTCAAGAGCCCCTAT

CGACAAGTAGGCTTACGACCTCGATGTTGGATCAGGGTATCCCAGTGGTGCAACCGCTAC

TGAC-GGTTCGTTTGTTCAACGATTAAAACCCTACGTGA

>CIBLS20140616004

CCATTTAACGGCCGCGGTACACTAACCGTGCGAAGGTAGCATAATCACTTGTTCTTTAAA

TAGGGACTAGTATCAACGGCATCACGAGGGCTATACTGTCTCCTCTCTCTAATCAGTGAA

ACTGATCCCCCCGTGAAGAAGCGGGGATTTAATTATAAGACGAGAAGACCCCATGGAGCT

TTAAACCCAACATATACCCCTTCACCTGAGCACCAACTTAACCCAAGAGACTTATATGTT

GGTTTTAGGCTGGGGGGGCCACGGAGTAAAATTAAACCTCCATAACAAATGGGCTAGCAC

CCTTATCTATGATCTTACAAATCTAAGAATTACCAAAATAATGTTTAATGACCCGATAAC

TCGATCAATGAACCAAGTTACCCTGGGGATAACAGCGCAATCTACTTCAAGAGCCCCTAT

CGACAAGTGGGTTTACGACCTCGATGTTGGATCAGGGTATCCTAGTGGTGCAACCGCTAC

TGAT-GGTTCGTTTGTTCAACGATTAAAACCCTACGTGA

>CIBLS20140616006

--ATTTAACGGCCGCGGTACACTAACCGTGCGAAGGTAGCATAATCACTTGTTCTTTAAA

TAGGGACTAGTATCAACGGCATCACGAGGGCTATACTGTCTCCTCTCTCTAATCAGTGAA

ACTGATCCCCCCGTGAAGAAGCGGGGATTTAATTATAAGACGAGAAGACCCCATGGAGCT

TTAAACCCAACATATACCCCTTCACCTGAGCACCAACTTAACCCAAGAGACTTATATGTT

GGTTTTAGGCTGGGGGGGCCACGGAGTAAAATTAAACCTCCATAACAAATGGGCTAGCAC

CCTTATCTATGATCTTACAAATCTAAGAATTACCAAAATAATGTTTAATGACCCGATAAC

TCGATCAATGAACCAAGTTACCCTGGGGATAACAGCGCAATCTACTTCAAGAGCCCCTAT

CGACAAGTGGGTTTACGACCTCGATGTTGGATCAGGGTATCCTAGTGGTGCAACCGCTAC

TGAT-GGTTCGTTTGTTCAACGATTAAAACCCTACGTGA

>CIBLS20140818005

--ATTAAACGGCCGCGGTATACTAACCGTGCGAAGGTAGCATAATCACTTGTTCTTTAAA

TGGGGACTTGTATCAACGGCATCACGAGGGTTATACTGTCTCCTCTCTCCAATCAGTGAA

ACTGATCCCCCCGTGAAGAAGCGGGGATTTTACTATAAGACGAGAAGACCCCATGGAGCT

TTAAACTCGACATACACTAAGGTATTAAAACACCAATTTAACCCAAAAA-CCTGTACGTC

AGTTTTAGGCTGGGGGGGCCACGGAGTAAAACTAAACCTCCACAACAAATGGGCTAACAC

CCTAATCTAAGAT-TTATAAATCTAAGAATCACCAAAATGATGTTTAATGACCCGATAAT

TCGATCAATGAACCAAGTTACCCTGGGGATAACAGCGCAATCTACTTCAAGAGCCCTTAT

CGACAAGTGGGTTTACGACCTCGATGTTGGATCAGGGTATCCTAGTGGTGCAACCGCTAC

TAAC-GGTTCGCTTGTTCAGCGATTAAAACCCTACGTGA

>CIBGD201108030

-GATTAAACGGCCGCGGTATACTAACCGTGCGAAGGTAGCATAATCACTTGTTCTTTAAA

TGGGGACTTGTATCAACGGCATCACGAGGGTTATACTGTCTCCTCTCTCCAATCAGTGAA

ACTGATCCCCCCGTGAAGAAGCGGGGATTTTACTATAAGACGAGAAGACCCCATGGAGCT

TTAAACTCGACATACACTAAGGTATTAAAACACCAATTTAACCCAAAAA-CCTGTACGTC

AGTTTTAGGCTGGGGGGGCCACGGAGTAAAACTAAACCTCCACAACAAATGGGCTAACAC

CCTAATCTAAGAT-TTATAAATCTAAGAATCACCAAAATGATGTTTAATGACCCGATAAT

TCGATCAATGAACCAAGTTACCCTGGGGATAACAGCGCAATCTACTTCAAGAGCCCTTAT

CGACAAGTGGGTTTACGACCTCGATGTTGGATCAGGGTATCCTAGTGGTGCAACCGCTAC

TAAC-GGTTCGCTTGTTCAGCGATTAAAACCCTACGTGA

>CIBFJS20150502002

-CATTAAACGGCCGCGGTATACTAACCGTGCGAAGGTAGCATAATCACTTGTTCTTTAAA

TGGGGACTTGTATCAACGGCATCACGAGGGTTATACTGTCTCCTCTCTCCAATCAGTGAA

ACTGATCCCCCCGTGAAGAAGCGGGGATTTTACTATAAGACGAGAAGACCCCATGGAGCT

TTAAACTCGACATACACTAAGGTATTAAAACACCAATTTAACCCAAAAA-CCTGTACGTC

AGTTTTAGGCTGGGGGGGCCACGGAGTAAAACTAAACCTCCACAACAAATGGGCTAACAC

CCTAATCTAAGAT-TTATAAATCTAAGAATCACCAAAATGATGTTTAATGACCCGATAAT

TCGATCAATGAACCAAGTTACCCTGGGGATAACAGCGCAATCTACTTCAAGAGCCCTTAT

CGACAAGTGGGTTTACGACCTCGATGTTGGATCAGGGTATCCTAGTGGTGCAACCGCTAC

TAAC-GGTTCGCTTGTTCAGCGATTAAAACCCTACGTGA
