# Supplementary material for: A new species of the odorous frog genus Odorrana (Amphibia, Anura, Ranidae) from southwestern China
Source: PeerJ. 2018 Oct 4;6:e5695. doi: 10.7717/peerj.5695 (PMC6174872; doi:10.7717/peerj.5695)
Supplement: Supplemental Information 7 — Voucher information for each sequence refer to Table S2. [file peerj-06-5695-s007.zip › raw data/DOLK gene sequence in this study.docx]

DOLK gene sequence in this study (sequence ID refer to Table 1 ):

>CIBGYU20130917004

GGTGACTTTGGTGGCTTTGGTCCTGCTGGGCATTATTTTCTCAGCCTTGTTCTTTTTCAT

GGACTCCACCACCTGGACCTCCTCTTTATTTTTCTACATGATGACTGCGGTTTTAGGTCT

AGGAGTCTTTGTGCCCTGGCTGCAGTACCTCATCAAGAGACATCCGCTCTTCTGGCTGGT

GGAGTTTCTGGTTCAGAGCAAAACACGACTGTATCTCCTCCTCTTCTGGACATGCCTTGT

TCTTGTTGCTTGCGGGATTGTTCTATTTCAAAATTCGAAGAGATCCACAGACTCCAAAAA

GCCGCAAGTATCCACCATAACCCGCAAGTACTTTCACTTTCTTGCAGTGGTTGTCTACGT

TCCTGGAGTCATGTACGATCGTTCTTTGCTCTATGTGGCTGCAGTGGTATGTTTGGCTGT

TTTTGTGCTATTGGAATACATTCGCTTCTTCCACATTAAGCCACTTGGACAGACCTTACG

GACTCTTCTGACGCTGTTCCTGGATGAGCGGGATAGCGGTCCACTGATTCTGTCACACAT

TTACCTTCTGTTGGGGATGTCTCTACCGGTTTGGCTATTTCCAAGACTCTGCGCAACATC

TCTTTCTGGCCCCTCTACCTTGCTGCCCTATTGTGGCATTCTGGC

>CIBGYU20130921001

GGTGACTTTGGTGGCTTTGGTCCTGCTGGGCATTATTTTCTCAGCCTTGTTCTTTTTCAT

GGACTCCACCACCTGGACCTCCTCTTTATTTTTCTACATGATGACTGCGGTTTTAGGTCT

AGGAGTCTTTGTGCCCTGGCTGCAGTACCTCATCAAGAGACATCCGCTCTTCTGGCTGGT

GGAGTTTCTGGTTCAGAGCAAAACACGACTGTATCTCCTCCTCTTCTGGACATGCCTTGT

TCTTGTTGCTTGCGGGATTGTTCTATTTCAAAATTCGAAGAGATCCACAGACTCCAAAAA

GCCGCAAGTATCCACCATAACCCGCAAGTACTTTCACTTTCTTGCAGTGGTTGTCTACGT

TCCTGGAGTCATGTACGATCGTTCTTTGCTCTATGTGGCTGCAGTGGTATGTTTGGCTGT

TTTTGTGCTATTGGAATACATTCGCTTCTTCCACATTAAGCCACTTGGACAGACCTTACG

GACTCTTCTGACGCTGTTCCTGGATGAGCGGGATAGCGGTCCACTGATTCTGTCACACAT

TTACCTTCTGTTGGGGATGTCTCTACCGGTTTGGCTATTTCCAAGACTCTGCGCAACATC

TCTTTCTGGCCCCTCTACCTTGCTGCCCTATTGTGGCATTCTGGC

>CIBGYU20130917005

GGTGACTTTGGTGGCTTTGGTCCTGCTGGGCATTATTTTCTCAGCCTTGTTCTTTTTCAT

GGACTCCACCACCTGGACCTCCTCTTTATTTTTCTACATGATGACTGCGGTTTTAGGTCT

AGGAGTCTTTGTGCCCTGGCTGCAGTACCTCATCAAGAGACATCCGCTCTTCTGGCTGGT

GGAGTTTCTGGTTCAGAGCAAAACACGACTGTATCTCCTCCTCTTCTGGACATGCCTTGT

TCTTGTTGCTTGCGGGATTGTTCTATTTCAAAATTCGAAGAGATCCACAGACTCCAAAAA

GCCGCAAGTATCCACCATAACCCGCAAGTACTTTCACTTTCTTGCAGTGGTTGTCTACGT

TCCTGGAGTCATGTACGATCGTTCTTTGCTCTATGTGGCTGCAGTGGTATGTTTGGCTGT

TTTTGTGCTATTGGAATACATTCGCTTCTTCCACATTAAGCCACTTGGACAGACCTTACG

GACTCTTCTGACGCTGTTCCTGGATGAGCGGGATAGCGGTCCACTGATTCTGTCACACAT

TTACCTTCTGTTGGGGATGTCTCTACCGGTTTGGCTATTTCCAAGACTCTGCGCAACATC

TCTTTCTGGCCCCTCTACCTTGCTGCCCTATTGTGGCATTCTGGC

>CIBjs20150803008

GGTGACTTTGGTGGCTTTGGTCCTGCTGGGCATTATTTTCTCAGCCTTGTTCTTTTTCAT

GGACTCCACCACCTGGACCTCCTCTTTATTTTTCTACATGATGACTGCGGTTTTAGGTCT

AGGAGTCTTTGTGCCCTGGCTGCAGTACCTCATCAAGAGACATCCGCTCTTCTGGCTGGT

GGAGTTTCTGGTTCAGAGCAAAACACGACTGTATCTCCTCCTCTTCTGGACATGCCTTGT

TCTTGTTGCTTGCGGGATTGTTCTATTTCAAAATTCGAAGAGATCCACAGACTCCAAAAA

GCCGCAAGTATCCACCATAACCCGCAAGTACTTTCACTTTCTTGCAGTGGTTGTCTACGT

TCCTGGAGTCATGTACGATCGTTCTTTGCTCTATGTGGCTGCAGTGGTATGTTTGGCTGT

TTTTGTGCTATTGGAATACATTCGCTTCTTCCACATTAAGCCACTTGGACAGACCTTACG

GACTCTTCTGACGCTGTTCCTGGATGAGCGGGATAGCGGTCCACTGATTCTGTCACACAT

TTACCTTCTGTTGGGGATGTCTCTACCGGTTTGGCTATTTCCAAGACTCTGCGCAACATC

TCTTTCTGGCCCCTCTACCTTGCTGCCCTATTGTGGCATTCTGGC

>CIBjs20171014001

GGTGACTTTGGTGGCTTTGGTCCTGCTGGGCATTATTTTCTCAGCCTTGTTCTTTTTCAT

GGACTCCACCACCTGGACCTCCTCTTTATTTTTCTACATGATGACTGCGGTTTTAGGTCT

AGGAGTCTTTGTGCCCTGGCTGCAGTACCTCATCAAGAGACATCCGCTCTTCTGGCTGGT

GGAGTTTCTGGTTCAGAGCAAAACACGACTGTATCTCCTCCTCTTCTGGACATGCCTTGT

TCTTGTTGCTTGCGGGATTGTTCTATTTCAAAATTCGAAGAGATCCACAGACTCCAAAAA

GCCGCAAGTATCCACCATAACCCGCAAGTACTTTCACTTTCTTGCAGTGGTTGTCTACGT

TCCTGGAGTCATGTACGATCGTTCTTTGCTCTATGTGGCTGCAGTGGTATGTTTGGCTGT

TTTTGTGCTATTGGAATACATTCGCTTCTTCCACATTAAGCCACTTGGACAGACCTTACG

GACTCTTCTGACGCTGTTCCTGGATGAGCGGGATAGCGGTCCACTGATTCTGTCACACAT

TTACCTTCTGTTGGGGATGTCTCTACCGGTTTGGCTATTTCCAAGACTCTGCGCAACATC

TCTTTCTGGCCCCTCTACCTTGCTGCCCTATTGTGGCATTCTGGC

>CIB20130531

GGTTACTTTGGTGGCTTTGGTCCTGCTGGGCATTATTTTCTCAGCCTTGTTCTTTTTCAT

GGACTCCACCACCTGGACCTCCTCTTTATTTTTCTACATGATGACTGCGGTTTTAGGTCT

AGGAGTCTTTGTGCCCTGGCTGCAGTACCTCATCAAGAGACATCCGCTCTTCTGGCTGGT

GGAGTTTCTGGTTCAGAGCAAAACACGACTGTATCTCCTCCTCTTCTGGACATGCCTTGT

TCTTGTTGCTTGCGGGATTGTTCTATTTCAAAATTCGAAGAGATCTACAGACTCCAAAAA

GCCGCAAGTATCCACCATAACCCGCAAGTACTTTCACTTTCTTGCAGTGGTTGTCTACGT

TCCTGGAGTCATGTACGATCGTTCTTTGCTCTATGTGGCTGCAGTTGTATGTTTGGCTGT

TTTTGTGCTATTGGAATACGTTCGCTTCTTCCACATTAAGCCACTTGGACAGACCTTACG

GACTCTTCTGACGCTGTTCCTGGATGAGCGGGATAGCGGTCCACTGATTCTGTCACACAT

TTACCTTCTGTTGGGGATGTCTCTACCGGTTTGGCTATTTCCAAGACTCTGCGCAACATC

TCTTTCTGGCCCCTCTACCTTGCTGCCCTATTGTGGCATTCTGGC

>CIB20130532

GGTTACTTTGGTGGCTTTGGTCCTGCTGGGCATTATTTTCTCAGCCTTGTTCTTTTTCAT

GGACTCCACCACCTGGACCTCCTCTTTATTTTTCTACATGATGACTGCGGTTTTAGGTCT

AGGAGTCTTTGTGCCCTGGCTGCAGTACCTCATCAAGAGACATCCGCTCTTCTGGCTGGT

GGAGTTTCTGGTTCAGAGCAAAACACGACTGTATCTCCTCCTCTTCTGGACATGCCTTGT

TCTTGTTGCTTGCGGGATTGTTCTATTTCAAAATTCGAAGAGATCTACAGACTCCAAAAA

GCCGCAAGTATCCACCATAACCCGCAAGTACTTTCACTTTCTTGCAGTGGTTGTCTACGT

TCCTGGAGTCATGTACGATCGTTCTTTGCTCTATGTGGCTGCAGTTGTATGTTTGGCTGT

TTTTGTGCTATTGGAATACGTTCGCTTCTTCCACATTAAGCCACTTGGACAGACCTTACG

GACTCTTCTGACGCTGTTCCTGGATGAGCGGGATAGCGGTCCACTGATTCTGTCACACAT

TTACCTTCTGTTGGGGATGTCTCTACCGGTTTGGCTATTTCCAAGACTCTGCGCAACATC

TCTTTCTGGCCCCTCTACCTTGCTGCCCTATTGTGGCATTCTGGC

>CIB20130533

GGTTACTTTGGTGGCTTTGGTCCTGCTGGGCATTATTTTCTCAGCCTTGTTCTTTTTCAT

GGACTCCACCACCTGGACCTCCTCTTTATTTTTCTACATGATGACTGCGGTTTTAGGTCT

AGGAGTCTTTGTGCCCTGGCTGCAGTACCTCATCAAGAGACATCCGCTCTTCTGGCTGGT

GGAGTTTCTGGTTCAGAGCAAAACACGACTGTATCTCCTCCTCTTCTGGACATGCCTTGT

TCTTGTTGCTTGCGGGATTGTTCTATTTCAAAATTCGAAGAGATCTACAGACTCCAAAAA

GCCGCAAGTATCCACCATAACCCGCAAGTACTTTCACTTTCTTGCAGTGGTTGTCTACGT

TCCTGGAGTCATGTACGATCGTTCTTTGCTCTATGTGGCTGCAGTTGTATGTTTGGCTGT

TTTTGTGCTATTGGAATACGTTCGCTTCTTCCACATTAAGCCACTTGGACAGACCTTACG

GACTCTTCTGACGCTGTTCCTGGATGAGCGGGATAGCGGTCCACTGATTCTGTCACACAT

TTACCTTCTGTTGGGGATGTCTCTACCGGTTTGGCTATTTCCAAGACTCTGCGCAACATC

TCTTTCTGGCCCCTCTACCTTGCTGCCCTATTGTGGCATTCTGGC

>CIBLC2010092

GGTTACTTTGGTGGCTTTGGTCCTGCTGGGCATTATTTTCTCAGCCTTGTTCTTTTTCAT

GGACTCCACCACCTGGACCTCCTCTTTATTTTTCTACATGATGACTGCGGTTTTAGGTCT

AGGAGTCTTTGTGCCCTGGCTGCAGTACCTCATCAAGAGACATCCGCTCTTCTGGCTGGT

GGAGTTTCTGGTTCAGAGCAAAACACGACTGTATCTCCTCCTCTTCTGGACATGCCTTGT

TCTTGTTGCTTGCGGGATTGTTCTATTTCAAAATTCGAAGAGATCTACAGACTCCAAAAA

GCCGCAAGTATCCACCATAACCCGCAAGTACTTTCACTTTCTTGCAGTGGTTGTCTACGT

TCCTGGAGTCATGTACGATCGTTCTTTGCTCTATGTGGCTGCAGTTGTATGTTTGGCTGT

TTTTGTGCTATTGGAATACGTTCGCTTCTTCCACATTAAGCCACTTGGACAGACCTTACG

GACTCTTCTGACGCTGTTCCTGGATGAGCGGGATAGCGGTCCACTGATTCTGTCACACAT

TTACCTTCTGTTGGGGATGTCTCTACCGGTTTGGCTATTTCCAAGACTCTGCGCAACATC

TCTTTCTGGCCCCTCTACCTTGCTGCCCTATTGTGGCATTCTGGC

>CIBLC2010097

GGTTACTTTGGTGGCTTTGGTCCTGCTGGGCATTATTTTCTCAGCCTTGTTCTTTTTCAT

GGACTCCACCACCTGGACCTCCTCTTTATTTTTCTACATGATGACTGCGGTTTTAGGTCT

AGGAGTCTTTGTGCCCTGGCTGCAGTACCTCATCAAGAGACATCCGCTCTTCTGGCTGGT

GGAGTTTCTGGTTCAGAGCAAAACACGACTGTATCTCCTCCTCTTCTGGACATGCCTTGT

TCTTGTTGCTTGCGGGATTGTTCTATTTCAAAATTCGAAGAGATCTACAGACTCCAAAAA

GCCGCAAGTATCCACCATAACCCGCAAGTACTTTCACTTTCTTGCAGTGGTTGTCTACGT

TCCTGGAGTCATGTACGATCGTTCTTTGCTCTATGTGGCTGCAGTTGTATGTTTGGCTGT

TTTTGTGCTATTGGAATACGTTCGCTTCTTCCACATTAAGCCACTTGGACAGACCTTACG

GACTCTTCTGACGCTGTTCCTGGATGAGCGGGATAGCGGTCCACTGATTCTGTCACACAT

TTACCTTCTGTTGGGGATGTCTCTACCGGTTTGGCTATTTCCAAGACTCTGCGCAACATC

TCTTTCTGGCCCCTCTACCTTGCTGCCCTATTGTGGCATTCTGGC

>CIBHN201108149

GGTGACTTTGGTGGCTTTGGTCCTGCTGGGCATTATTTTCTCAGCCTTGTTCTTTTTCAT

GGACTCCACCACCTGGACCTCCTCTTTATTTTTCTACATGATGACCGCCGTTTTAGGTCT

AGGAGTCTTTGTGCCCTGGCTGCAGTACCTCATCAAGAGACATCCGCTCTTCTGGCTGGT

GGAGTTTCTGGTTCAGAGCAAAACACGACTGTATCTCCTCCTCTTCTGGACATGCCTTGT

TCTTGTTGCTTGCGGGATTGTTCTATTTCAAAATTCGAAGAGATCCACAGACTCCAAAAA

GCCGCAAGTATCCACCATAACCCGCAAGTACTTTCACTTTCTTGCAGTGGTTGTCTACGT

TCCTGGAGTCATGTACGATCGTTCTTTGCTCTATGTGGCTGCAGTGGTATGTTTGGCTGT

TTTTGTGCTATTGGAATACATTCGCTTCTTCCACATTAAGCCACTTGGACAGACCTTACG

GACTCTTCTGACGCTGTTCCTGGATGAGCGGGATAGCGGTCCACTGATTCTGTCACACAT

TTACCTTCTGTTGGGGATGTCTCTACCGGTTTGGCTCTTTCCAAGACTCTGCGCAACATC

TCTTTCTGGCCCCTCTACCTTGCTGCCCTATTGTGGCATTCTGGC

>CIBFJS20150501004

GGTGACTTTGGTGGCTTTGGTCCTGCTGGGCATTATTTTCTCAGCCTTGTTCTTTTTCAT

GGACTCCACCACCTGGACCTCCTCTTTATTTTTCTACATGATGACCGCCGTTTTAGGTCT

AGGAGTCTTTGTGCCCTGGCTGCAGTACCTCATCAAGAGACATCCGCTCTTCTGGCTGGT

GGAGTTTCTGGTTCAGAGCAAAACACGACTGTATCTCCTCCTCTTCTGGACATGCCTTGT

TCTTGTTGCTTGCGGGATTGTTCTATTTCAAAATTCGAAGAGATCCACAGACTCCAAAAA

GCCGCAAGTATCCACCATAACCCGCAAGTACTTTCACTTTCTTGCAGTGGTTGTCTACGT

TCCTGGAGTCATGTACGATCGTTCTTTGCTCTATGTGGCTGCAGTGGTATGTTTGGCTGT

TTTTGTGCTATTGGAATACATTCGCTTCTTCCACATTAAGCCACTTGGACAGACCTTACG

GACTCTTCTGACGCTGTTCCTGGATGAGCGGGATAGCGGTCCACTGATTCTGTCACACAT

TTACCTTCTGTTGGGGATGTCTCTACCGGTTTGGCTCTTTCCAAGACTCTGCGCAACATC

TCTTTCTGGCCCCTCTACCTTGCTGCCCTATTGTGGCATTCTGGC

>CIBFJS20150501006

GGTGACTTTGGTGGCTTTGGTCCTGCTGGGCATTATTTTCTCAGCCTTGTTCTTTTTCAT

GGACTCCACCACCTGGACCTCCTCTTTATTTTTCTACATGATGACCGCCGTTTTAGGTCT

AGGAGTCTTTGTGCCCTGGCTGCAGTACCTCATCAAGAGACATCCGCTCTTCTGGCTGGT

GGAGTTTCTGGTTCAGAGCAAAACACGACTGTATCTCCTCCTCTTCTGGACATGCCTTGT

TCTTGTTGCTTGCGGGATTGTTCTATTTCAAAATTCGAAGAGATCCACAGACTCCAAAAA

GCCGCAAGTATCCACCATAACCCGCAAGTACTTTCACTTTCTTGCAGTGGTTGTCTACGT

TCCTGGAGTCATGTACGATCGTTCTTTGCTCTATGTGGCTGCAGTGGTATGTTTGGCTGT

TTTTGTGCTATTGGAATACATTCGCTTCTTCCACATTAAGCCACTTGGACAGACCTTACG

GACTCTTCTGACGCTGTTCCTGGATGAGCGGGATAGCGGTCCACTGATTCTGTCACACAT

TTACCTTCTGTTGGGGATGTCTCTACCGGTTTGGCTCTTTCCAAGACTCTGCGCAACATC

TCTTTCTGGCCCCTCTACCTTGCTGCCCTATTGTGGCATTCTGGC

>CIBLS20140616004

GGTGACTTTGGTGGCTTTGGTCCTGCTGGGCATTATTTTCTCAGCCTTGTTCTTTTTCAT

GGACTCCACCACCTGGACATCCTCTTTATTTTTCTACATGATGACCGCCGTTTTAGGTCT

AGGAGTCTTTGTGCCCTGGCTGCAGTACCTCATCAAGAGACATCCGCTCTTCTGGCTGGT

GGAGTTTCTGGTTCAGAGCAAAACACGACTGTATCTCCTCCTCTTCTGGACATGCCTTGT

TCTTGTTGCTTGCGGGATTGTTCTATTTCAAAATTCGAAGAGATCCACAGACTCCAAAAA

GCCGCAAGTATCCACCATAACCCGCAAGTACTTTCACTTTCTTGCAGTGGTTGTCTACGT

TCCTGGAGTCATGTACGATCGTTCTTTGCTCTATGTGGCTGCAGTGGTATGTTTGGCTGT

TTTTGTGCTATTGGAATACGTTCGCTTCTTCCACATTAAGCCACTTGGACAGACCTTACG

AACTCTTCTGACGCTGTTCCTGGATGAGCGGGATAGCGGTCCACTGATTCTGTCACACAT

TTACCTTCTGTTGGGGATGTCTCTACCGGTTTGGCTCTTTCCAAGACTCTGCGCAACATC

TCTTTCTGGCCCCTCTACCTTGCTGCCCTATTGTGGCATTCTGGC

>CIBLS20140616006

GGTGACTTTGGTGGCTTTGGTCCTGCTGGGCATTATTTTCTCAGCCTTGTTCTTTTTCAT

GGACTCCACCACCTGGACATCCTCTTTATTTTTCTACATGATGACCGCCGTTTTAGGTCT

AGGAGTCTTTGTGCCCTGGCTGCAGTACCTCATCAAGAGACATCCGCTCTTCTGGCTGGT

GGAGTTTCTGGTTCAGAGCAAAACACGACTGTATCTCCTCCTCTTCTGGACATGCCTTGT

TCTTGTTGCTTGCGGGATTGTTCTATTTCAAAATTCGAAGAGATCCACAGACTCCAAAAA

GCCGCAAGTATCCACCATAACCCGCAAGTACTTTCACTTTCTTGCAGTGGTTGTCTACGT

TCCTGGAGTCATGTACGATCGTTCTTTGCTCTATGTGGCTGCAGTGGTATGTTTGGCTGT

TTTTGTGCTATTGGAATACGTTCGCTTCTTCCACATTAAGCCACTTGGACAGACCTTACG

AACTCTTCTGACGCTGTTCCTGGATGAGCGGGATAGCGGTCCACTGATTCTGTCACACAT

TTACCTTCTGTTGGGGATGTCTCTACCGGTTTGGCTCTTTCCAAGACTCTGCGCAACATC

TCTTTCTGGCCCCTCTACCTTGCTGCCCTATTGTGGCATTCTGGC

>CIBLS20140818005

GGTGACTTTGGTGGCTTTGGTCCTGCTGGGCATTATTTTCTCAGCCTTGTTCTTTTTCAT

GGACTCCACCACCTGGACCTCCTCTTTATTTTTCTACATGATGACTGCGGTTTTAGGTCT

AGGAGTCTTTGTGCCCTGGCTGCAGTACCTCATCAAGAGACATCCGCTCTTCTGGCTGGT

GGAGTTTCTGGTTCAGAGCAAAACACGACTGTATCTCCTCCTCTTCTGGACATGCCTTGT

TCTTGTTGCTTGCGGGATTGTTCTATTTCAAAATTCGAAGAGATCCACAGACTCCAAAAA

GCCGCAAGTATCCACCATAACCCGCAAGTACTTTCACTTTCTTGCAGTGGTTGTCTACGT

TCCTGGAGTCATGTACGATCGTTCTTTGCTCTATGTGGCTGCAGTGGTATGTTTGGCTGT

TTTTGTGCTATTGGAATACGTTCGCTTCTTCCACATTAAGCCACTTGGACAGACCTTACG

GACTCTTCTGACGCTGTTCCTGGATGAGCGGGATAGCGGTCCACTGATTCTGTCACACAT

TTACCTTCTGTTGGGGATGTCTCTACCGGTTTGGCTATTTCCAAGACTCTGCGCAACATC

TCTTTCTGGCCCCTCTACCTTGCTGCCCTATTGTGGCATTCTGGC

>CIBGD201108030

GGTGACTTTGGTGGCTTTGGTCCTGCTGGGCATTATTTTCTCAGCCTTGTTCTTTTTCAT

GGACTCCACCACCTGGACCTCCTCTTTATTTTTCTACATGATGACTGCGGTTTTAGGTCT

AGGAGTCTTTGTGCCCTGGCTGCAGTACCTCATCAAGAGACATCCGCTCTTCTGGCTGGT

GGAGTTTCTGGTTCAGAGCAAAACACGACTGTATCTCCTCCTCTTCTGGACATGCCTTGT

TCTTGTTGCTTGCGGGATTGTTCTATTTCAAAATTCGAAGAGATCCACAGACTCCAAAAA

GCCGCAAGTATCCACCATAACCCGCAAGTACTTTCACTTTCTTGCAGTGGTTGTCTACGT

TCCTGGAGTCATGTACGATCGTTCTTTGCTCTATGTGGCTGCAGTGGTATGTTTGGCTGT

TTTTGTGCTATTGGAATACGTTCGCTTCTTCCACATTAAGCCACTTGGACAGACCTTACG

GACTCTTCTGACGCTGTTCCTGGATGAGCGGGATAGCGGTCCACTGATTCTGTCACACAT

TTACCTTCTGTTGGGGATGTCTCTACCGGTTTGGCTATTTCCAAGACTCTGCGCAACATC

TCTTTCTGGCCCCTCTACCTTGCTGCCCTATTGTGGCATTCTGGC

>CIBFJS20150502002

GGTGACTTTGGTGGCTTTGGTCCTGCTGGGCATTATTTTCTCAGCCTTGTTCTTTTTCAT

GGACTCCACCACCTGGACCTCCTCTTTATTTTTCTACATGATGACTGCGGTTTTAGGTCT

AGGAGTCTTTGTGCCCTGGCTGCAGTACCTCATCAAGAGACATCCGCTCTTCTGGCTGGT

GGAGTTTCTGGTTCAGAGCAAAACACGACTGTATCTCCTCCTCTTCTGGACATGCCTTGT

TCTTGTTGCTTGCGGGATTGTTCTATTTCAAAATTCGAAGAGATCCACAGACTCCAAAAA

GCCGCAAGTATCCACCATAACCCGCAAGTACTTTCACTTTCTTGCAGTGGTTGTCTACGT

TCCTGGAGTCATGTACGATCGTTCTTTGCTCTATGTGGCTGCAGTGGTATGTTTGGCTGT

TTTTGTGCTATTGGAATACGTTCGCTTCTTCCACATTAAGCCACTTGGACAGACCTTACG

GACTCTTCTGACGCTGTTCCTGGATGAGCGGGATAGCGGTCCACTGATTCTGTCACACAT

TTACCTTCTGTTGGGGATGTCTCTACCGGTTTGGCTATTTCCAAGACTCTGCGCAACATC

TCTTTCTGGCCCCTCTACCTTGCTGCCCTATTGTGGCATTCTGGC
