# Supplementary material for: A new species of the odorous frog genus Odorrana (Amphibia, Anura, Ranidae) from southwestern China
Source: PeerJ. 2018 Oct 4;6:e5695. doi: 10.7717/peerj.5695 (PMC6174872; doi:10.7717/peerj.5695)
Supplement: Supplemental Information 7 — Voucher information for each sequence refer to Table S2. [file peerj-06-5695-s007.zip › raw data/KCNF gene sequence in this study.docx]

KCNF gene sequence in this study (sequence ID refer to Table 1 ):

>CIBGYU20130917004

CGAGATGGAGTTCTGGAAAGTGGACTTGTCTTTTCTGGATGAGTGCTGTAAGAGCCACCT

AAGTGAGAAGAAGGAGGAACTGGAGGAGATAGCTAAGAGGGTGCAGACCATCCTGGATGA

CCTAGGAGTTGACACCACCGAGAGCCGCTGGAAGAGGTTTCAGAAATATGTCTGGAAGTT

CATGGAGAAACCAGAATCCTCTTTTCCTGCCAGGGTCACAGCTGTGCTCTCCTTCCTATT

CATCCTGACCTCATCAGTGGTAATGTGTGTAGGGACCATTCCTGAAATGCAGGTGGAAGA

TGAAGAAGGGAACCCTGTAGAGCATCCTGTTCTAGACAACATAGAGACAGCATGCATTGG

CTGGTTTACCCTGGAATATATTCTAAGACTCTTGTCTTCCCCCAACAAGTTACACTTTAC

TTTTTCCTTCATGAATATCATTGACGTGCTGGCCATACTCCCATTCTATGTCAGCCTTAC

CCTGACCCATCTAGGGGCACGTATGATGGAATTGACTAATGTCCAGCAGGCTGTCCAGGC

CCTCAGGATCATGAGGATTGCCAGGATTTTTAAGCTGGCCCGCCATTCCTCTGGGCTGCA

GACCCTAACCTATGCCCTCAAGAGCAGTTTCAAGGAGTTGGGTCTGCTCTTGATGTACCT

TGCAGTGGGGATCTTTGTATTTTCAGCCCTAGGGTACACCATGGAACAGAGTCACCCAGA

TACCTTATTTAAAAGCATCCCTCAGTCTTTC

>CIBGYU20130921001

CGAGATGGAGTTCTGGAAAGTGGACTTGTCTTTTCTGGATGAGTGCTGTAAGAGCCACCT

AAGTGAGAAGAAGGAGGAACTGGAGGAGATAGCTAAGAGGGTGCAGACCATCCTGGATGA

CCTAGGAGTTGACACCACCGAGAGCCGCTGGAAGAGGTTTCAGAAATATGTCTGGAAGTT

CATGGAGAAACCAGAATCCTCTTTTCCTGCCAGGGTCACAGCTGTGCTCTCCTTCCTATT

CATCCTGACCTCATCAGTGGTAATGTGTGTAGGGACCATTCCTGAAATGCAGGTGGAAGA

TGAAGAAGGGAACCCTGTAGAGCATCCTGTTCTAGACAACATAGAGACAGCATGCATTGG

CTGGTTTACCCTGGAATATATTCTAAGACTCTTGTCTTCCCCCAACAAGTTACACTTTAC

TTTTTCCTTCATGAATATCATTGACGTGCTGGCCATACTCCCATTCTATGTCAGCCTTAC

CCTGACCCATCTAGGGGCACGTATGATGGAATTGACTAATGTCCAGCAGGCTGTCCAGGC

CCTCAGGATCATGAGGATTGCCAGGATTTTTAAGCTGGCCCGCCATTCCTCTGGGCTGCA

GACCCTAACCTATGCCCTCAAGAGCAGTTTCAAGGAGTTGGGTCTGCTCTTGATGTACCT

TGCAGTGGGGATCTTTGTATTTTCAGCCCTAGGGTACACCATGGAACAGAGTCACCCAGA

TACCTTATTTAAAAGCATCCCTCAGTCTTTC

>CIBGYU20130917005

CGAGATGGAGTTCTGGAAAGTGGACTTGTCTTTTCTGGATGAGTGCTGTAAGAGCCACCT

AAGTGAGAAGAAGGAGGAACTGGAGGAGATAGCTAAGAGGGTGCAGACCATCCTGGATGA

CCTAGGAGTTGACACCACCGAGAGCCGCTGGAAGAGGTTTCAGAAATATGTCTGGAAGTT

CATGGAGAAACCAGAATCCTCTTTTCCTGCCAGGGTCACAGCTGTGCTCTCCTTCCTATT

CATCCTGACCTCATCAGTGGTAATGTGTGTAGGGACCATTCCTGAAATGCAGGTGGAAGA

TGAAGAAGGGAACCCTGTAGAGCATCCTGTTCTAGACAACATAGAGACAGCATGCATTGG

CTGGTTTACCCTGGAATATATTCTAAGACTCTTGTCTTCCCCCAACAAGTTACACTTTAC

TTTTTCCTTCATGAATATCATTGACGTGCTGGCCATACTCCCATTCTATGTCAGCCTTAC

CCTGACCCATCTAGGGGCACGTATGATGGAATTGACTAATGTCCAGCAGGCTGTCCAGGC

CCTCAGGATCATGAGGATTGCCAGGATTTTTAAGCTGGCCCGCCATTCCTCTGGGCTGCA

GACCCTAACCTATGCCCTCAAGAGCAGTTTCAAGGAGTTGGGTCTGCTCTTGATGTACCT

TGCAGTGGGGATCTTTGTATTTTCAGCCCTAGGGTACACCATGGAACAGAGTCACCCAGA

TACCTTATTTAAAAGCATCCCTCAGTCTTTC

>CIBjs20150803008

CGAGATGGAGTTCTGGAAAGTGGACTTGTCTTTTCTGGATGAGTGCTGTAAGAGCCACCT

AAGTGAGAAGAAGGAGGAACTGGAGGAGATAGCTAAGAGGGTGCAGACCATCCTGGATGA

CCTAGGAGTTGACACCACCGAGAGCCGCTGGAAGAGGTTTCAGAAATATGTCTGGAAGTT

CATGGAGAAACCAGAATCCTCTTTTCCTGCCAGGGTCACAGCTGTGCTCTCCTTCCTATT

CATCCTGACCTCATCAGTGGTAATGTGTGTAGGGACCATTCCTGAAATGCAGGTGGAAGA

TGAAGAAGGGAACCCTGTAGAGCATCCTGTTCTAGACAACATAGAGACAGCATGCATTGG

CTGGTTTACCCTGGAATATATTCTAAGACTCTTGTCTTCCCCCAACAAGTTACACTTTAC

TTTTTCCTTCATGAATATCATTGACGTGCTGGCCATACTCCCATTCTATGTCAGCCTTAC

CCTGACCCATCTAGGGGCACGTATGATGGAATTGACTAATGTCCAGCAGGCTGTCCAGGC

CCTCAGGATCATGAGGATTGCCAGGATTTTTAAGCTGGCCCGCCATTCCTCTGGGCTGCA

GACCCTAACCTATGCCCTCAAGAGCAGTTTCAAGGAGTTGGGTCTGCTCTTGATGTACCT

TGCAGTGGGGATCTTTGTATTTTCAGCCCTAGGGTACACCATGGAACAGAGTCACCCAGA

TACCTTATTTAAAAGCATCCCTCAGTCTTTC

>CIBjs20171014001

CGAGATGGAGTTCTGGAAAGTGGACTTGTCTTTTCTGGATGAGTGCTGTAAGAGCCACCT

AAGTGAGAAGAAGGAGGAACTGGAGGAGATAGCTAAGAGGGTGCAGACCATCCTGGATGA

CCTAGGAGTTGACACCACCGAGAGCCGCTGGAAGAGGTTTCAGAAATATGTCTGGAAGTT

CATGGAGAAACCAGAATCCTCTTTTCCTGCCAGGGTCACAGCTGTGCTCTCCTTCCTATT

CATCCTGACCTCATCAGTGGTAATGTGTGTAGGGACCATTCCTGAAATGCAGGTGGAAGA

TGAAGAAGGGAACCCTGTAGAGCATCCTGTTCTAGACAACATAGAGACAGCATGCATTGG

CTGGTTTACCCTGGAATATATTCTAAGACTCTTGTCTTCCCCCAACAAGTTACACTTTAC

TTTTTCCTTCATGAATATCATTGACGTGCTGGCCATACTCCCATTCTATGTCAGCCTTAC

CCTGACCCATCTAGGGGCACGTATGATGGAATTGACTAATGTCCAGCAGGCTGTCCAGGC

CCTCAGGATCATGAGGATTGCCAGGATTTTTAAGCTGGCCCGCCATTCCTCTGGGCTGCA

GACCCTAACCTATGCCCTCAAGAGCAGTTTCAAGGAGTTGGGTCTGCTCTTGATGTACCT

TGCAGTGGGGATCTTTGTATTTTCAGCCCTAGGGTACACCATGGAACAGAGTCACCCAGA

TACCTTATTTAAAAGCATCCCTCAGTCTTTC

>CIB20130531

CGAGATGGAGTTCTGGAAAGTGGACTTGTCTTTTCTGGATGAGTGCTGTAAGAGCCACCT

AAGTGAGAAGAAGGAGGAACTGGAGGAGATAGCTAAGAGGGTGCAGACCATCCTGGATGA

CCTAGGAGTTGACACCACCGAGAGCCGCTGGAAGAGGTTTCAGAAATATGTCTGGAAGTT

CATGGAGAAACCAGAATCCTCTTTTCCTGCTAGGGTCACAGCTGTGCTCTCCTTCCTATT

CATCCTGACCTCATCAGTGGTAATGTGTGTAGGGACCATTCCTGAAATGCAGGTGGAAGA

TGCAGAAGGGAACCATGTAGAGCATCCTGCTCTAGACAACATAGAGACAGCATGCATTGG

CTGGTTTACCCTGGAATATATTCTAAGACTCTTGTCTTCCCCCAACAAGTTACACTTTAC

TTTTTCCTTCATGAATATCATTGACGTGCTGGCCATACTCCCATTCTATGTCAGCCTTAC

CCTGACCCATCTAGGGGCACGCATGATGGAATTGACTAATGTCCAGCAGGCTGTCCAGGC

CCTCAGGATCATGAGGATTGCCAGGATTTTTAAGCTGGCCCGCCATTCCTCTGGGCTGCA

GACCCTAACCTATGCCCTCAAGAGCAGTTTCAAGGAGCTGGGTCTGCTCTTGATGTACCT

TGCAGTGGGGATCTTTGTATTTTCAGCCCTAGGGTACACCATGGAACAGAGTCACCCAGA

TACCTTATTTAAAAGCATCCCTCAGTCTTTC

>CIB20130532

CGAGATGGAGTTCTGGAAAGTGGACTTGTCTTTTCTGGATGAGTGCTGTAAGAGCCACCT

AAGTGAGAAGAAGGAGGAACTGGAGGAGATAGCTAAGAGGGTGCAGACCATCCTGGATGA

CCTAGGAGTTGACACCACCGAGAGCCGCTGGAAGAGGTTTCAGAAATATGTCTGGAAGTT

CATGGAGAAACCAGAATCCTCTTTTCCTGCTAGGGTCACAGCTGTGCTCTCCTTCCTATT

CATCCTGACCTCATCAGTGGTAATGTGTGTAGGGACCATTCCTGAAATGCAGGTGGAAGA

TGCAGAAGGGAACCATGTAGAGCATCCTGCTCTAGACAACATAGAGACAGCATGCATTGG

CTGGTTTACCCTGGAATATATTCTAAGACTCTTGTCTTCCCCCAACAAGTTACACTTTAC

TTTTTCCTTCATGAATATCATTGACGTGCTGGCCATACTCCCATTCTATGTCAGCCTTAC

CCTGACCCATCTAGGGGCACGCATGATGGAATTGACTAATGTCCAGCAGGCTGTCCAGGC

CCTCAGGATCATGAGGATTGCCAGGATTTTTAAGCTGGCCCGCCATTCCTCTGGGCTGCA

GACCCTAACCTATGCCCTCAAGAGCAGTTTCAAGGAGCTGGGTCTGCTCTTGATGTACCT

TGCAGTGGGGATCTTTGTATTTTCAGCCCTAGGGTACACCATGGAACAGAGTCACCCAGA

TACCTTATTTAAAAGCATCCCTCAGTCTTTC

>CIB20130533

CGAGATGGAGTTCTGGAAAGTGGACTTGTCTTTTCTGGATGAGTGCTGTAAGAGCCACCT

AAGTGAGAAGAAGGAGGAACTGGAGGAGATAGCTAAGAGGGTGCAGACCATCCTGGATGA

CCTAGGAGTTGACACCACCGAGAGCCGCTGGAAGAGGTTTCAGAAATATGTCTGGAAGTT

CATGGAGAAACCAGAATCCTCTTTTCCTGCTAGGGTCACAGCTGTGCTCTCCTTCCTATT

CATCCTGACCTCATCAGTGGTAATGTGTGTAGGGACCATTCCTGAAATGCAGGTGGAAGA

TGCAGAAGGGAACCATGTAGAGCATCCTGCTCTAGACAACATAGAGACAGCATGCATTGG

CTGGTTTACCCTGGAATATATTCTAAGACTCTTGTCTTCCCCCAACAAGTTACACTTTAC

TTTTTCCTTCATGAATATCATTGACGTGCTGGCCATACTCCCATTCTATGTCAGCCTTAC

CCTGACCCATCTAGGGGCACGCATGATGGAATTGACTAATGTCCAGCAGGCTGTCCAGGC

CCTCAGGATCATGAGGATTGCCAGGATTTTTAAGCTGGCCCGCCATTCCTCTGGGCTGCA

GACCCTAACCTATGCCCTCAAGAGCAGTTTCAAGGAGCTGGGTCTGCTCTTGATGTACCT

TGCAGTGGGGATCTTTGTATTTTCAGCCCTAGGGTACACCATGGAACAGAGTCACCCAGA

TACCTTATTTAAAAGCATCCCTCAGTCTTTC

>CIBLC2010092

CGAGATGGAGTTCTGGAAAGTGGACTTGTCTTTTCTGGATGAGTGCTGTAAGAGCCACCT

AAGTGAGAAGAAGGAGGAACTGGAGGAGATAGCTAAGAGGGTGCAGACCATCCTGGATGA

CCTAGGAGTTGACACCACCGAGAGCCGCTGGAAGAGGTTTCAGAAATATGTCTGGAAGTT

CATGGAGAAACCAGAATCCTCTTTTCCTGCCAGGGTCACAGCTGTGCTCTCCTTCCTATT

TATCCTGACCTCATCAGTGGTAATGTGTGTAGGGACCATTCCTGAAATGCAGGTGGAAGA

TGCAGAAGGGAACCATGTAGAGCATCCTGTTCTAGACAACATAGAGACAGCATGCATTGG

CTGGTTTACCCTGGAATATATTCTAAGACTCTTGTCTTCCCCCAACAAGTTACACTTTAC

TTTTTCCTTCATGAATATCATTGACGTGCTGGCCATACTCCCATTCTATGTCAGCCTTAC

CTTGACCCATCTAGGGGCACGCATGATGGAATTGACTAATGTCCAGCAGGCTGTCCAGGC

CCTCAGGATCATGAGGATTGCCAGGATTTTTAAGCTGGCCCGCCATTCCTCTGGGCTGCA

GACCCTAACCTATGCCCTCAAGAGCAGTTTCAAGGAGTTGGGTCTGCTCTTGATGTACCT

TGCAGTGGGGATCTTTGTATTTTCAGCCCTAGGGTACACCATGGAACAGAGTCACCCAGA

TACCTTATTTAAAAGCATCCCTCAGTCTTTC

>CIBLC2010097

CGAGATGGAGTTCTGGAAAGTGGACTTGTCTTTTCTGGATGAGTGCTGTAAGAGCCACCT

AAGTGAGAAGAAGGAGGAACTGGAGGAGATAGCTAAGAGGGTGCAGACCATCCTGGATGA

CCTAGGAGTTGACACCACCGAGAGCCGCTGGAAGAGGTTTCAGAAATATGTCTGGAAGTT

CATGGAGAAACCAGAATCCTCTTTTCCTGCCAGGGTCACAGCTGTGCTCTCCTTCCTATT

TATCCTGACCTCATCAGTGGTAATGTGTGTAGGGACCATTCCTGAAATGCAGGTGGAAGA

TGCAGAAGGGAACCATGTAGAGCATCCTGTTCTAGACAACATAGAGACAGCATGCATTGG

CTGGTTTACCCTGGAATATATTCTAAGACTCTTGTCTTCCCCCAACAAGTTACACTTTAC

TTTTTCCTTCATGAATATCATTGACGTGCTGGCCATACTCCCATTCTATGTCAGCCTTAC

CTTGACCCATCTAGGGGCACGCATGATGGAATTGACTAATGTCCAGCAGGCTGTCCAGGC

CCTCAGGATCATGAGGATTGCCAGGATTTTTAAGCTGGCCCGCCATTCCTCTGGGCTGCA

GACCCTAACCTATGCCCTCAAGAGCAGTTTCAAGGAGTTGGGTCTGCTCTTGATGTACCT

TGCAGTGGGGATCTTTGTATTTTCAGCCCTAGGGTACACCATGGAACAGAGTCACCCAGA

TACCTTATTTAAAAGCATCCCTCAGTCTTTC

>CIBHN201108149

TGAGATGGAGTTCTGGAAAGTGGACTTGTCTTTTCTGGATGAGTGCTGTAAGAGCCACCT

AAGTGAGAAGAAGGAGGAACTGGAAGAGATAGCTAAGAGGGTGCAGACCATCCTGGATGA

CCTAGGAGTTGACACCACCGAGAGCCGCTGGAAGAGGTTTCAGAAATATGTCTGGAAGTT

CATGGAGAAGCCAGAATCCTCTTTTCCTGCCAGGGTCACAGCTGTGCTCTCCTTCCTATT

CATCCTGACCTCATCAGTGGTAATGTGTGTAGGGACCATTCCTGAAATGCAGGTGGAAGA

TGCAGAAGGGAACCATGTAGAGCATCCTGTTCTAGACAACATAGAGACAGCATGCATTGG

CTGGTTTACCCTGGAATATATTCTAAGACTCTTGTCTTCCCCCAACAAGTTACACTTTAC

TTTTTCCTTCATGAATATCATTGACGTGCTGGCCATACTCCCATTCTATGTCAGCCTTAC

CCTGACCCATCTAGGGGCACGCATGATGGAATTGACTAATGTCCAGCAGGCTGTCCAGGC

CCTCAGGATCATGAGGATTGCCAGGATTTTTAAGCTGGCCCGCCATTCCTCTGGGCTGCA

GACCCTAACCTATGCCCTCAAGAGCAGTTTCAAGGAGTTGGGTCTGCTTTTGATGTACCT

TGCAGTGGGGATCTTTGTATTTTCAGCCCTAGGGTACACCATGGAACAGAGTCACCCAGA

TACCTTATTTAAAAGCATCCCTCAGTCTTTC

>CIBFJS20150501004

CGAGATGGAGTTCTGGAAAGTGGACTTGTCTTTTCTGGATGAGTGCTGTAAGAGCCACCT

AAGTGAGAAGAAGGAGGAACTGGAAGAGATCGCTAAGAGGGTGCAGACCATCCTGGATGA

CCTAGGAGTTGACACCACCGAGAGCCGCTGGAAGAGGTTTCAGAAATATGTCTGGAAGTT

CATGGAGAAGCCAGAATCCTCTTTTCCTGCCAGGGTCACAGCTGTGCTCTCCTTCCTATT

CATCCTGACCTCATCAGTGGTAATGTGTGTAGGGACCATTCCTGAAATGCAGGTGGAAGA

TACAGAAGGGAACCATGTAGAGCATCCTGTTCTAGACAACATAGAGACAGCATGCATTGG

CTGGTTTACCCTGGAATATATTCTAAGACTCTTGTCTTCCCCCAACAAGTTACACTTTAC

TTTTTCCTTCATGAATATCATTGACGTGCTGGCCATACTCCCATTCTATGTCAGCCTTAC

CCTGACCCATCTAGGGGCACGCATGATGGAATTGACTAATGTCCAGCAGGCTGTCCAGGC

CCTCAGGATCATGAGGATTGCCAGGATTTTTAAGCTGGCCCGCCATTCCTCTGGGCTGCA

GACCCTAACCTATGCCCTCAAGAGCAGTTTCAAGGAGTTGGGTCTGCTTTTGATGTACCT

TGCAGTGGGGATCTTTGTATTTTCAGCCCTAGGGTACACCATGGAACAGAGTCACCCAGA

TACCTTATTTAAAAGCATCCCTCAGTCTTTC

>CIBFJS20150501006

CGAGATGGAGTTCTGGAAAGTGGACTTGTCTTTTCTGGATGAGTGCTGTAAGAGCCACCT

AAGTGAGAAGAAGGAGGAACTGGAAGAGATCGCTAAGAGGGTGCAGACCATCCTGGATGA

CCTAGGAGTTGACACCACCGAGAGCCGCTGGAAGAGGTTTCAGAAATATGTCTGGAAGTT

CATGGAGAAGCCAGAATCCTCTTTTCCTGCCAGGGTCACAGCTGTGCTCTCCTTCCTATT

CATCCTGACCTCATCAGTGGTAATGTGTGTAGGGACCATTCCTGAAATGCAGGTGGAAGA

TGCAGAAGGGAACCATGTAGAGCATCCTGTTCTAGACAACATAGAGACAGCATGCATTGG

CTGGTTTACCCTGGAATATATTCTAAGACTCTTGTCTTCCCCCAACAAGTTACACTTTAC

TTTTTCCTTCATGAATATCATTGACGTGCTGGCCATACTCCCATTCTATGTCAGCCTTAC

CCTGACCCATCTAGGGGCACGCATGATGGAATTGACTAATGTCCAGCAGGCTGTCCAGGC

CCTCAGGATCATGAGGATTGCCAGGATTTTTAAGCTGGCCCGCCATTCCTCTGGGCTGCA

GACCCTAACCTATGCCCTCAAGAGCAGTTTCAAGGAGTTGGGTCTGCTTTTGATGTACCT

TGCAGTGGGGATCTTTGTATTTTCAGCCCTAGGGTACACCATGGAACAGAGTCACCCAGA

TACCTTATTTAAAAGCATCCCTCAGTCTTTC

>CIBLS20140616004

CGAGATGGAGTTCTGGAAAGTGGACTTGTCTTTTCTGGATGAGTGCTGTAAGAGCCACCT

AAGTGAGAAGAAGGAGGAACTGGAAGAGATAGCTAAGAGGGTGCAGACCATCCTGGATGA

CCTAGGAGTTGACACCACCGAGAGCCGCTGGAAGAGGTTTCAGAAATATGTCTGGAAGTT

CATGGAGAAGCCAGAATCCTCTTTTCCTGCCAGGGTCACAGCTGTGCTCTCCTTCCTATT

CATCCTGACCTCATCAGTGGTAATGTGTGTAGGGACCATTCCTGAAATGCAGGTGGAAGA

TGCAGAAGGGAACCATGTAGAGCATCCTGTTCTAGACAACATAGAGACAGCATGCATTGG

CTGGTTTACCCTGGAATATATTCTAAGACTCTTGTCTTCCCCCAACAAGTTACACTTTAC

TTTTTCCTTCATGAATATCATTGACGTGCTGGCCATACTCCCATTCTATGTCAGCCTTAC

CCTGACCCATCTAGGGGCACGCATGATGGAATTGACTAATGTCCAGCAGGCTGTCCAGGC

CCTCAGGATCATGAGGATTGCCAGGATTTTTAAGCTGGCCCGCCATTCCTCTGGGCTGCA

GACCCTAACCTATGCCCTCAAGAGCAGTTTCAAGGAGTTGGGTCTGCTTTTGATGTACCT

TGCAGTTGGGATCTTTGTATTTTCAGCCCTAGGGTACACCATGGAACAGAGTCACCCAGA

TACCTTATTTAAAAGCATCCCTCAGTCTTTC

>CIBLS20140616006

CGAGATGGAGTTCTGGAAAGTGGACTTGTCTTTTCTGGATGAGTGCTGTAAGAGCCACCT

AAGTGAGAAGAAGGAGGAACTGGAAGAGATAGCTAAGAGGGTGCAGACCATCCTGGATGA

CCTAGGAGTTGACACCACCGAGAGCCGCTGGAAGAGGTTTCAGAAATATGTCTGGAAGTT

CATGGAGAAGCCAGAATCCTCTTTTCCTGCCAGGGTCACAGCTGTGCTCTCCTTCCTATT

CATCCTGACCTCATCAGTGGTAATGTGTGTAGGGACCATTCCTGAAATGCAGGTGGAAGA

TGCAGAAGGGAACCATGTAGAGCATCCTGTTCTAGACAACATAGAGACAGCATGCATTGG

CTGGTTTACCCTGGAATATATTCTAAGACTCTTGTCTTCCCCCAACAAGTTACACTTTAC

TTTTTCCTTCATGAATATCATTGACGTGCTGGCCATACTCCCATTCTATGTCAGCCTTAC

CCTGACCCATCTAGGGGCACGCATGATGGAATTGACTAATGTCCAGCAGGCTGTCCAGGC

CCTCAGGATCATGAGGATTGCCAGGATTTTTAAGCTGGCCCGCCATTCCTCTGGGCTGCA

GACCCTAACCTATGCCCTCAAGAGCAGTTTCAAGGAGTTGGGTCTGCTTTTGATGTACCT

TGCAGTTGGGATCTTTGTATTTTCAGCCCTAGGGTACACCATGGAACAGAGTCACCCAGA

TACCTTATTTAAAAGCATCCCTCAGTCTTTC

>CIBLS20140818005

CGAGATGGAGTTCTGGAAAGTGGACTTGTCTTTTCTGGATGAGTGCTGTAAGAGCCACCT

AAGTGAGAAGAAGGAGGAACTGGAGGAGATAGCTAAGAGGGTGCAGACCATCCTGGATGA

CCTAGGAGTTGACACCACCGAGAGCCGTTGGAAGAGGTTTCAGAAATATGTCTGGAAGTT

CATGGAGAAACCAGAATCCTCTTTTCCTGCCAGGGTCACAGCTGTGCTCTCCTTCCTATT

CATCCTGACCTCATCAGTGGTAATGTGTGTAGGGACCATTCCTGAAATGCAGGTGGAAGA

TGAAGAAGGGAACCATGTAGAGCATCCTGTTCTAGACAACATAGAGACAGCATGCATTGG

CTGGTTTACCCTGGAATATATTCTAAGACTCTTGTCTTCCCCCAACAAGTTACACTTTAC

TTTTTCCTTCATGAATATCATTGACGTGCTGGCCATACTCCCATTCTATGTCAGCCTTAC

CCTGACCCATCTAGGGGCACGCATGATGGAATTGACTAATGTCCAGCAGGCTGTCCAGGC

CCTCAGGATCATGAGGATTGCCAGGATTTTTAAGCTGGCCCGCCATTCCTCTGGGCTGCA

GACCCTAACCTATGCCCTCAAGAGCAGTTTCAAGGAGTTGGGTCTGCTCTTGATGTACCT

TGCAGTGGGGATCTTTGTATTTTCAGCCCTAGGGTACACCATGGAACAGAGTCACCCAGA

TACCTTATTTAAAAGCATCCCTCAGTCTTTC

>CIBGD201108030

CGAGATGGAGTTCTGGAAAGTGGACTTGTCTTTTCTGGATGAGTGCTGTAAGAGCCACCT

AAGTGAGAAGAAGGAGGAACTGGAGGAGATAGCTAAGAGGGTGCAGACCATCCTGGATGA

CCTAGGAGTTGACACCACCGAGAGCCGCTGGAAGAGGTTTCAGAAATATGTCTGGAAGTT

CATGGAGAAACCAGAATCCTCTTTTCCTGCCAGGGTCACAGCTGTGCTCTCCTTCCTATT

CATCCTGACCTCATCAGTGGTAATGTGTGTAGGGACCATTCCTGAAATGCAGGTGGAAGA

TGAAGAAGGGAACCATGTAGAGCATCCTGTTCTAGACAACATAGAGACAGCATGCATTGG

CTGGTTTACCCTGGAATATATTCTAAGACTCTTGTCTTCCCCCAACAAGTTACACTTTAC

TTTTTCCTTCATGAATATCATTGACGTGCTGGCCATACTCCCATTCTATGTCAGCCTTAC

CCTGACCCATCTAGGGGCACGCATGATGGAATTGACTAATGTCCAGCAGGCTGTCCAGGC

CCTCAGGATCATGAGGATTGCCAGGATTTTTAAGCTGGCCCGCCATTCCTCTGGGCTGCA

GACCCTAACCTATGCCCTCAAGAGCAGTTTCAAGGAGTTGGGTCTGCTCTTGATGTACCT

TGCAGTGGGGATCTTTGTATTTTCAGCCCTAGGGTACACCATGGAACAGAGTCACCCAGA

TACCTTATTTAAAAGCATCCCTCAGTCTTTC

>CIBFJS20150502002

CGAGATGGAGTTCTGGAAAGTGGACTTGTCTTTTCTGGATGAGTGCTGTAAGAGCCACCT

AAGTGAGAAGAAGGAGGAACTGGAGGAGATAGCTAAGAGGGTGCAGACCATCCTGGATGA

CCTAGGAGTTGATACCACTGAGAGCCGCTGGAAGAGGTTTCAGAAATATGTCTGGAAGTT

CATGGAGAAACCAGAATCCTCTTTTCCTGCCAGGGTCACAGCTGTGCTCTCCTTCCTATT

TATCCTGACCTCATCAGTGGTAATGTGTGTAGGGACCATTCCTGAAATGCAGGTGGAAGA

TACAGAAGGAAACCATGTAGAGCATCCTGTTCTAGACAACATAGAGACAGCATGCATTGG

CTGGTTTACCCTGGAATATATTCTAAGACTCTTGTCTTCCCCCAACAAGTTACACTTTAC

TTTTTCCTTCATGAATATCATTGACGTGCTGGCCATACTCCCATTCTATGTCAGCCTTAC

CTTGACCCATCTAGGGGCACGCATGATGGAATTGACTAATGTCCAGCAGGCTGTCCAGGC

CCTCAGGATCATGAGGATTGCCAGGATTTTTAAGCTGGCCCGCCATTCCTCTGGGCTGCA

GACCCTAACCTATGCCCTCAAGAGCAGTTTCAAGGAGTTGGGTCTGCTCTTGATGTACCT

TGCAGTGGGGATCTTTGTATTTTCAGCCCTAGGGTACACCATGGAACAGAGTCACCCAGA

TACCTTATTTAAAAGCATCCCTCAGTCTTTC
