# Supplementary material for: A new species of the odorous frog genus Odorrana (Amphibia, Anura, Ranidae) from southwestern China
Source: PeerJ. 2018 Oct 4;6:e5695. doi: 10.7717/peerj.5695 (PMC6174872; doi:10.7717/peerj.5695)
Supplement: Supplemental Information 7 — Voucher information for each sequence refer to Table S2. [file peerj-06-5695-s007.zip › raw data/ND2 sequence in this study.docx]

ND2 sequence in this study (sequence ID refer to Table 1 ):

>CIBGYU20130917004

ATTAACCCACTCGCCCTTGTAATATTTCTTATTAGCCTTGCCATCGGAACCACCACTACT

ATATCAAGTCATCACTGACTTCTTGCTTGAATCGGATTAGAAATTAACACCTTAGCCTTA

CTTCCCATCATAATGAAAACACCCCACCCACGAGCCATTGAAGCAGCCACAAAATACTTC

TTAACTCAAGCTACAGCTTCTGCCTTAATATTATTTTCATGCCTCATCAACGCCCTGCAA

ACTGGAGAATGAAACATCAACAACCCCCCCAATTTAATAACAAACCCCCTCTCCCTAGCC

ATTATAATAAAACTAGGATTAGCCCCCTTACACTTTTGACTGCCAGAAGTTCTTCAAGGA

ACCCCACTATCAACAGGACTCATCTTATCTACCTGACAAAAAATCCCCCCTATAATCCTC

CTACTTCAAATTTCCCCCGACATTAACCTAAGCCTGACAGTCATCATGGGTCTTACTTCA

ATTTTAATCGGGGGCTGAGGCGGGATCGGCCAAACCCAACTTCGAAAAATCATAGCCTTC

TCCTCAATTGGCCACCTTGGATGAATCATCCTCATTTTAAAGCTTAACCCCCAGCTCTCC

CTATTTAGCTTCATCCTATATATTATTATAACAACTGCCATATTCCTTTCACTAATCATA

CTCTCCACAACAAAAATATCACAAATCTCAATCTCATGAACCAAAAACCCTGCCATAACT

ACAACTGCCCTGGTCATCCTCCTCTCCTTAGCAGGACTACCCCCTCTC

>CIBGYU20130921001

ATTAACCCACTCGCCCTTGTAATATTTCTTATTAGCCTTGCCATCGGAACCACCACTACT

ATATCAAGTCATCACTGACTTCTTGCTTGAATCGGATTAGAAATTAACACCTTAGCCTTA

CTTCCCATCATAATGAAAACACCCCACCCACGAGCCATTGAAGCAACCACAAAATACTTC

TTAACTCAAGCTACAGCTTCTGCCTTAATATTATTTTCATGCCTCATCAACGCCCTGCAA

ACTGGAGAATGAAACATCAACAACCCCCCCAATTTAATAACAAACCCCCTCTCCCTAGCC

ATTATAATAAAACTAGGATTAGCCCCCTTACACTTTTGACTGCCAGAAGTTCTTCAAGGA

ACCCCACTATCAACAGGACTCATCTTATCTACCTGACAAAAAATCCCCCCTATAATCCTC

CTACTTCAAATTTCCCCCGACATTAACCTAAGCCTGACAGTCATCATGGGTCTTACTTCA

ATTTTAATCGGGGGCTGAGGCGGGATCGGCCAAACCCAACTTCGAAAAATCATAGCCTTC

TCCTCAATTGGCCACCTTGGATGAATCATCCTCATTTTAAAGCTTAACCCCCAGCTCTCC

CTATTTAGCTTCATCCTATATATTATTATAACAACTGCCATATTCCTTTCACTAATCATA

CTCTCCACAACAAAAATATCACAAATCTCAATCTCATGAACCAAAAACCCTGCCATAACT

ACAACTGCCCTGCTCATCCTCCTCTCCTTAGCAGGACTACCCCCTCTC

>CIBGYU20130917005

ATTAACCCACTCGCCCTTGTAATATTTCTTATTAGCCTTGCCATCGGAACCACCACTACT

ATATCAAGTCATCACTGACTTCTTGCTTGAATCGGATTAGAAATTAACACCTTAGCCTTA

CTTCCCATCATAATGAAAACACCCCACCCACGAGCCATTGAAGCAACCACAAAATACTTC

TTAACTCAAGCTACAGCTTCTGCCTTAATATTATTTTCATGCCTCATCAACGCCCTGCAA

ACTGGAGAATGAAACATCAACAACCCCCCCAATTTAATAACAAACCCCCTCTCCCTAGCC

ATTATAATAAAACTAGGATTAGCCCCCTTACACTTTTGACTGCCAGAAGTTCTTCAAGGA

ACCCCACTATCAACAGGACTCATCTTATCTACCTGACAAAAAATCCCCCCTATAATCCTC

CTACTTCAAATTTCCCCCGACATTAACCTAAGCCTGACAGTCATCATGGGTCTTACTTCA

ATTTTAATCGGGGGCTGAGGCGGGATCGGCCAAACCCAACTTCGAAAAATCATAGCCTTC

TCCTCAATTGGCCACCTTGGATGAATCATCCTCATTTTAAAGCTTAACCCCCAGCTCTCC

CTATTTAGCTTCATCCTATATATTATTATAACAACTGCCATATTCCTTTCACTAATCATA

CTCTCCACAACAAAAATATCACAAATCTCAATCTCATGAACCAAAAACCCTGCCATAACT

ACAACTGCCCTGCTCATCCTCCTCTCCTTAGCAGGACTACCCCCTCTC

>CIBjs20150803008

ATTAACCCACTCGCCCTTGTAATATTTCTTATTAGCCTTGCCATCGGAACCACCACTACT

ATATCAAGTCATCACTGACTTCTTGCTTGAATCGGATTAGAAATTAACACCTTAGCCTTA

CTTCCCATCATAATGAAAACACCCCACCCACGAGCCATTGAAGCAACCACAAAATACTTC

TTAACTCAAGCTACAGCTTCTGCCTTAATATTATTTTCATGCCTCATCAACGCCCTGCAA

ACTGGAGAATGAAACATCAACAACCCCCCCAATTTAATAACAAACCCCCTCTCCCTAGCC

ATTATAATAAAACTAGGATTAGCCCCCTTACACTTTTGACTGCCAGAAGTTCTTCAAGGA

ACCCCACTATCAACAGGACTCATCTTATCTACCTGACAAAAAATCCCCCCTATAATCCTC

CTACTTCAAATTTCCCCCGACATTAACCTAAGCCTGACAGTCATCATGGGTCTTACTTCA

ATTTTAATCGGGGGCTGAGGCGGGATCGGCCAAACCCAACTTCGAAAAATCATAGCCTTC

TCCTCAATTGGCCACCTTGGATGAATCATCCTCATTTTAAAGCTTAACCCCCAGCTCTCC

CTATTTAGCTTCATCCTATATATTATTATAACAACTGCCATATTCCTTTCACTAATCATA

CTCTCCACAACAAAAATATCACAAATCTCAATCTCATGAACCAAAAACCCTGCCATAACT

ACAACTGCCCTGCTCATCCTCCTCTCCTTAGCAGGACTACCCCCTCTC

>CIBjs20171014001

ATTAACCCACTCGCCCTTGTAATATTTCTTATTAGCCTTGCCATCGGAACCACCACTACT

ATATCAAGTCATCACTGACTTCTTGCTTGAATCGGATTAGAAATTAACACCTTAGCCTTA

CTTCCCATCATAATGAAAACACCCCACCCACGAGCCATTGAAGCAACCACAAAATACTTC

TTAACTCAAGCTACAGCTTCTGCCTTAATATTATTTTCATGCCTCATCAACGCCCTGCAA

ACTGGAGAATGAAACATCAACAACCCCCCCAATTTAATAACAAACCCCCTCTCCCTAGCC

ATTATAATAAAACTAGGATTAGCCCCCTTACACTTTTGACTGCCAGAAGTTCTTCAAGGA

ACCCCACTATCAACAGGACTCATCTTATCTACCTGACAAAAAATCCCCCCTATAATCCTC

CTACTTCAAATTTCCCCCGACATTAACCTAAGCCTGACAGTCATCATGGGTCTTACTTCA

ATTTTAATCGGGGGCTGAGGCGGGATCGGCCAAACCCAACTTCGAAAAATCATAGCCTTC

TCCTCAATTGGCCACCTTGGATGAATCATCCTCATTTTAAAGCTTAACCCCCAGCTCTCC

CTATTTAGCTTCATCCTATATATTATTATAACAACTGCCATATTCCTTTCACTAATCATA

CTCTCCACAACAAAAATATCACAAATCTCAATCTCATGAACCAAAAACCCTGCCATAACT

ACAACTGCCCTGCTCATCCTCCTCTCCTTAGCAGGACTACCCCCTCTC

>CIB20130531

ATTAACCCACTCGCCCTTATAGTATTTCTTATTAGCCTTGCCATCGGAGCCACCACTACT

ATATTAAGTCACCACTGACTTCTTGCCTGAATCGGATTAGAGATTAATACCTTAGCTTTA

CTCCCCATCATAATGAAAACACCCCACCCACGAGCCATTGAAGCAGCCACAAAATATTTC

TTAACTCAAGCTACAGCTTCTGCCTTAATACTATTTTCATGCCTCATCAACGCCCTGCAA

ACTGGAGAATGAAATATCAACAACCCCCACAATTTAATAACAACTCCCCTGTCCCTAGCT

ATTATAATAAAATTAGGACTAGCCCCCCTACACTTTTGACTGCCAGAAGTTCTCCAAGGA

ACCCCCCTGTCAACAGGACTCATCTTATCTACCTGACAAAAAATTCCCCCCATAATCCTC

CTGCTTCAAATTTCCCCCAACATTAACCTAAGCCTAACAATTATCATGGGCCTTACTTCA

ATTTTAATCGGGGGCTGAGGCGGGATCGGCCAAACCCAACTTCGAAAAATCATAGCCTTC

TCCTCAATTGGCCACCTTGGATGAATCATCCTCATCTTAAAGCTTAACCCACAGCTCTCC

CTGTTTAGCTTCATCCTATACATCATTATAACAACTGCTATATTCCTTTCACTAATTATG

CTCTCCACCACAAAAATATCACAAATCTCAATCTCATGAACTAAAAACCCTGCCATAATA

ACAACCACCATGCTCATCCTCCTCTCTCTAGCAGGACTACCCCCTCTC

>CIB20130532

ATTAACCCACTCGCCCTTATAGTATTTCTTATTAGCCTTGCCATCGGAACCACCACTACT

ATATTAAGTCACCACTGACTTCTTGCCTGAATCGGATTAGAGATTAATACCTTAGCTTTA

CTCCCCATCATAATGAAAACACCCCACCCACGAGCCATTGAAGCAGCCACAAAATATTTC

TTAACTCAAGCTACAGCTTCTGCCTTAATACTATTTTCATGCCTCATCAACGCCCTGCAA

ACTGGAGAATGAAATATCAACAACCCCCACAATTTAATAACAACTCCCCTGTCCCTAGCT

ATTATAATAAAATTAGGACTAGCCCCCCTACACTTTTGACTGCCAGAAGTTCTCCAAGGA

ACCCCCCTGTCAACAGGACTCATCTTATCTACCTGACAAAAAATTCCCCCCATAATCCTC

CTGCTTCAAATTTCCCCCAACATTAACCTAAGCCTAACAATTATCATGGGCCTTACTTCA

ATTTTAATCGGGGGCTGAGGCGGGATCGGCCAAACCCAACTTCGAAAAATCATAGCCTTC

TCCTCAATTGGCCACCTTGGATGAATCATCCTCATCTTAAAGCTTAACCCACAGCTCTCC

CTGTTTAGCTTCATCCTATACATCATTATAACAACTGCTATATTCCTTTCACTAATTATG

CTCTCCACCACAAAAATATCACAAATCTCAATCTCATGAACTAAAAACCCTGCCATAATA

ACAACCACCATGCTCATCCTCCTCTCTCTAGCAGGACTACCCCCTCTC

>CIB20130533

ATTAACCCACTCGCCCTTATAGTATTTCTTATTAGCCTTGCCATCGGAGCCACCACTACT

ATATTAAGTCACCACTGACTTCTTGCCTGAATCGGATTAGAGATTAATACCTTAGCTTTA

CTCCCCATCATAATGAAAACACCCCACCCACGAGCCATTGAAGCAGCCACAAAATATTTC

TTAACTCAAGCTACAGCTTCTGCCTTAATACTATTTTCATGCCTCATCAACGCCCTGCAA

ACTGGAGAATGAAATATCAACAACCCCCACAATTTAATAACAACTCCCCTGTCCCTAGCT

ATTATAATAAAATTAGGACTAGCCCCCCTACACTTTTGACTGCCAGAAGTTCTCCAAGGA

ACCCCCCTGTCAACAGGACTCATCTTATCTACCTGACAAAAAATTCCCCCCATAATCCTC

CTGCTTCAAATTTCCCCCAACATTAACCTAAGCCTAACAATTATCATGGGCCTTACTTCA

ATTTTAATCGGGGGCTGAGGCGGGATCGGCCAAACCCAACTTCGAAAAATCATAGCCTTC

TCCTCAATTGGCCACCTTGGATGAATCATCCTCATCTTAAAGCTTAACCCACAGCTCTCC

CTGTTTAGCTTCATCCTATACATCATTATAACAACTGCTATATTCCTTTCACTAATTATG

CTCTCCACCACAAAAATATCACAAATCTCAATCTCATGAACTAAAAACCCTGCCATAATA

ACAACCACCATGCTCATCCTCCTCTCTCTAGCAGGACTACCCCCTCTC

>CIBHN201108149

ATAAACCCTTTTGCCCTAATAGTTTTCCTTATCAGCCTTGCCGTAGGAACAACCGTTGCC

CTATCAAGTCATCACTGACTCCTTGCCTGAATCGGATTAGAAATCAATACCTTGGCCCTA

CTCCCGGTCATAACAAAAACGCCACATCCACGAGCCATTGAGGCAGCCACAAAATATTTT

TTAACCCAAGCCACTGCCTCCGCTTTAATATTATTTTCGTGCCTTATTAACGCACTACAA

ACCGGAGAATGAGATATCACCATCCCCCCCAATTTAATGATAAACCCCCTTTCCATCGCC

CTTATAATAAAACTAGGATTAGCCCCCCTACACTTCTGACTACCAGAAGTCCTCCAAGGA

GTTTCCCTCTCAACAGGATTAATCCTATCAACCTGACAAAAAATTCCTCCAATAATTCTC

CTCTTCCTAACTTCTCATAATATTAACCTGAACTTAGCAATTGTTTTAGGCTCTATCTCA

ATCTTAGTCGGAGGCTGAGGCGGAATCAGCCAAACCCAGCTTCGAAAAATTATAGCCTTC

TCCTCCATCGGCCACCTCGGATGAATTATTCTTATTTTAAAACTTAACCCACAACTCTCC

ATCTTCAGCTTTATTTTATATATACTTATAACAACTGCCGTATTCCTTTCACTCATTACC

CTATCTACCACAAAAATATCACAAATTTCAATCTCCTGAACTAAAAACCCAACCGTAACT

ACGGCCACTATAGTTACTCTTCTCTCTCTAGCAGGACTCCCACCCCTC

>CIBFJS20150501004

ATAAACCCTTTTGCCCTAATAGTTTTCCTTATCAGCCTTGCCGTAGGAACAACCGTTGCC

CTATCAAGTCACCACTGACTCCTTGCCTGAATCGGATTAGAAATCAACACCTTGGCCCTA

CTCCCGGTCATAACAAAAACGCCACATCCACGAGCCATTGAGGCAGCCACAAAATATTTT

TTAACCCAAGCCACTGCCTCCGCTTTAATATTATTTTCGTGCCTTATTAACGCACTACAA

ACCGGAGAATGAGACATCACCATCCCCCCCAATTTAATAATAAACCCCCTCTCCATCGCC

CTTATAATAAAACTAGGATTAGCCCCACTACACTTCTGACTACCAGAAGTCCTCCAAGGA

GTTTCCCTCTCAACAGGATTAATTCTATCAACCTGACAAAAAATTCCTCCAATAATTCTC

CTCTTCCTAACTTCTCATAATATTAACCTGAACTTAGCAATTGTTTTAGGCTCTATCTCA

ATCTTAGTCGGAGGCTGAGGCGGAATCAGCCAAACCCAGCTTCGAAAAATTATAGCCTTC

TCCTCCATCGGCCACCTCGGATGAATTATTCTTATTTTAAAACTTAATCCACAACTCTCC

ATCTTCAGCTTTATTTTATATATACTTATAACAACTGCCGTATTCCTTTCACTCATTACC

CTCTCTACCACAAAAATATCACAAATTTCAATCTCCTGAACTAAAAACCCAACCGTAACT

ACGGCCACTATAGTTACTCTTCTCTCTCTAGCAGGACTCCCGCCCCTC

>CIBFJS20150501006

ATAAACCCTTTTGCCCTAATAGTTTTCCTTATCAGCCTTGCCGTAGGAACAACCGTTGCC

CTATCAAGTCACCACTGACTCCTTGCCTGAATCGGATTAGAAATCAACACCTTGGCCCTA

CTCCCGGTCATAACAAAAACGCCACATCCACGAGCCATTGAGGCAGCCACAAAATATTTT

TTAACCCAAGCCACTGCCTCCGCTTTAATATTATTTTCGTGCCTTATTAACGCACTACAA

ACCGGAGAATGAGACATCACCATCCCCCCCAATTTAATAATAAACCCCCTCTCCATCGCC

CTTATAATAAAACTAGGATTAGCCCCACTACACTTCTGACTACCAGAAGTCCTCCAAGGA

GTTTCCCTCTCAACAGGATTAATTCTATCAACCTGACAAAAAATTCCTCCAATAATTCTC

CTCTTCCTAACTTCTCATAATATTAACCTGAACTTAGCAATTGTTTTAGGCTCTATCTCA

ATCTTAGTCGGAGGCTGAGGCGGAATCAGCCAAACCCAGCTTCGAAAAATTATAGCCTTC

TCCTCCATCGGCCACCTCGGATGAATTATTCTTATTTTAAAACTTAATCCACAACTCTCC

ATCTTCAGCTTTATTTTATATATACTTATAACAACTGCCGTATTCCTTTCACTTATTACC

CTCTCTACCACAAAAATATCACAAATTTCAATCTCCTGAACTAAAAACCCAACCGTAACT

ACGGCCACTATAGTTACTCTTCTCTCTCTAGCAGGACTCCCGCCCCTC

>CIBLS20140616004

ATTAACCCCCTTGCCCTAATAGTCTTTCTTATCAGCCTTGCCTTAGGAACGACCATTACC

TTATCAAGTCACCACTGGCTCCTTGCCTGAATCGGATTAGAAATTAACACCTTGGCCCTA

CTCCCAATTATAACAAAAACACCGCATCCACGGGCCATTGAAGCAGCCACAAAATACTTT

TTAACCCAGGCCACAGCTTCCGCTTTAATGCTATTTTCATGCCTTATTAGTGCACTACAA

GCCGGAGAATGAGACATTGTTACCCCCCCCAATTTGATATTAAACCCTCTTTCCATCGCT

CTTATAATAAAACTAGGATTAGCCCCCCTACACTTCTGATTACCAGAAGTCCTCCAAGGA

ATTTCCCTCTCAACAGGACTGATCTTATCAACCTGACAAAAAATTCCTCCAATAGTCCTC

CTCTTCTTAGTTTCTCACGATATCAGCCTAGACCTAATGATTATTTCAGGCCTTATTTCA

ATCTTAATCGGAGGCTGAGGAGGGATCGGCCAAACCCAGCTTCGAAAAATCATAGCCTTC

TCCTCCATTGGCCACCTCGGATGAATTATTCTCGTTTTAAAACTTGACCCCCAACTCTCC

CTCTTCAGCTTTATCTTATATACTATTATAACGACTGCCATATTCCTTTCGCTCATTATT

CTTTCTGCCACAAAAATATCGCAAATCTCAATTTCCTGAGCTAAAAACCCCGCCCTAGCC

ACAACTGCTATAATTAATCTCCTTTCCCTAGCAGGACTTCCACCTCTC

>CIBLS20140616006

ATTAACCCCCTTGCCCTAATAGTCTTTCTTATCAGCCTTGCCTTAGGAACGACCATTACC

TTATCAAGTCACCACTGGCTCCTTGCCTGAATCGGATTAGAAATTAACACCTTGGCCCTA

CTCCCAATTATAACAAAAACACCGCATCCACGGGCCATTGAAGCAGCCACAAAATACTTT

TTAACCCAGGCCACAGCTTCCGCTTTAATGCTATTTTCATGCCTTATTAGTGCACTACAA

GCCGGAGAATGAGACATTGTTACCCCCCCCAATTTGATATTAAACCCTCTTTCCATCGCT

CTTATAATAAAACTAGGATTAGCCCCCCTACACTTCTGATTACCAGAAGTCCTCCAAGGA

ATTTCCCTCTCAACAGGACTGATCTTATCAACCTGACAAAAAATTCCTCCAATAATCCTC

CTCTTCTTAATTTCTCACGATATCAGCCTAGACTTAATGATTATTTCAGGCCTTATTTCA

ATCTTAATCGGAGGCTGAGGAGGGATCGGCCAAACCCAGCTTCGAAAAATCATAGCCTTC

TCCTCCATTGGCCACCTCGGATGAATTATTCTCGTTTTAAAACTTGACCCCCAACTCTCC

CTCTTCAGCTTTATCTTATATACTATTATAACGACTGCCATATTCCTTTCGCTCATTATT

CTTTCTGCCACAAAAATATCGCAAATCTCAATTTCCTGAGCTAAAAACCCCGCCCTAGCC

ACAACTGCTATAATTAATCTCCTTTCCCTAGCAGGACTTCCACCTCTC

>CIBLS20140818005

ATTAATCCTCTCGCCATAATAATATTCCTTACTAGCCTTGCCATCGGAACCACCATTACT

CTATCAAGCCACCACTGACTTCTTGCCTGAATCGGACTAGAAATCAATACCTTAGCCCTA

CTCCCCATCATAACGAAAACTCCTCACCCACGAGCCATTGAAGCAGCTACAAAATATTTC

CTAACCCAAGCCACAGCTTCTGCCTTAATATTATTTTCATGCCTTATCAACGCCATACAA

GCTGGAGAATGAGATATTAACACCCCCTCAAATTTAATAACAAACTCGCTATCTATCGCT

ATTATAATGAAATTAGGATTAGCCCCCATACACTTTTGATTGCCAGAAGTACTACAAGGA

ATCTCCCTTGTAACTGGGCTCATCTTGTCTACTTGACAAAAAATCCCCCCGATAATCCTC

CTGTTTCAAATTTCCCATAATATTAACCTAAGCCTAACAGTCATCCTAGGCCTTACTTCA

GTTTTAATCGGCGGCTGAGGCGGAATTGGCCAAACCCAAATCCGAAAAATTATGGCCTTC

TCCTCTATCGGCCACCTTGGGTGAATTATTATCATCTTAAAACTTAACCCACAGCTCTCC

TTATTTAACTTCATTTTATATACAATTATGACAACTGCCATATTCCTCTCAATAATTATG

CTATCTGCTACAAAAATATCACAAATTTCAACTTCCTGAACTAAAAACCCCGCCCTAACC

ACAACCACCATGCTTGTCCTCCTCTCCCTAGCCGGACTCCCACCTCTC

>CIBGD201108030

ATTAATCCTCTCGCCATAATAATATTCCTTACTAGCCTTGCCATCGGAACCACCATTACT

CTATCAAGCCACCACTGACTTCTTGCCTGAATCGGACTAGAAATCAATACCTTAGCCCTA

CTCCCCATCATAACGAAAACTCCTCACCCACGAGCCATTGAAGCAGCTACAAAATATTTC

CTAACCCAAGCCACAGCTTCTGCCTTAATATTATTTTCATGCCTTATCAACGCCATACAA

GCTGGAGAATGAGATATTAACACCCCCTCAAATTTAATAACAAACTCGCTATCTATCGCT

ATTATAATGAAATTAGGATTAGCCCCCATACACTTTTGATTGCCAGAAGTACTACAAGGA

ATCTCCCTTGTAACTGGGCTCATCTTGTCTACTTGACAAAAAATCCCCCCGATAATCCTC

CTATTTCAAATTTCCCATAATATTAACCTAAGCCTAACAGTCATCCTAGGCCTTACTTCA

GTTTTAATTGGCGGCTGAGGCGGAATCGGCCAAACCCAAATCCGAAAAATTATGGCCTTC

TCCTCTATCGGCCACCTTGGGTGAATTATTATCATCTTAAAACTTAACCCACAGCTCTCC

TTATTTAACTTCATTTTATATACAATTATGACAACTGCCATATTCCTCTCAATAATTATG

CTATCTGCTACAAAAATATCACAAATTTCAACTTCCTGAACTAAAAACCCCGCCCTAACC

ACAACCACCATGCTTGTCCTCCTCTCCCTAGCCGGACTCCCACCTCTC

>CIBFJS20150502002

ATTAATCCTCTCGCCATAATAATATTCCTTACTAGCCTTGCCATCGGAACCACCATTACT

CTATCAAGCCACCACTGACTTCTTGCCTGAATCGGACTAGAAATCAATACCTTAGCCCTA

CTCCCCATCATAACGAAAACTCCTCACCCACGAGCCATTGAAGCAGCTACAAAATATTTC

CTAACCCAAGCCACAGCTTCTGCCTTAATATTATTTTCATGCCTTATCAACGCCATACAA

GCTGGAGAATGAGATATTAACACCCCCTCAAATTTAATAACAAACTCGCTATCTATCGCT

ATTATAATGAAATTAGGATTAGCCCCCATACACTTTTGATTGCCAGAAGTACTACAAGGA

ATCTCCCTTGTAACTGGGCTCATCTTGTCTACTTGACAAAAAATCCCCCCGATAATCCTC

CTGTTTCAAATTTCCCATAATATTAACCTAAGCCTAACAGTCATCCTAGGCCTTACTTCA

GTTTTAATCGGCGGCTGAGGCGGAATTGGCCAAACCCAAATCCGAAAAATTATGGCCTTC

TCCTCTATCGGCCACCTTGGGTGAATTATTATCATCTTAAAACTTAACCCACAGCTCTCC

TTATTTAACTTCATTTTATATACAATTATGACAACTGCCATATTCCTCTCAATAATTATG

CTATCTGCTACAAAAATATCACAAATTTCAACTTCCTGAACTAAAAACCCCGCCCTAACC

ACAACCACCATGCTTGTCCTCCTCTCCCTAGCCGGACTCCCACCTCTC
